# Prevalence and Severity of Lower Gastrointestinal Symptoms amongst Non-Dialysis Chronic Kidney Disease Patients: A Systematic Review and Meta-Analysis

**DOI:** 10.3390/jcm11216363

**Published:** 2022-10-28

**Authors:** Jakub Ruszkowski, Katarzyna Majkutewicz, Zbigniew Heleniak, Jacek M. Witkowski, Alicja Dębska-Ślizień

**Affiliations:** 1 Department of Pathophysiology, Faculty of Medicine, Medical University of Gdańsk, 80-211 Gdańsk, Poland; 2 Department of Nephrology, Transplantology and Internal Medicine, Faculty of Medicine, Medical University of Gdańsk, 80-214 Gdańsk, Poland; 3 Student Scientific Circle, Department of Nephrology, Transplantology and Internal Medicine, Faculty of Medicine, Medical University of Gdańsk, 80-214 Gdańsk, Poland

**Keywords:** conservative management, digestive symptoms, symptomatology

## Abstract

Chronic kidney disease (CKD) patients experience a wide range of symptoms that deteriorate their health-related quality of life (HRQoL). We aimed to estimate the prevalence and severity of lower gastrointestinal (GI) symptoms in non-dialysis CKD adult outpatients, and to summarize the relationships between these symptoms and HRQoL, laboratory test results, and clinical data. The protocol of the study was preregistered (PROSPERO CRD42021255122). We searched MEDLINE, Scopus, Web of Science, and grey literature sources from the databases’ inception up until 27 November 2021. Wide citation chasing was conducted. Single proportions (prevalence of functional constipation, self-reported constipation, diarrhea, abdominal bloating, fecal incontinence, and abdominal/rectal pain) were pooled using generalized linear mixed models. A total of 37 studies with 12,074 patients were included. We found that lower GI symptoms, especially self-reported abdominal bloating [CKD G1–2: 48.45% (95% CI: 43.5–53.4%; 2 studies); G3: 46.95% (95% CI: 45.0–48.9%; 2 studies), G4–5: 36.1% (95% CI: 25.4–48.5%; 8 studies)] and constipation [CKD G1–2: 31.8% (95% CI: 13.9–54.9%); G3: 29.8% (95% CI: 21.2–40.1%; 4 studies); G4–5: 38.8% (95% CI: 30.9–47.4%); 22 studies)], were common in non-dialysis CKD patients. The severity of the symptoms was limited. Self-reported constipation was most consistently associated with worse HRQoL, whereas hard stool consistency was associated with higher uremic toxins levels. To conclude, since lower GI symptoms are common in CKD, using symptom questionnaires that do not take them into account cannot provide full insight into the patient’s experience. Further studies are needed to cover identified knowledge gaps, including the exploration of the pathophysiology of GI symptoms in CKD with multi-omics data.

## 1. Introduction

Chronic kidney disease (CKD), defined as abnormalities of kidney structure or function present for ≥3 months, with implications for health, is a common condition affecting ca. 9.1% of the global population [1]. A decline in kidney function disrupts several physiological processes and adversely affects multiple organs [2]. Therefore, CKD patients suffer from such conditions as anemia, cardiovascular disease, immune dysfunction, malnutrition, mineral and bone disorder, and water–electrolyte imbalance [2]. As a consequence, CKD patients experience a wide range of symptoms that deteriorate both their physical and mental health-related quality of life (HRQoL) [3,4]. The overall burden of CKD is substantial; it is estimated that CKD leads to a loss of 451.3 years of full health per 100,000 population due to premature mortality or disability (age-standardized disability-adjusted life year (DALY) rate) and is the 12th leading cause of death out of 133 conditions with an estimated 2.6 million deaths in total in 2017 [1]. Increased cardiovascular mortality in CKD is an essential contributor to these appalling statistics [1,2]. 

Among a plethora of CKD symptoms, lower gastrointestinal (GI) ones are gaining more and more attention in recent years. This is especially true for constipation [5]. Recent epidemiological registry-based studies revealed that constipation is associated with higher incidence rates of CKD and kidney failure [6,7]. Surprisingly, how CKD progression affects the lower GI symptom burden has not yet been systematically studied. Intuitively, as CKD progresses, both the prevalence and severity of GI symptoms should increase. This is a crucial consideration, as several GI symptoms were found to be associated with deteriorated HRQoL in both the general and CKD populations [8,9].

While there are systematic reviews assessing the prevalence of several symptoms in CKD patients [3,10], none are dedicated to a comprehensive analysis of lower GI symptoms in non-dialysis-dependent patients. This patient population deserves special attention because they constitute more than 99% of all CKD patients [1]; moreover, non-dialyzed CKD patients may have different risk factors and implications of GI symptoms than patients undergoing renal replacement therapy [10]. Because patients can understand symptoms differently than health practitioners, a careful approach is required to estimate the prevalence and implications of each from the spectrum of GI symptoms [11,12]. 

The primary objective of the review was to estimate the prevalence and severity of lower GI symptoms in non-dialysis CKD patients worldwide. Additionally, we aimed to identify the relationships between these symptoms and HRQoL, laboratory test results, and clinical data.

## 2. Materials and Methods

A protocol for this systematic review was registered with the International Prospective Register of Systematic Reviews (PROSPERO 2021 CRD42021255122). All deviations from the protocol were listed and explained in Table S1. The article was written in accordance with the Preferred Reporting Items for Systematic Reviews and Meta-Analysis (PRISMA 2020) statement [13].

### 2.1. Eligibility Criteria

We aimed to include all observational studies reporting at least one of the predefined outcomes and recruiting adult outpatients with CKD that were treated without dialysis. There were no language restrictions, nor did we exclude studies based on the date of publication or sample size. We specified and explained the eligibility criteria in Supplementary Materials, Methods S1. In this article, we show data on patients that did not receive kidney transplants; articles regarding patients after kidney transplantation will be reviewed in a separate article.

### 2.2. Information Sources and Search Strategy

To identify the studies of interest, we systematically searched MEDLINE (via PubMed), Scopus, Web of Science Core Collection, Korean Journal Database (via Web of Science), and SciELO (via Web of Science). The databases were searched from inception, and all languages were considered. The initial search was performed on 24 May 2021, and it was rerun on 27 November 2021. Additional searches of the database Open Dissertations (via EBSCO; on 24 May 2021 and 27 November 2021) and American Society of Nephrology conference abstracts from the last ten years (ASN Kidney Week 2011–2021) were conducted.

Literature search strategies consisted of two parts: the first dedicated to finding articles about populations of interest, and the second dedicated to the outcomes of interest. To realize the first aim, we modified the high-sensitivity CKD filter developed by Iansavichus et al. [14] (removal of elements referring to dialysis and hyperphosphatemia). To find studies reporting the outcomes, we used medical subject headings (MeSH) or other subject terms, keywords, and synonyms related to outcomes of interest. Full search strategies for each of the databases were shown in the Table S2.

The CitationChaser Shiny app. [15] was used to perform forward citation chasing of articles presenting symptom questionnaires of interest on 14 November and 26 December 2021 (Table S3). Finally, we used the CitationChaser Shiny app. [15] also for both backward and forward citation chasing of the included studies on 22 January 2022.

### 2.3. Study Selection, and Data Collection Process

All search results were imported into the Rayyan QCRI reference manager web application for deduplication and screening [16]. JR excluded duplicates. Two investigators (Ruszkowski, J and either Majkutewicz, K or Heleniak, Z) independently screened all article titles and abstracts for eligibility (blind mode). Full texts of the articles that fulfilled the initial screening criteria were acquired and reviewed for subsequent inclusion, against the eligibility criteria.

Data were independently extracted by two authors (Ruszkowski, J and either Majkutewicz, K or Heleniak, Z) in a blinded standardized manner using the Systematic Review Data Repository Plus (SRDR+; https://srdrplus.ahrq.gov (accessed on 18 September 2022)). Items of electronic extraction form are listed in Table S4. All the differences were settled by discussion between all researchers. Both main manuscripts and all available supplementary materials of the included studies were evaluated. If the outcome was measured at multiple time points, we extracted data from the first measurement. In the case of missing crucial data, we tried to contact authors via e-mail or ResearchGate. Ruszkowski, J exported data from SRDR+ as an xlsx file and processed data using both Pandas 1.3.5 [17] and manually (Excel, Microsoft Office 365 (Redmond, WA, USA)).

### 2.4. Study Risk of Bias and Reporting Bias Assessment

Two reviewers (Ruszkowski, J and either Majkutewicz, K or Heleniak, Z) assessed the quality of all included studies using the Joanna Briggs Institute (JBI) Critical Appraisal Checklist for Prevalence Studies [18]. Assessment of “reporting biases” (e.g., publication bias) are described in Supplementary Materials, Methods S2.

### 2.5. Synthesis Methods and Certainty Assessment

Since the burden of disease- and treatment-related symptoms increases with the progression of CKD [19], we decided to meta-analyze all outcomes separately in the subgroups as follows: early CKD (G1 and G2, i.e., estimated glomerular filtration rate (eGFR) ≥ 60 mL/min per 1.73 m^2^), moderate CKD (G3, i.e., eGFR 30–59 mL/min/1.73 m^2^), and advanced CKD (G4 and G5, i.e., eGFR < 30 mL/min/1.73 m^2^). We planned to conduct separate meta-analyses according to albuminuria categories; however, data on albuminuria were not reported in the included studies. Location of data collection (countries) were grouped into six World Health Organization (WHO) regions.

Prevalence and severity data were synthesized when available from at least 2 studies. Results for each prevalence outcome expressed as single proportions were pooled using generalized linear mixed models (GLMM). Specifically, the mean prevalence across subsets of studies was estimated with a random intercept logistic regression model (logit transformation) via the maximum-likelihood approach using the *metaprop* function from the ‘meta’ package (version 5.2-0) [20]. If at least 3 studies were available for a specific outcome–population pair, a 95% prediction interval (95% PI) for the proportion in a new study was calculated. To calculate 95% PI, ‘meta’ uses the *t*-distribution with (*n* − 2) degrees of freedom where *n* corresponds to the number of studies in the meta-analysis [20,21]. To compare the prevalence between subgroups, a meta-analysis of within-study odds ratios was conducted using a random effects model with a restricted maximum-likelihood (REML) estimator for τ^2^.

Since data on severity were collected as ordered discrete variables (e.g., “mild”, “severe”, “overwhelming”) that can be perceived as more informative for the larger audience than a continuous estimator of severity, we preferred to meta-analyze the prevalence of each category of severity (multi-category prevalence [22]). It was performed using a MetaXL implementation of a random-effects meta-analysis with the Freeman–Tukey double arcsine transformation of proportions with the DerSimonian and Laird estimator of variance (it is the only available estimator) [22]. The same method was used to meta-analyze data on the number of bowel movements (BMs) per week, because it was reported in studies as categorical data (less than 3; at least 3 but less than 7; more than 7). Alternatively, if severity was available only as a continuous variable, a random-effects meta-analysis of single means was planned to be conducted.

The results for prevalence data (proportions) were multiplied by 100% to be interpretable as a percentage of the population. Forest plots were generated using the *forest* function from the ‘meta’ package [20]. Associations/correlations between each of the lower GI symptoms/syndromes and HRQoL/laboratory test results/clinical data were presented via the structured tabulation of results across studies and discussed. The meta-analyses of such associations/correlations were not planned, nor performed.

Confidence interval calculation, subgroup analysis, sensitivity analysis, and certainty assessment are detailed in Supplementary Materials, Methods S3.

## 3. Results

### 3.1. Study Selection

**Figure 1:**
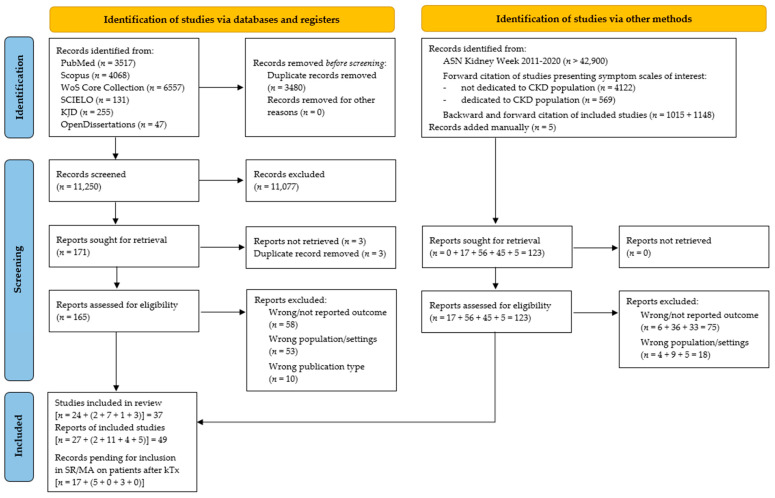
PRISMA 2020 flow diagram.

Figure 1 shows the study selection process. We found 14,730 records through database searching. After duplicates removal and records screening, we reviewed 165 full-text documents and included 27 reports of 24 studies. We identified an additional 6834 records using citation chasing of both included studies and articles introducing selected questionnaires; after reviewing 118 full-text documents, we included 17 papers. Additionally, we added five papers manually. We listed reasons for exclusion in Table S5. Taken together, we included 49 reports of 37 studies in our systematic review. Moreover, 25 records are pending for inclusion in our future systematic review dedicated to the same outcomes in patients after kidney transplantation.

### 3.2. Study Characteristics and Risk of Bias

Basic characteristics of the included studies are summarized in Table 1. All except seven studies had a cross-sectional design: five were prospective cohorts [23,24,25,26,27], one a retrospective cohort [28], and one was a case-control study [29]. There were studies from each of the WHO regions: 12 from the Western Pacific Region (Australia, Brunei, China, Japan, Malaysia, and South Korea), 11 from the European Region (Belgium, Denmark, Germany, Italy, the Netherlands, Poland, Spain, Sweden, Turkey, and the United Kingdom), 6 from the Region of the Americas (Brazil, Mexico, United States), 4 from the South-East Asian Region (all from Sri Lanka), 2 from the African Region (both from Nigeria), and 2 from the Eastern Mediterranean Region (Iraq, Saudi Arabia). Besides reports written in English, there were 4 studies reported in other languages (German [30], Spanish [27,28,31]). 

The vast majority of studies reported data collected in CKD G4–5 patients (30 studies), whereas far fewer studies reported data on GI symptomatology in the early stages of CKD (6 in G1–2 [9,32,33,34,35,36]; 10 in G3 [9,32,33,34,35,36,37,38,39,40]). Subgroup characteristics were described in Supplementary Materials, Results S1.

The sample frame was poorly described, questionable, or clearly inappropriate to address the target general CKD population in 25 (68%) studies. Since the objectives of individual studies varied from ours, the studies used too-restrictive inclusion criteria (e.g., only CKD G5 patients receiving palliative treatment) or too-wide exclusion criteria (e.g., suffering from diabetes mellitus or heart failure, or using such drugs as beta-blockers and tricyclic antidepressants [29]; exclusion criteria of all studies are listed in Table S6) to obtain a representative CKD population. Moreover, participants were recruited in a recommended way in only 13 (35%) studies; other studies used convenience sampling or did not clearly report the method of sampling. The sample size was judged as adequate in only 6 (16%) studies. All of these limitations in the included studies may increase the risk of bias in the observed results. On the other hand, the risk of measurement/classification bias was limited: 24 (65%) studies used validated questionnaires to collect data on GI symptoms, and 20 (54%) studies collected data in a standard, reliable way for all participants (the reliability of data collection in the next 15 studies was unclear, not necessarily low). The full risk of bias assessment is shown in Table S7.

**Table 1:** Included studies.

Author, Reference	Extracted Populations			Outcomes	Study Design	Country	Study Period
	CKD Stage (N)	Age	Male (%)				
Gordon, S.J.; et al. [41]	G4–5 (4)	50	75.0%	D ^a^	cross-sectional	USA	(1976)
Yapa, H.E.; et al. [39]	G3b (224)	55.96	60.7%	C, C-s, D, D-s	cross-sectional	Sri Lanka	2018–2019
	G4–5 (443)	61.34	65.5%				
Muhd Ariffin, N.F.; et al. [42]	G5 (50)	NR	NR	C, AP, B, D	cross-sectional	Brunei	(2016)
Ramos, C.I.; et al. [40]	G3 (6)	55.83	83.3%	BSFS, FC	cross-sectional	Brazil	2015–2016
	G4–5 (36)	59.58	52.8%				
Trimingham, C.; et al. [12]	General population of non-dialysis CKD patients (95)	NR	47.4%	BSFS, AP, B, BM	cross-sectional	Australia	(2018)
Sanya, E.O. and Ogunniyi, A. [43]	General population of non-dialysis CKD patients (60)	39	NR	C, D	cross-sectional	Nigeria	2000–2000
Saini, T.; et al. [44]	G4–5 (11)	67	72.7%	C, B, D	cross-sectional	UK	2005–2005
Ohkuma, T.; et al. [45]	General population of non-dialysis patients with diabetic kidney disease (2245)	NR	NR	BM	cross-sectional	Japan	2008–2010
Ruszkowski, J.; et al. [9,46]	G1–2 (16)	49.6	37.5%	BSFS, FC, AP, AP-s, B, B-s, BM	cross-sectional	Poland	2018–2019
	G3 (69)	66.8	52.2%				
	G4–5 (26)	63.5	76.9%				
Quintal-Medina, I.A.; et al. [27]	G5 (70)	59	55.7%	C, C-s, D, D-s	prospective cohort	Mexico	2018–2018
Meade, A.; et al. [47]	G4–5 (134)	64.6	64.9%	BSFS, B, BM, RP, FI	cross-sectional	Australia	2017–2018
Wizemann, V. and Benz, U. [30]	G5 (20)	NR	NR	C, AP, B, D	cross-sectional	Germany	(1978)
Zhang, X.; et al. [32]	G1–2 (370)	51.6	57.0%	B	cross-sectional	USA	2003–2008
	G3 (2541)	59.2	56.7%				
	G4–5 (688)	58.8	47.8%				
Gryp, T.; et al. [33,48]	G1–2 (37)	51.3	54.05%	BSFS	cross-sectional	Belgium	(2020)
	G3 (44)	64	65.9%				
	G4–5 (33)	69.5	72.7%				
Miskulin, D.C. and the HALT-PKD studies investigators [49,50]	ADPKD G1–4 (1043)	41.8	50.1%	AP, AP-s	cross-sectional	USA	2006–2009
Windahl, K. and the EQUAL study inves-tigators [23,51,52,53]	G4–5 (1205)	76 ^b^	64.6% ^b^	C, D, C-s, D-s	prospective cohort	Germany, Italy, the Netherlands, Poland, Sweden, UK	2012–2018
Ducharlet, K.; et al. [24]	G4 (31)	71	74%	C, D, C-s, D-s	prospective cohort	Australia	2014–2014
Grove, B.E.; et al. [38]	G3 (141)	65	63.1%	C, D, C-s, D-s	cross-sectional	Denmark	2019–2019
	G4–5 (92)	68	72.8%				
Onodugo, O.D.; et al. [54]	G5 (80)	41.5	48.8%	C ^a^, D ^a^	cross-sectional	Nigeria	2015–2015
Allawi, A.A. [29]	G5 (35)	55.4	62.9%	C	case-control	Iraq	2016–2016
Lee, A.; et al. [55]	G5 (21)	64.2	47.6%	C, BSFS, FC	cross-sectional	Australia	(2016)
Dawson, J.; et al. [37]	G3 (8)	80.6	75%	C, D, C-s, D-s	cross-sectional	Australia	2016–2019
	G4–5 (92)	82.4	64.1%				
Abeywickrama, H.M.; et al. [34]	G1–2 (2)	66	100%	D, D-s	cross-sectional	Sri Lanka	2019–2019
	G3 (45)	60.5	73.3%				
	G4–5 (73)	62.6	65.8%				
Lee, S.J. and Jeon, J.H. [35]	G1–2 (22)	61.7	95.5%	C, C-s, D, D-s	cross-sectional	South Korea	2013–2013
	G3 (83)	68.8	62.7%				
	G4–5 (38)	63.7	42.1%				
Yong, D.S.P.; et al. [56]	G5 (45)	73.1	46.7%	C, C-s, B, B-s	cross-sectional	China	2006–2007
Senanayake, S.; et al. [36]	G1–2 (96)	52.5	32.3%	D, D-s	cross-sectional	Sri Lanka	2016–2016
	G3 (163)	57.9	51.5%				
	G4–5 (782)	59.7	68.3%				
Abdel-Kader, K.; et al. [57]	G4–5 (87)	51	65.5%	C, C-s, D, D-s	cross-sectional	USA	2004–2006
Wan Zukiman, W.Z.H.; et al. [58]	G5 (100)	61.0	48%	C, C-s, D, D-s	cross-sectional	Malaysia	2015–2016
Gutiérrez Sánchez, D.; et al. [31,59,60,61]	G4–5 (124)	69.8	70.2%	C, C-s, D, D-s	cross-sectional	Spain	2015–2015
Murtagh, F.E.; et al. [62,63]	G5 (66)	82	48.5%	C, C-s, B, B-s, D, D-s	cross-sectional	England	2005–2006
Turkmen, K.; et al. [25]	Fabry nephropathy (11)	41.3	63.6%	AP	prospective cohort	Turkey	2014–2016
Brennan, F.; et al. [64]	G5 (42)	83	42.9%	C, C-s, D, D-s	cross-sectional	Australia	2010–2012
Murphy, E.L.; et al. [65]	G4–5 (55)	82	47.3%	C, C-s, D, D-s	cross-sectional	UK	2005–2006
Taira, K.; et al. [66]	General population of non-dialysis CKD patients (15)	54.9	73.3%	C, D	cross-sectional	Sri Lanka	2015–2015
Purtell, L.; et al. [26,67]	G4–5 (46)	78.3	39.1%	C, C-s, D, D-s	prospective cohort	Australia	2016–2017
de Miguel, C.; et al. [28]	G4–5 (102)	79.6	59.8%	C	retrospective cohort	Spain	1997–2009
Almutary, H.; et al. [68,69]	G4–5 (107)	51.6	55.1%	C, C-s, D, D-s	cross-sectional	Saudi Arabia	2013–2014

^a^ only clinical/lab/HRQoL associations with the outcome were extracted, not the prevalence or severity. ^b^ The median age and percentage of males in the EQUAL study were extracted from the article written by Windahl et al. [23], whereas the number of analyzed participants was larger (data from the authors). Abbreviations: ADPKD, autosomal dominant polycystic kidney disease; AP, self-reported abdominal pain; B, self-reported bloating; BM, frequency of bowel movements; BMI, body mass index; BSFS, stool consistency according to the Bristol stool form scale; C, self-reported constipation; D, self-reported diarrhea; FC, functional constipation; FI, self-reported fecal incontinence; NR, not reported; RP, rectal pain; -s, severity of the symptom.

### 3.3. Constipation: Prevalence and Severity 

#### 3.3.1. Self-Reported Constipation

**Figure 2:**
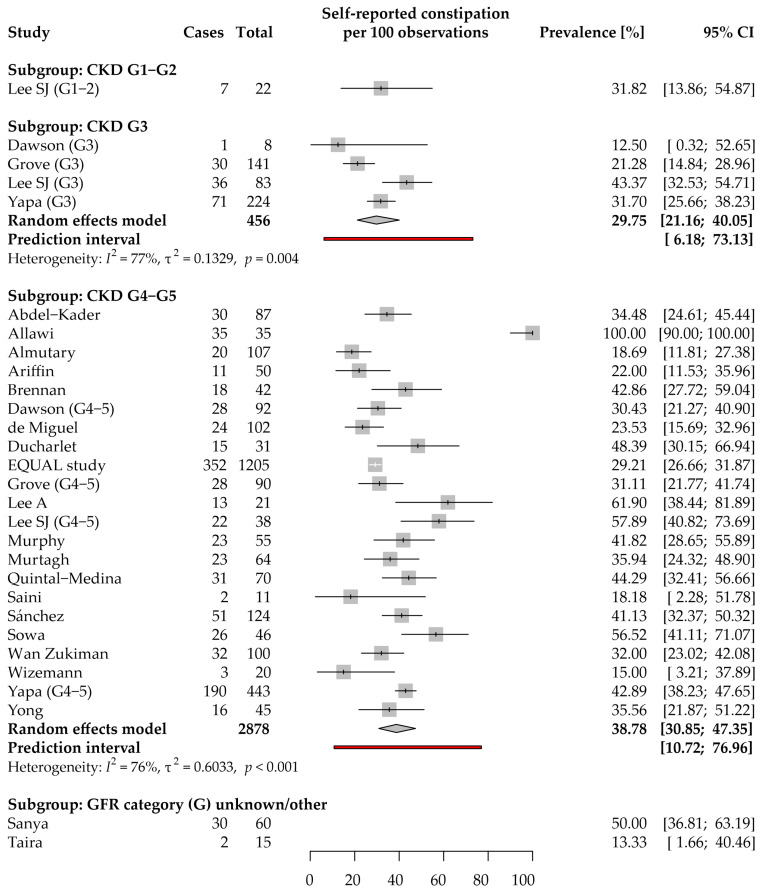
Forest plot with pooled point prevalence estimates for self-reported constipation in chronic kidney disease by stages.

Data on self-reported constipation prevalence in CKD were provided in 24 studies, of which 22 were included in the meta-analyses below (Figure 2). They were conducted primarily in the WHO Western Pacific (9 studies [24,26,35,37,42,55,56,58,64]) and European (8 studies [23,28,30,31,38,44,53,62,65]) Regions. Data were collected using authors’ questionnaires/during anamnesis in the case of 8 studies, and using validated questionnaires: renal version of the Patient Outcome Scale—symptom module (POS-S Renal; 5 studies [24,27,31,35,64]); Integrated Palliative Care Outcome Scale (IPOS)-Renal (3 studies [26,37,39]); Dialysis Symptom Index (DSI) (3 studies [23,53,57,58]); Memorial Symptom Assessment Scale–Short Form (MSAS-SF; 2 studies [44,62]).

Only one study reported self-reported constipation prevalence among patients with CKD G1–G2 (31.8%, 95% CI: 13.9–54.9%); therefore, a meta-analysis was not performed for this subgroup [35]. Based on 7 patients reporting constipation in this subgroup, it was estimated that 57.1% (95% CI: 28.6–91.5%) experienced mild, 28.6% (95% CI: 0–62.9%) moderate, 14.3% (95% CI: 0–48.6%) severe, and 0% (95% CI: 0–34.3%) overwhelming severity [35]. 

A total of 4 studies were included in the prevalence analysis for the CKD G3 subgroup [35,37,38,39]. The observed prevalence ranged from 13 to 43% mainly due to between-study variance (τ^2^ = 0.13; *I*^2^ = 77%). The estimated average prevalence was 29.8% (95% CI: 21.2–40.1%). The 95% prediction interval (PI) for the prevalence in new studies ranged from 6.2 to 73.1%. Three studies presented data that could be meta-analyzed for the symptom severity (Table S8). Mild, moderate, severe, and overwhelming severity of constipation was, on average, reported by 37.6% (95% CI: 10.4–69.1%), 40.9% (95% CI: 12.8–72.2%), 15.8% (0–41.2%), and 5.6% (0–23.9%) of patients reporting the symptom, respectively. 

A total of 22 studies enrolling 2878 patients were included in the analysis for the CKD G4–5 subgroup [24,26,27,28,29,30,31,35,37,38,39,42,44,53,55,56,57,58,62,64,65,66]. The observed prevalence ranged from 15 up to 100%. The estimated average prevalence was 38.8% (95% CI: 30.9–47.4%). Observed heterogeneity in the reported results came from the substantial between-study variance (τ^2^ = 0.60; *I*^2^ = 76%) rather than sampling. A subgroup analysis revealed that it may have partially resulted from the difference in the location of data collection (*p* = 0.02); studies conducted in the European Region (31.4%; 95% CI: 26.8–36.5%) tended to report a lower prevalence than studies from the Western Pacific or American WHO Regions (Table S9). Neither study period, mean age of participants, nor sex of participants were significantly associated with the reported prevalence. While there was no clear asymmetry in the funnel plot (Peters’ regression test: *p* = 0.22), an analysis of both the Doi plot and the LFK index suggested a minor bias favoring publication of studies with a higher prevalence of self-reported constipation in CKD G4–5 (Figure S1). The 95% PI for the prevalence in new studies ranged from 10.7 to 77.0%. Based on four studies that simultaneously reported the prevalence in both CKD G3 and G4–5 stages, self-reported constipation was more likely to be reported in CKD G4–5 subgroups than in G3 subgroups (odds ratio, OR: 1.67 [95% CI: 1.27–2.19], *p* < 0.001). It cannot be explained by the difference in the mean age of patients (*p* = 0.78; Figures S2 and S3). Eight studies with 360 patients reporting constipation were included in the meta-analysis of severity (Table S8). Mild, moderate, severe, and overwhelming severity of constipation was, on average, reported by 43.8% (95% CI: 32.8–54.4%), 34.4% (95% CI: 24.2–44.9%), 18.2% (10.3–27.2%), and 3.6% (0.4–9.0%) of patients reporting the symptom, respectively. A small part of the studies asked how much the symptom burden distressed the patient (Table S10).

Sixteen studies provided data that related prevalence/severity of self-reported constipation with an HRQoL evaluation, clinical data, or laboratory test results (Table S11). Both the presence and severity of self-reported constipation were associated with a significantly worse HRQoL: both the physical and mental well-being of the CKD patients [35,39,53]. Analyses of symptom clusters led to different results: Lee SJ et al. found that constipation and diarrhea clustered together with the “difficulty sleeping” item into the “neurological and bowel problem” symptom cluster [35], whereas both Gutiérrez Sánchez et al. and Almutary et al. failed to cluster constipation together with other symptoms [31,69]. Interestingly, Dawson et al. found an association between taste disturbances and constipation prevalence [37], while the EQUAL study showed that constipation was an independent predictor of a decline in nutritional status [23]. Finally, Murtagh et al. showed that the prevalence of constipation within a month before death was 1.87 times higher than in the whole baseline CKD group [62,63]; nonetheless, the increase in the prevalence of constipation in CKD patients was evidenced also over the 1-year follow-up period in the EQUAL study [52].

Taken together, these data demonstrate that self-reported constipation is one of the most common lower GI symptoms and it is consistently associated with impaired HRQoL. In the case of patients with CKD G3/G4–5, more than half of constipated patients reported at least moderate severity of the symptom.

#### 3.3.2. Functional Constipation

**Figure 3:**
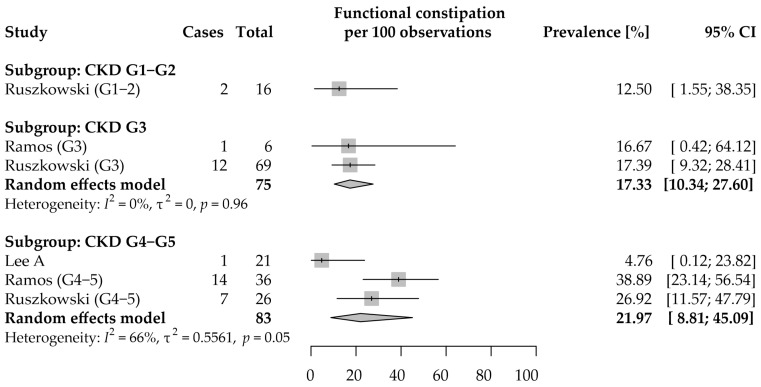
Forest plot with pooled point prevalence estimates for functional constipation in chronic kidney disease by stages.

Data on the prevalence of functional constipation (FC) in CKD were reported in three studies [9,40,55]. Both individual and pooled data are shown in Figure 3. 

Only one study reported the prevalence of FC among patients with CKD G1–2 (12.5%, 95% CI: 1.6–38.4% [9]); therefore, a meta-analysis was not performed for this subgroup. 

A meta-analysis of FC prevalence in the CKD G3 subgroup was conducted using data from two studies [9,40]. The estimated average prevalence was 17.3% (95% CI: 10.3–27.6%). We did not detect significant between-study variance (τ^2^ = 0; *p* = 0.96; *I*^2^ = 0).

A total of 3 studies were included in the analysis for the CKD G4–5 subgroup [9,40,55]. The estimated average prevalence of FC was 22.0% (95% CI: 8.8–45.1%). Data from the included studies were quite homogenous (*p* = 0.05), and it was estimated that two-thirds of the variability was due to between-study variance (τ^2^ = 0.56; *I*^2^ = 66%). There is high uncertainty about the prevalence of FC in future studies (95% PI ranged from 0 to 100%).

All extracted implications of FC were summarized in Table S12. Both Ramos et al. and Ruszkowski et al. failed to show significant associations between gender, age, or body mass index and the presence/prevalence of FC [9,40]. Additionally, Ramos et al. suggested no association between dietary parameters and FC [40], while Ruszkowski et al. showed that taking acetaminophen (paracetamol) was associated with higher, whereas non-steroidal anti-inflammatory drugs with lower, prevalence of FC [9]. Only one study explored (and found) relationships between the presence of FC and both HRQoL and sleep quality [9,46]. Ramos et al. failed to prove a statistically significant association between reporting FC and having higher levels of *p*-cresyl sulfate, one of the uremic toxins that are generated by gut microbiota [40].

In conclusion, even though available data on FC prevalence are quite homogenous, the estimation of the prevalence is uncertain due to data limitations. Further studies are needed to verify the described associations of FC with pharmacotherapy and HRQoL in the CKD population. 

### 3.4. Diarrhea: Prevalence and Severity

**Figure 4:**
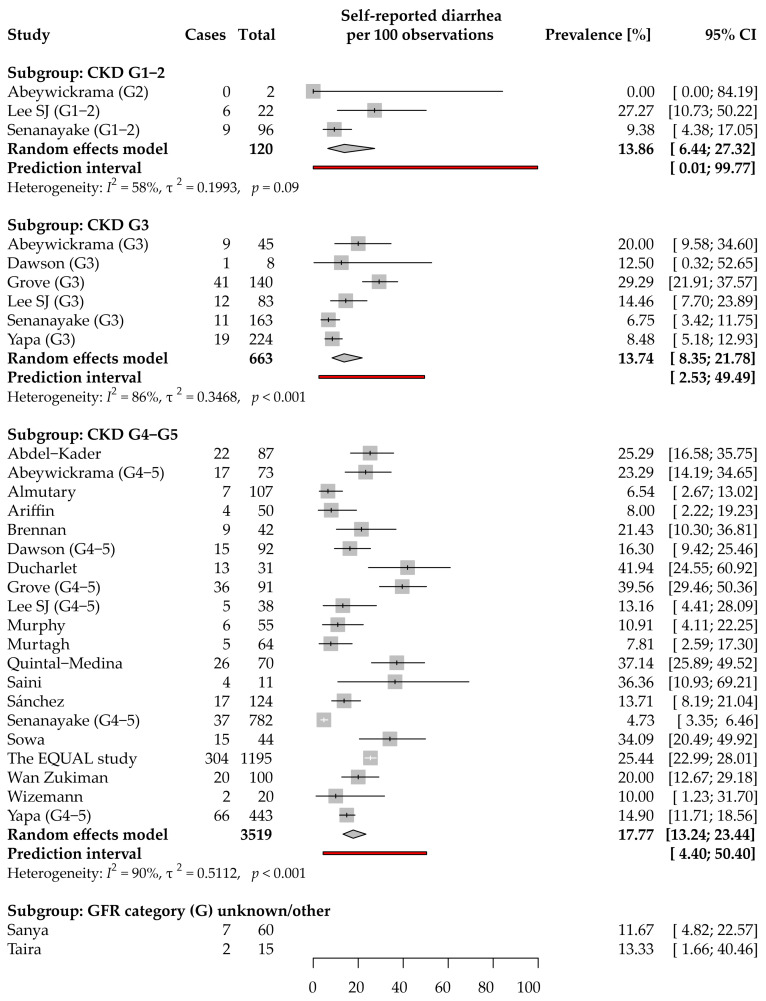
Forest plot with pooled point prevalence estimates for self-reported diarrhea in chronic kidney disease by stages.

Data on self-reported diarrhea prevalence in CKD were provided in 22 studies, of which 20 were included in the meta-analyses below (Figure 4). We did not identify any study reporting the prevalence of functional diarrhea in CKD. Studies reporting self-reported diarrhea were conducted primarily in the WHO Western Pacific (7 studies [24,35,37,42,58,64,67]) and European (6 studies [30,31,38,53,62,65]) Regions, whereas only one study each was conducted in the African (Nigeria [43]) and Eastern Mediterranean (Saudi Arabia [68]) Regions.

Data were collected using authors’ questionnaires/during anamnesis in the case of 4 studies [30,42,43,66], and using validated questionnaires: the POS-S Renal questionnaire and its modifications/translations in 6 studies [24,27,31,35,64,65]; IPOS-Renal in 3 studies [37,39,67]; DSI in 3 studies [53,57,58]; MSAS-SF questionnaire in 2 studies [44,62]; CKD Symptom Index—Sri Lanka (CKDSI-Sri Lanka) in 2 studies [34,36]; and CKD Symptom Burden Index (CKD-SBI [68]) and Renal Disease Questionnaire (RDQ [38]) in one study each.

Three studies were included in the analysis for the CKD G1–2 subgroup [34,35,36]. The estimated average prevalence was 13.9% (95% CI: 6.4–27.3%). While the observed prevalence ranged from 0 to 27%, no significant heterogeneity was detected (*I*^2^ = 58%, τ^2^ = 0.20, *p* = 0.09). The prevalence in this subgroup in future studies is highly uncertain (95% PI: 0–99.8%). Two studies presented data that could be meta-analyzed for the symptom severity (Table S13). Mild, moderate, severe, and overwhelming severity of diarrhea was, on average, reported by 46.8% (95% CI: 15.5–80.9%), 20.0% (95% CI: 0–53.9%), 13.2% (95% CI: 0–40.2%), and 20.0% (95% CI: 0–53.9%) of CKD G1–2 patients reporting the symptom, respectively.

The CKD G3 subgroup analysis included 6 studies (663 patients) [34,35,36,37,38,39]. The estimated average prevalence was 13.7% (95% CI: 8.4–21.8%). The differences in the reported prevalence across studies (7–29%) resulted primarily from between-study variance (τ^2^ = 0.35; *I*^2^ = 86%). The 95% PI for the prevalence ranged from 2.5 to 49.5%. Five studies presented data that could be meta-analyzed for the symptom severity (Table S13). Among CKD G3 patients reporting the symptom, it was of mild, moderate, severe, and overwhelming severity in the case of, on average, 63.0% (95% CI: 43.7–82.4%), 25.2% (95% CI: 9.8–45.4%), 8.0% (95% CI: 0–20.9%), and 3.8% (95% CI: 0–13.6%) of them, respectively.

The CKD G4–5 subgroup analysis included 20 studies (3519 patients) [23,24,26,27,30,31,34,35,36,37,38,39,42,44,53,57,58,62,64,65,68]. The estimated average prevalence was 17.8% (95% CI: 13.2–23.4%). The reported prevalence ranged from 5 to 42% and resulted mainly from the between-study variance (τ^2^ = 0.51; *I*^2^ = 90%). A subgroup analysis revealed that it may have partially resulted from the difference in the location of data collection (*p* < 0.001); studies conducted in the American and Western Pacific Regions reported higher prevalence than studies from the South-East Asian or Eastern Mediterranean WHO Regions (Table S14). Neither study period, average age of participants, nor sex of participants were significantly associated with the reported prevalence. While there was no asymmetry in the funnel plot (Peters’ regression test: *p* = 0.54), an analysis of both the Doi plot and the LFK index suggested a major bias favoring the publication of studies with a lower prevalence of self-reported diarrhea in CKD G4–5 (Figure S4). The 95% PI for the prevalence in new studies ranged from 4.4 to 50.4%. Based on studies that simultaneously reported the prevalence in several CKD stages, no significant differences in the odds of reporting the symptoms were found between CKD G3 patients and either G1–2 (*p* = 0.16) or G4–5 (*p* = 0.23) (Figures S5 and S6). We included 10 studies in the meta-analysis of diarrhea severity in CKD G4–5 (Table S13). Mild, moderate, severe, and overwhelming severity of diarrhea was, on average, reported by 47.1% (95% CI: 33.3–59.7%), 35.6% (95% CI: 23.0–48.2%), 13.5% (95% CI: 5.4–23.6%), and 3.9% (95% CI: 0.1–10.9%) of CKD G4–5 patients reporting the symptom, respectively.

All extracted relationships between self-reported diarrhea and HRQoL, clinical data, or laboratory test results are presented in Table S15. Interestingly, in contrast to the clear picture of the relationship between HRQoL and constipation, there are controversies on this in the case of diarrhea. The EQUAL study did show negative implications of self-reported diarrhea on both the physical and mental components of HRQoL (in a similar way as in the case of constipation) [53]. Contrary to this, Yapa et al. found no significant correlation between diarrhea severity and either the physical or mental components of HRQoL [39]. 

Analyses of symptom clusters led, however, to quite consistent results. According to Almutary et al. nausea and vomiting were core symptoms of the GI symptom cluster across all dimensions; diarrhea was related to this cluster in distress and severity dimensions only [69]. Similarly, Gutiérrez Sánchez et al. reported that diarrhea was clustered together with nausea and vomiting [60]. On the other hand, Lee SJ et al. found that diarrhea and constipation clustered together with the “difficulty sleeping” item into the “neurological and bowel problem” symptom cluster [35].

The pathophysiology of self-reported diarrhea in CKD remains unclear. Two research teams from Nigeria failed to show an association between diarrhea and cardiovascular autonomic neuropathy (autonomic dysfunction) in CKD [43,54]; however, nocturnal diarrhea might be a predictor of this dysfunction [54]. Gordon et al. suggested bile acid abnormalities as a cause of diarrhea in CKD [41], but definitely more studies are needed to confirm this hypothesis.

To sum up, the above-mentioned data indicate that self-reported diarrhea is less common than self-reported constipation in non-dialyzed CKD patients. Unfortunately, no study used Rome criteria, and thus functional diarrhea prevalence remains unknown in CKD. The sophisticated analyses of numerous symptoms suggested that self-reported diarrhea can be somehow related to both nausea and vomiting; however, the understanding of self-reported diarrhea pathobiology in CKD is extremely limited.

### 3.5. Bloating: Prevalence and Severity

**Figure 5:**
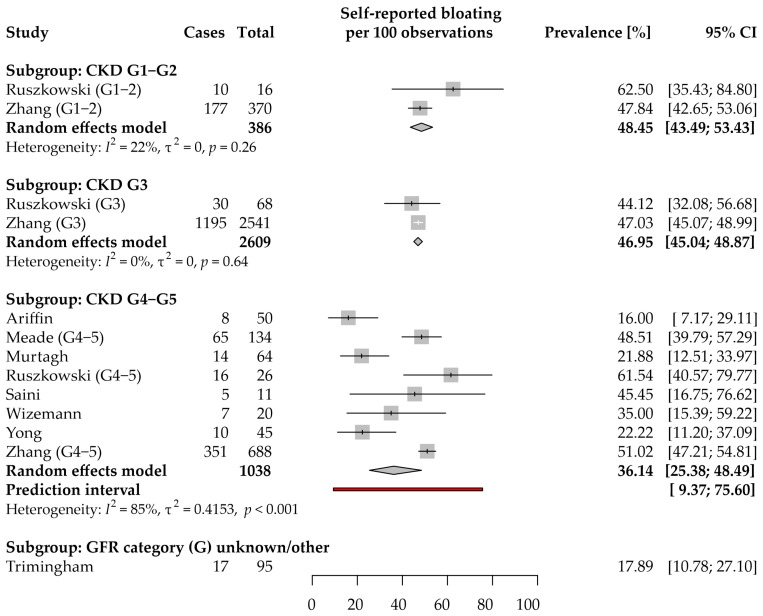
Forest plot with pooled point prevalence estimates for self-reported bloating in chronic kidney disease by stages.

Data on the self-reported bloating prevalence in CKD were extracted from 9 studies [9,12,30,32,42,44,47,56,62] that enrolled 4128 participants (Figure 5). All but one study were included in the meta-analyses. Four studies each were from the WHO European [9,30,44,62] and Western Pacific [12,42,47,56] Regions; one study reported data acquired in the Region of the Americas [32]. The included studies were characterized by using different methods to collect data on symptom prevalence: three used face-to-face interview/authors’ questionnaires; two, the MSAS-SF questionnaire [44,62]; each of the other studies used a different questionnaire.

Based on two studies [9,32], the estimated average prevalence in the CKD G1–2 subgroup was 48.45% (95% CI: 43.5–53.4%). There was no significant heterogeneity between the data from both studies (τ^2^ = 0; *I*^2^ = 22%; *p* = 0.26).

The same two studies were the only source of data for the CKD G3 subgroup. The estimated average prevalence was 46.95% (95% CI: 45.0–48.9%). Data from both studies were consistent (τ^2^ = 0; *I*^2^ = 0%; *p* = 0.64).

The CKD G4–5 subgroup analysis included 8 studies [9,30,32,42,44,47,56,62]. The estimated average prevalence was 36.1% (95% CI: 25.4–48.5%). The results of the included studies ranged between 16 and 62%, and the differences can be attributed mainly to the between-study variance (τ^2^ = 0.42; *I*^2^ = 85%). The 95% PI for the prevalence in new studies ranged from 9.4 to 75.6%. Based on two studies that simultaneously reported the prevalence in all CKD stages, no significant differences in the odds of reporting the symptoms were found between CKD G3 patients and either G1–2 (*p* = 0.55) or G4–5 (*p* = 0.21) (Figures S7 and S8).

Table S16 summarizes the data on the severity of abdominal bloating. Only one study reported data for the CKD G1–2 and G3 subgroups [9]. Among 10 CKD G1–2 patients with the symptom, 80.0% (95% CI: 70–100%) experienced mild, and 20.0% (95% CI: 10.0–48.7%) moderate severity of bloating, while severe or overwhelming bloating was reported by 0% (95% CI: 0–34.3%) of patients each. Among 30 CKD G3 patients with abdominal bloating, mild severity was reported by 50.0% (95% CI: 33.3–68.2%), moderate by 46.7% (95% CI: 30.0–64.8%), severe by 3.3% (95% CI: 0–21.5%), and overwhelming by 0% (95% CI: 0–18.2%). Three studies provided results on bloating severity in CKD G4–5, but data could have not been meta-analyzed (Table S16).

Table S17 summarizes the implications of abdominal bloating in CKD patients. Each of the findings was reported in a single study; therefore, the generalizability of the bloating implications may be limited.

Taken together, even though self-reported bloating seems to be one of the most common GI symptoms, its prevalence in the early stages of CKD has rarely been studied. Moreover, no study provided data on the risk factors associated with the increased burden of the symptom in CKD. Since no study used Rome criteria, functional bloating prevalence remains unknown in CKD. 

### 3.6. Abdominal Pain: Prevalence and Severity

**Figure 6:**
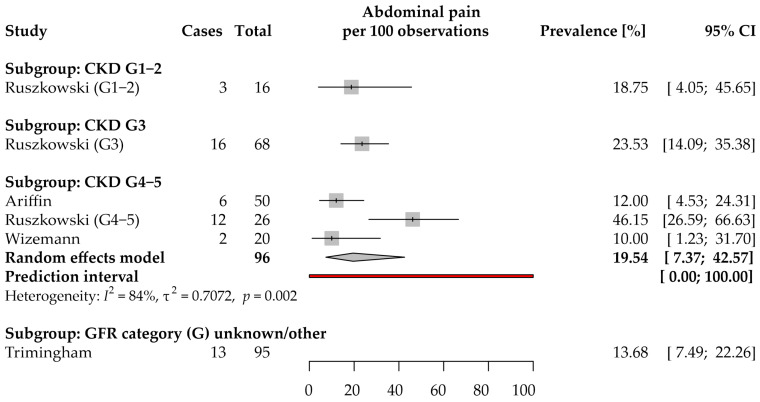
Forest plot with pooled point prevalence estimates for self-reported abdominal pain in chronic kidney disease by stages.

Only four studies provided data on the self-reported abdominal pain prevalence in CKD (Figure 6) [9,12,30,42]. Two studies each were from the WHO European [9,30] and Western Pacific [12,42] Regions. The included studies were characterized by using different methods to collect data on symptom prevalence: two used face-to-face interviews, the others used questionnaires such as the Patient Assessment of Constipation-Symptoms (PAC-SYM) questionnaire, and the “Bowel health questionnaire”. Additionally, one study each presented results on autosomal dominant polycystic kidney disease (ADPKD) [49] and CKD secondary to Fabry disease [25]. We did not identify any study providing data about the prevalence of centrally mediated abdominal pain syndrome in CKD. The prevalence of abdominal pain in CKD secondary to ADPKD or Fabry disease is described in Supplementary Materials, Results S2.

Data on self-reported abdominal pain prevalence among patients with CKD G1–2 (18.8%; 95% CI: 4.1–46.7%) and CKD G3 (23.5%; 95% CI: 14.1–35.4%) were extracted from only one study [9]; therefore, meta-analyses were not performed for these subgroups.

The CKD G4–5 subgroup analysis included 3 studies [9,30,42]. The estimated average prevalence was 19.5% (95% CI: 7.4–42.6%). Ruszkowski et al. [9] observed substantially higher prevalence than in the other two studies; the difference was unlikely from sampling variance (τ^2^ = 0.71; *I*^2^ = 84%). We suppose this is due to the use of paper questionnaires by Ruszkowski et al. and face-to-face interviews by the other two research teams. The prevalence in future studies is totally uncertain (95% PI: 0–100%).

Three studies provided data on the symptom implications (Table S19). Briefly, Ruszkowski et al. analyzed the associations between abdominal pain and both HRQoL and quality of sleep in CKD [9,46]. Miskulin et al. questioned the association between either eGFR or height-adjusted total kidney volume and abdominal pain in ADPKD patients [49].

In conclusion, based on both the limited number and heterogeneous characteristics of the included studies, the results are uncertain. Additionally, there were no data on the symptom prevalence coming from the CKD populations living in the African, American, South-East Asian, and Eastern Mediterranean Regions.

### 3.7. Stool Consistency (Bristol Stool Scale)

The stool appearance and consistency were classified with the Bristol Stool Form Scale (BSFS) into seven stool types. Types 1–2 represent abnormally hard stools (and in conjunction with other symptoms indicative of FC or the constipation-predominant subtype of irritable bowel syndrome), while types 6–7 represent abnormally liquid stools.

Six studies provided data on stool consistency in CKD patients: five studies reported prevalence of both types 1–2 and 6–7 stool forms among 482 patients [9,12,33,40,47], while one study provided data on types 1–2 only in 21 patients [55]. All studies were conducted or published after 2015, and came from Australia (3 studies: [12,47,55]), Europe (2 studies: [9,33]), and Brazil [40]. Table 2 shows the extracted and pooled prevalence of stool types 1–2, 3–5, and 6–7.

Two studies, both conducted in Europe, were included in the prevalence analysis for the CKD G1–2 subgroup [9,33]. The estimated average prevalence of types 1–2 was 14.8% (95% CI: 0.8–37.5%), whereas for types 6–7, it was 8.0% (95% CI: 0–24.2%). While the prevalence of types 3–5 was similar in both studies (76–77%), the prevalence of having either abnormally hard, or loose, stool was quite different in these studies (*I*^2^ = 61%).

Three studies were included in the prevalence analysis for the CKD G3 subgroup [9,33,40]. The estimated average prevalence of types 1–2 was 23.0% (95% CI: 10.9–37.3%), whereas for types 6–7, it was 13.2% (95% CI: 4.1–25.5%). Nearly a half of the total variance can be attributed to sampling errors (*I*^2^ = 54%).

Four studies were included in the prevalence analysis for the CKD G4–5 subgroup [9,33,40,47]. The estimated average prevalence of types 1–2 was 24.5% (95% CI: 13.2–37.8%), whereas for types 6–7, it was 15.7% (95% CI: 6.5–27.6%). There was substantial between-studies heterogeneity (τ = 0.058, *I*^2^ = 72%); however, the elimination of results from [47] would reduce the estimated *I*^2^ to 0% and lead to higher estimates for the prevalence of both hard stools (30.8%) and loose stools (17.0%).

The relationships between stool consistency and other data are shown in Table S20. Briefly, two research teams found that the harder the stool consistency, the higher the concentration of some uremic solutes such as *p*-cresyl sulfate and hippuric acid in serum/plasma [33,40,48]. Both Meade et al. and Ramos et al. reported no associations between dietary parameters and stool consistency [40,47]. Ruszkowski et al. found that taking diuretics was independently associated with an increased prevalence of reporting type 1–2 (i.e., hard) stool form [9]. Interestingly, implications for HRQoL were explored in one study only: no associations between types 1–2 form and either HRQoL or sleep quality were found [9,46].

To sum up, the results of a small number of studies suggest that about two-fifths of patients with advanced CKD may have abnormal stool consistency. Unlike self-reported constipation and diarrhea, stool form appears to be less related to lower HRQoL and more related to increased concentrations of uremic toxins, or at least those formed in the lumen of the large intestine. Further studies, especially outside of Europe and Australia, are needed to assess the impact of CKD progression and frequently used drugs on stool consistency (e.g., via alternation of whole gut transit time). With the help of multi-omics data, it will be possible to identify more uremic solutes associated with hard stool consistency.

### 3.8. Number of Bowel Movements per Week

Data on the number of bowel movements per week were extracted from three studies [9,12,47] enrolling 339 CKD patients, and one study [45] enrolling 2245 DKD patients. Three studies were conducted in the Western Pacific Region (Australia [12,47] and Japan [45]), and one in Europe (Poland [9]). The authors used their own questionnaires to collect data on the outcome.

Data on the weekly frequency of defecation among patients with CKD G1–2 and CKD G3 were extracted from one study only [9]; therefore, meta-analyses were not performed for these subgroups. Estimated prevalence and its 95% CI based on this study for both subgroups are presented in Table S21. We were able to conduct a meta-analysis for the CKD G4–5 subgroup with data from two studies [9,47]: commonly, CKD G4–5 patients had a BM once daily [40.5% (95% CI: 9.4–72.4%)], whereas 31.9% (95% CI: 4.4–64.4%), 26.1% (95% CI: 1.8–58.5%), and 1.5% (95% CI: 0–14.7%) CKD G4–5 patients had more than 7, less than 7 but at least 3, and less than 3 BMs per week, respectively (Table S21).

The identified relationships between the number of BMs per week and HRQoL, clinical data, or laboratory test results are presented in Table S22. Interestingly, the frequency lower than 7 BMs/week (i.e., less than once a day) was associated with impaired HRQoL in CKD, and an increased risk of nephropathy in patients with diabetes. However, since each of the implications was reported in a single study, the generalizability of the implications is limited.

To conclude, the described implications of having a bowel movement less than once a day seem worthy of more extensive research, especially since information on BM frequency is easy to obtain.

### 3.9. Fecal Incontinence and Rectal Pain

Two outcomes, the prevalence of both fecal incontinence and rectal pain in non-dialysis CKD patients, were reported in one study only [47]. It came from Australia, and the symptoms were assessed using a modified 28-item Gastrointestinal Symptom Rating Scale. Fecal incontinence was reported by 46 [34.3% (95% CI: 26.4–43.0%)] and rectal pain by 18 [13.4% (95% CI: 8.2–20.4%)] out of 134 CKD G4–5 patients. The prevalence of both symptoms in earlier CKD stages, as well as in another geographical localization, remain unknown. 

The authors of the mentioned study stated that in the combined group of non-dialysis and dialysis CKD patients, “there was no significant association of fruit, vegetables, wholegrains or legumes intake with any GI symptom” (*p* not reported) [47].

In light of the sparse data presented above, patients with CKD G4–5 may have a surprisingly high prevalence of fecal incontinence; however, without further research the actual prevalence, especially outside Australia, remains unknown.

### 3.10. Sensitivity Analyses

The estimated pooled prevalence of the symptoms remained stable in sensitivity analyses, and the differences between the reference and other models were, in more than four-fifths of the cases, lower than 1%. We listed all estimates with a difference greater than one percent in Table S23.

## 4. Discussion

Our study aimed to comprehensively determine the prevalence, severity, and implications of lower GI symptoms in non-dialysis CKD patients. This population required special interest because non-dialyzed patients constitute more than 99% of all CKD patients, whereas, when compared to other frequent conditions, the overall burden of CKD is very high [1]. By focusing on a single symptom group, and using a widespread search strategy (including citation chasing of symptoms questionnaires), we uncovered a significant number of papers previously overlooked in the context of assessing CKD symptom prevalence. Lower GI symptoms, particularly abdominal bloating and self-reported constipation, are common in non-dialysis CKD patients, but their severity is limited. The literature showed the multiple relationships between lower GI symptoms and HRQoL, clinical outcomes, and laboratory data [9,23,35,39,40,46,49,50,51,52,53]. Our work is the first to analyze in detail the knowledge of lower GI symptomatology in non-dialysis-dependent CKD; thus, most likely for the first time, we demonstrated some novel findings that could not be concluded with sufficient certainty from the original studies (e.g., increased odds of self-reported constipation in CKD patients with more advanced disease). The meta-analysis of results from multiple studies makes the conclusions more convincing and verifies previous smaller observations with higher statistical power.

In an excellent narrative review by Sumida et al. fully dedicated to constipation in CKD [5], the part about constipation epidemiology was based mainly on the information about symptom prevalence in dialysis patients (systematically reviewed earlier [10]). Sumida et al. highlighted a scarcity of information on the prevalence of constipation among non-dialysis-dependent CKD patients. We believe that our work, with several meta-analyses on such specific outcomes as the prevalence of self-reported constipation, FC, or hard stool consistency, at least partially fills the knowledge gap identified by Sumida et al. and provides details on lower GI symptoms experienced in CKD. Our findings can be used to update the Kidney Disease: Improving Global Outcomes (KDIGO) report on Supportive Care in CKD [70]. In this report, the prevalence of “constipation” and “diarrhea” was based on 17 and 10 studies, respectively. Not only did we include more studies and specify the meaning of the symptoms, but we also properly analyzed the outcomes in subgroups according to the deterioration of kidney function. More importantly, the report’s authors stated that the “Impact (of either constipation or diarrhea on HRQoL, morbidity, etc.) has not been assessed systematically in CKD”. Our work covers the knowledge gap and illuminates the importance of lower GI symptoms in patients’ experience of CKD.

Since the included studies rarely tested whether the prevalence of GI symptoms differed between CKD patients and the general population [42,66], only a less certain indirect comparison can be made using published data on the prevalence of the symptoms in the general population. It seems that, in comparison to the general population, self-reported abdominal bloating and both functional and self-reported constipation are more common in CKD non-dialysis patients (details in Appendix A). Interestingly, non-dialysis patients have similar odds of reporting abdominal bloating and constipation but might have lower odds of reporting diarrhea when compared with dialysis patients (details in Appendix B).

### 4.1. Limitations of the Evidence Included and the Review Processes Used

Below, we discuss limitations of the included evidence, such as a lack of studies reporting several outcomes, a scarcity of studies from some world regions, and the risk of bias in the included studies.

We did not identify any study providing data about the prevalence of functional diarrhea, functional abdominal bloating/distension, centrally mediated abdominal pain syndrome, or functional anorectal pain in CKD. Given the fact that all functional GI disorders are associated with lower HRQoL in the general population [8], covering this knowledge gap is relevant. 

The meta-analysis methodology provides the possibility to estimate average prevalence across studies. We cannot, however, make informed statements about the prevalence among populations that were not covered by primary studies. That is, the paucity of studies from the African and the Eastern Mediterranean Regions means that knowledge of symptom prevalence among CKD patients living there is limited. Moreover, the extrapolation of the available results to unexamined populations (by continent or ethnicity) may lead to improper conclusions, as significant variations between populations have been described in such aspects as normal bowel movement frequency, and both the prevalence, and core symptoms, of functional GI disorders [8,71,72,73].

Furthermore, the design of the majority of the included studies could introduce a risk of bias. Participants were recruited using convenience sampling rather than recommended methods such as random sampling or inclusion of all patients from a census. It may have led to the selection of nonrepresentative groups of CKD patients (e.g., wanting to express their GI problems in surveys). Such a risk of bias could have been exacerbated further because of the too-narrow inclusion/too-wide exclusion criteria used in a part of the included studies.

Finally, we extracted “implications” in a similar way as in the KDIGO Report on Supportive Care in CKD [70]. That is, we collected data on all reported associations between GI symptoms and HRQoL/clinical data/laboratory test data. Unfortunately, the vast majority of the included studies had a cross-sectional design and did not apply more or less sophisticated methods of causal inference; therefore, the reported associations should not be treated as causal relationships.

We put in a lot of effort to use the most up-to-date and most appropriate methods to meta-analyze the collected data. Nonetheless, we found some limitations in the available methods. Firstly, there are no well-established one-step methods to meta-analyze multinomial data such as symptom severity, stool consistency, and frequency of BMs. We employed a commonly-used multi-stage technique that is implemented in MetaXL and is based on the Freeman–Tukey double arcsine transformation of proportions to stabilize their variances [22]. Even though MetaXL uses the DerSimonian and Laird method to estimate between-study variance, there is a paucity of simulation research that would confirm that it is the optimal approach (it was, however, shown to be a suboptimal method in other conditions [74]). Another alternative that recently emerged is a Bayesian approach that works under the assumption that each category prevalence understood as the probability is distributed as a Dirichlet distribution with a gamma hyperprior [75]. We also found that there is a lack of a validated method to plot a multinomial data meta-analysis. Therefore, we will plan to design several visualizations and test their readability in both experienced and not-experienced users of systematic reviews. In this review, we provided tabulated data, and showed the results in plain text. Because the number of studies included in our meta-analyses was limited, the estimations of both the between-study variance (τ) and *I*^2^ statistic could be imprecise or even biased [76]. We believe that our work will encourage the next research teams to examine the prevalence and severity of GI symptoms in CKD patients, and that we will be able to include many more studies in the update of our work in the next 3–4 years. With new studies, all estimations will be more precise.

### 4.2. Implications of the Results for Practice and Future Research

Below, we discuss how our findings may be applied directly to improve the quality of patient care and to enhance the design and objectives of future research.

Numerous questionnaires designed to assess symptoms in CKD patients do not include questions about the lower GI symptoms (e.g., Symptoms and Problems Subscale from the Kidney Disease Quality of Life 36-item Short Form Survey (KDQOL-36™)). Since patients with GI symptoms may incorrectly evaluate their GI health and rarely discuss their symptoms with a clinician, it is of great importance to actively ask patients about GI symptoms [11,12,77]. Therefore, in agreement with van der Willik et al. [78], we recommend the DSI and the POS-S Renal (and its expanded version: the IPOS-Renal) for routine symptom assessment in CKD patients. Additionally, specific GI questionnaires may be useful for research purposes. Given the especially high prevalence of abdominal bloating and self-reported constipation in CKD, future studies should more frequently use such questionnaires as the Intestinal Gas Questionnaire [79,80] and the PAC-SYM [81] questionnaire. In addition, because of the paucity of data, there is a need to assess the prevalence, risk factors, and implications of functional disorders in CKD; the optimal way is to use the Rome IV Diagnostic Questionnaire for Functional Gastrointestinal Disorders in Adults. The use of specialized questionnaires will improve understanding of patients’ GI symptoms and, when combined with multi-omics data, may reveal the role of GI symptoms in the gut–kidney axis in CKD [82].

According to the evidence-based research approach, not only did we provide pooled prevalence estimates, but we also identified many evidence gaps. Our study may, therefore, serve to calculate sample sizes and choose relevant outcomes in future studies [83]. Furthermore, to gain greater insight into the associations found in cross-sectional studies (e.g., self-reported constipation with HRQoL), there is a need for more high-quality longitudinal studies that will apply causal inference methods [84]. As the pathophysiology of GI symptoms in CKD remains poorly understood, we suggest a wide exploration of both traditional risk factors (including gender, physical activity, and fiber intake [85]) and CKD-related disorders/processes that can contribute to symptoms (e.g., dysregulation of the autonomic nervous system, endocrine disorders, bile acids composition abnormalities, or specific drug side-effects). Finally, the treatment burden inherently associated with CKD (e.g., difficulties associated with taking medicine, problems resulting from fluid restriction, psychological barriers to accessing public toilets in hospital) should be taken into account [86].

The high prevalence of lower GI symptoms should be taken into account in studies focusing on the alteration of GI microbiota in CKD patients. Previous studies have linked several lower GI symptoms with dysbiosis in the general population [87,88], so how it is related to the dysbiosis observed in CKD must be revealed [89]. Surprisingly, none of the studies to date have included patient GI symptomatology in the assessment of dysbiosis in CKD, with one exception that found that variation of the microbial composition was correlated with stool consistency [48].

Finally, interventional studies should be conducted to assess whether symptomatic treatment enhances HRQoL in the case of GI symptoms that were shown to be associated with a worse HRQoL.

As the pace of research on CKD symptomatology increases, we plan to update this systematic review in approximately 3–4 years. This will include broader search methods to include areas not fully covered in this review and will take advantage of emerging methods for meta-analyses. We anticipate the results of a study currently occurring in Uganda [90], and also one in Australia [91], and a full-text article detailing data for non-dialysis CKD patients from a study conducted in Malaysia [92]. Moreover, we will update the meta-analytical methods to provide results that are as reliable as possible.

## 5. Conclusions

The collected data demonstrated that lower GI symptoms, especially abdominal bloating and self-reported constipation, are common in non-dialysis CKD patients, but the severity of the majority of GI symptoms is limited. The lower GI symptoms in CKD warrant interest not only due to their associations with lower HRQoL, but also because of their probable implications indicated in individual reports. Our findings can be used in clinical practice to improve the recognition of GI symptoms in CKD (medical practitioners should use symptom questionnaires that incorporate the most prevalent GI symptoms), as well as in future research to explore knowledge gaps that were identified. To elucidate the pathogenesis of lower GI symptoms in CKD, longitudinal studies using specialized questionnaires and collecting multi-omics data are needed. Because of the variability in GI symptoms’ prevalence worldwide, more data have to be collected, especially in the African and Eastern Mediterranean Regions. All meta-analysis results can be incorporated into the sample size estimation of future studies. Given the widespread GI symptoms in CKD, they should be taken into account in studies evaluating the GI microbiome in CKD.

## Figures and Tables

**Table 2:** Stool consistency in chronic kidney disease according to Bristol Stool Form Scale.

Authors, Reference	Total (N Analyzed)	Bristol Stool Form Scale		
		Type 1–2	Type 3–5	Type 6–7
**CKD G1–2**				
Gryp, T.; et al. [33]	37	4	28	5
Ruszkowski, J.; et al. [9,46]	13	3	10	0
Prevalence (95% CI) ^a^		14.8% (0.8–37.5)	77.2% (51.8–94.9)	8.0% (0–24.2)
**CKD G3**				
Gryp, T.; et al. [33]	44	7	27	10
Ramos, C.I.; et al. [40]	6	1	5	0
Ruszkowski, J.; et al. [9,46]	62	19	37	6
Prevalence (95% CI) ^b^		23.0% (10.9–37.3)	63.8% (47.5–77.6)	13.2% (4.1–25.5)
**CKD G4–5**				
Gryp, T.; et al. [33]	33	9	18	6
Meade, A.; et al. [47]	134	18	98	18
Ramos, C.I.; et al. [40]	36	13	18	5
Ruszkowski, J.; et al. [9,46]	22	6	12	4
Lee, A.; et al. [55]	21	4	17	
Prevalence (95% CI) ^c^		24.5% (13.2–37.8)	59.8% (45.3–73.2)	15.7% (6.5–27.6)
**CKD: eGFR unknown**				
Trimingham, C.; et al. [12]	95	9	77	9
Prevalence (95% CI) ^d^		9.5% (3.2–17.6)	81.1% (74.7–89.1)	9.5% (3.2–17.6)

^a^ Meta-analysis was conducted using a random-effects model with Freeman–Tukey double arcsine transformation of proportions (τ^2^ = 0.079; *I*^2^ = 61%). ^b^ Meta-analysis was conducted using a random-effects model with Freeman–Tukey double arcsine transformation of proportions (τ^2^ = 0.038; *I*^2^ = 54%). ^c^ Meta-analysis of 4 studies was conducted using a random-effects model with Freeman–Tukey double arcsine transformation of proportions (τ^2^ = 0.058; *I*^2^ = 72%); data provided by Lee A were not included due to an incompatible format. ^d^ Confidence intervals for multinomial proportions were calculated according to the method of Sison and Glaz.

## Data Availability

Data are contained within the article and Supplementary Materials.

## Supplementary Materials

The following supporting information can be downloaded at: https://www.mdpi.com/article/10.3390/jcm11216363/s1, Figure S1: Funnel and Doi plot for self-reported constipation in CKD G4–5; Figure S2: Forest plot with pooled odds ratio for self-reported constipation in chronic kidney disease (G4–5 vs. G3); Figure S3: Bubble plot based on meta-regression model: odds ratio for self-reported constipation in chronic kidney disease (G4–5 vs. G3); Figure S4: Funnel and Doi plot for self-reported diarrhea in CKD G4–5; Figure S5: Forest plot with pooled odds ratio for self-reported diarrhea in chronic kidney disease (G3 vs. G1–2); Figure S6: Forest plot with pooled odds ratio for self-reported diarrhea in chronic kidney disease (G4–5 vs. G3); Figure S7: Forest plot with pooled odds ratio for self-reported abdominal bloating in chronic kidney disease (G3 vs. G1–2); Figure S8: Forest plot with pooled odds ratio for self-reported abdominal bloating in chronic kidney disease (G4–5 vs. G3); Table S1: Deviations from the registered protocol with justification; Table S2: Search strategy; Table S3: Symptom questionnaires used for forward citation chasing; Table S4: Items of electronic extraction form; Table S5: Excluded studies with reason; Table S6: Exclusion criteria in the included studies; Table S7: Risk of bias in included studies; Table S8: Severity of self-reported constipation; Table S9: Subgroup analysis for self-reported constipation prevalence in chronic kidney disease (CKD) G4–5; Table S10: Severity of self-reported constipation (alternative version); Table S11: Relationships between self-reported constipation and health-related quality of life (HRQoL), clinical data, or laboratory tests results; Table S12: Relationships between functional constipation and HRQoL, clinical data, or laboratory tests results; Table S13: Severity of self-reported diarrhea; Table S14: Subgroup analysis for self-reported diarrhea prevalence in CKD G4–5; Table S15: Relationships between self-reported diarrhea and HRQoL, clinical data, or laboratory test results; Table S16: Severity of abdominal bloating; Table S17: Relationships between self-reported abdominal bloating and HRQoL, clinical data, or laboratory test results; Table S18: Abdominal pain prevalence and severity in autosomal dominant polycystic kidney disease; Table S19: Relationships between self-reported abdominal pain and HRQoL, clinical data, or laboratory tests results; Table S20: Relationships between stool consistency and HRQoL, clinical data, or laboratory tests results; Table S21: Number of bowel movements per week in patients with CKD or diabetic kidney disease; Table S22: Relationships between the frequency of defecations and HRQoL, clinical data, or laboratory tests results; Table S23: Sensitivity analysis: differences from the reference model exceeding one percent; Supplementary Methods, S1: Eligibility Criteria, S2: Reporting bias assessment, S3: Confidence interval calculation, subgroup analysis, sensitivity analysis, and certainty assessment; Supplementary Results, S1: Study characteristics, S2: Abdominal pain in ADPKD and Fabry disease. References [9,12,18,20,23,24,25,26,27,28,29,30,31,32,33,34,35,36,37,38,39,40,41,42,43,44,45,46,47,48,49,50,51,52,53,54,55,56,57,58,59,60,61,62,63,64,65,66,67,68,69,93,94,95,96,97,98,99,100,101,102,103] are mentioned in Supplementary Methods.

## Appendix A

Only two studies directly compared the prevalence of GI symptoms between CKD patients and the general population [42,66]. The analyses were, however, underpowered to find significant differences between groups (with one exception: Taira et al. found a higher prevalence of constipation among 15 CKD patients than in 76 healthy individuals [13% vs. 1%, *p* = 0.02]) [66].

Our study showed that the prevalence of self-reported constipation in CKD was 31.8% (95% CI: 13.9–54.9%), 29.8% (95% CI: 21.2–40.1%), and 38.8% (95% CI: 30.9–47.4%), in G1–2, G3, and G4–5 CKD stages, respectively. The median prevalence of self-reported constipation in the general population across 22 studies identified in five systematic reviews was 15.1% (IQR: 6.3–24.3%) [104,105,106,107,108,109]. Therefore, in comparison to the general population, self-reported constipation prevalence may be higher in CKD patients even in the early stages of the disease (similar in G1–2 and G3), and further increase with the loss of kidney function (G4–5). In a systematic review and meta-analysis conducted by Barberio et al. the prevalence of functional constipation (according to Rome III criteria, which were used in all studies included in our review) in the general population was 10.4% (95% CI: 6.5–14.9%; I^2^ = 99.8%) [98]. Therefore, in comparison to the general population, the FC prevalence is comparable in patients with early stages of CKD (G1–2: 12% [1.6–38.4%]), but may be increased in patients with CKD G3 (17.3% [10.3–27.6%]), and the difference may be bigger in stages G4–5 (22.0% [8.8–45.1%]).

Self-reported abdominal bloating is reported to be one of the most prevalent GI symptoms in the general population; it was also the most prevalent symptom in our analysis. We found that its point prevalence in CKD was 48.45% (95% CI: 43.5–53.4%; 2 studies), 46.95% (95% CI: 45.0–48.9%; 2 studies), and 36.1% (95% CI: 25.4–48.5%; 8 studies), in G1–2, G3, and G4–5 stages, respectively. Unfortunately, the prevalence of self-reported bloating in the general population was not a focus of any systematic review up to date. The median prevalence of self-reported abdominal bloating in the general population across 9 studies was 20.6% (IQR: 13.4–31.4%). Therefore, in comparison to the general population, self-reported abdominal bloating prevalence seems to be higher in CKD patients, particularly in the early stages of the disease (similar in G1–2 and G3).

## Appendix B

As stated in Table S10, nine studies directly compared the prevalence of self-reported constipation between non-dialysis patients with advanced stages of CKD and dialysis-dependent patients; the results were highly inconsistent. There were studies showing both lower (significantly: [31]; non-significantly: [39,55,56,57,58]) and higher (significantly: [68]; non-significantly: [24,42]) odds of self-reported constipation in dialysis patients than in patients with CKD G4–5. The median prevalence of self-reported constipation in dialysis patients across 13 studies identified in the systematic reviews conducted by Zuvela et al. [10] and our work was 30.1% (IQR: 25.7–43.2%), which is quite similar to the prevalence in CKD (particularly, the early stages). To sum up, there is no evidence supporting the hypothesis that dialysis patients have a significantly different prevalence of self-reported constipation than non-dialysis patients with CKD G4–5.

In the case of self-reported diarrhea (Table S14), there were eight studies that directly compared its prevalence between CKD non-dialysis and dialysis-dependent patients. In the majority of studies, non-dialysis patients had lower odds of reporting diarrhea (significantly in four studies: [24,36,39,68]; non-significantly in three studies: [31,42,57]) than patients treated with dialysis; only one study showed another trend [58]. The median prevalence of self-reported diarrhea in dialysis patients across 12 studies identified in the systematic reviews conducted by Zuvela et al. and our work was 16.9% (IQR 9.7–28.4) [10]; that is close to the average prevalence in G4–5 in our meta-analysis. To sum up, non-dialysis patients with advanced stages of CKD may have a lower or comparable prevalence of self-reported diarrhea than patients requiring dialysis.

As stated in Table S16, only two studies directly compared the prevalence of self-reported bloating between CKD non-dialysis and dialysis-dependent patients: both observed a slightly higher prevalence in dialysis patients but found no significant differences between these two groups [42,56]. The median prevalence of self-reported bloating in dialysis patients across 5 studies identified in the systematic reviews conducted by Zuvela et al. and our work was 27.6% (IQR 21.5–41.7%) [10], which is within the confidence interval of the average prevalence in CKD G4–5. Taken together, there are not enough data to reliably compare abdominal bloating prevalence between non-dialysis patients with advanced stages of CKD and dialysis-dependent CKD patients.
