# Supplementary material for: Prevalence and Severity of Lower Gastrointestinal Symptoms amongst Non-Dialysis Chronic Kidney Disease Patients: A Systematic Review and Meta-Analysis
Source: J Clin Med. 2022 Oct 28;11(21):6363. doi: 10.3390/jcm11216363 (PMC9656905; doi:10.3390/jcm11216363)
Supplement: Supplementary file 1 [file jcm-11-06363-s001.zip › jcm-1950103-supplementary.pdf]

Supplementary Material to:

Prevalence and Severity of Lower Gastrointestinal Symptoms amongst Non-Dialysis Chronic Kidney Disease Patients: A Systematic Review and Meta-Analysis

Jakub Ruszkowski, Katarzyna Majkutewicz, Zbigniew Heleniak, Jacek M. Witkowski and Alicja Dębska-Ślizień

Supplementary Contents

Supplementary Methods 3

S1. Eligibility Criteria 3

S2. Reporting bias assessment 4

S3. Confidence interval calculation, subgroup analysis, sensitivity analysis, and certainty assessment 4

Supplementary Results 5

S1. Study characteristics 5

S2. Abdominal pain in ADPKD and Fabry disease 6

Table S1. Deviations from the registered protocol with justification 7

Table S2. Search strategy 8

Table S3. Symptom questionnaires used for forward citation chasing 13

Table S4. Items of electronic extraction form 13

Table S5. Excluded studies with reason 14

Table S6. Exclusion criteria in the included studies. 29

Table S7. Risk of bias in included studies. 31

Table S8. Severity of self-reported constipation 35

Table S9. Subgroup analysis for self-reported constipation prevalence in chronic kidney disease (CKD) G4-5 36

Table S10. Severity of self-reported constipation (alternative version) 37

Table S11. Relationships between self-reported constipation and health-related quality of life (HRQoL), clinical data, or laboratory tests results 37

Table S12. Relationships between functional constipation and HRQoL, clinical data, or laboratory tests results. 39

Table S13. Severity of self-reported diarrhea 40

Table S14. Subgroup analysis for self-reported diarrhea prevalence in CKD G4-5 41

Table S15. Relationships between self-reported diarrhea and HRQoL, clinical data, or laboratory test results 42

Table S16. Severity of abdominal bloating 44

Table S17. Relationships between self-reported abdominal bloating and HRQoL, clinical data, or laboratory test results. 45

Table S18. Abdominal pain prevalence and severity in autosomal dominant polycystic kidney disease. 46

Table S19. Relationships between self-reported abdominal pain and HRQoL, clinical data, or laboratory tests results. 47

Table S20. Relationships between stool consistency and HRQoL, clinical data, or laboratory tests results. 48

Table S21. Number of bowel movements per week in patients with CKD or diabetic kidney disease. 49

Table S22. Relationships between the frequency of defecations and HRQoL, clinical data, or laboratory tests results. 50

Table S23. Sensitivity analysis: differences from the reference model exceeding one percent. 51
Supplementary Figures 52
Figure S1. Funnel and Doi plot for self-reported constipation in chronic kidney disease (CKD) G4–5 52
Figure S2. Forest plot with pooled odds ratio for self-reported constipation in CKD (G4–5 vs G3) 52
Figure S3. Bubble plot based on meta-regression model: odds ratio for self-reported constipation in CKD (G4–5 vs G3) 53
Figure S4. Funnel and Doi plot for self-reported diarrhea in CKD G4–5 53
Figure S5. Forest plot with pooled odds ratio for self-reported diarrhea in CKD (G3 vs G1-2) 53
Figure S6. Forest plot with pooled odds ratio for self-reported diarrhea in CKD (G4-5 vs G3) 54
Figure S7. Forest plot with pooled odds ratio for self-reported abdominal bloating in CKD (G3 vs G1–2) 54
Figure S8. Forest plot with pooled odds ratio for self-reported abdominal bloating in CKD (G4–5 vs G3) 54
Supplementary References 56

Supplementary Methods

S1. Eligibility Criteria

Population and settings

Inclusion: Adults (age ≥ 18 years) with CKD that are treated without dialysis.

Exclusion: People under 18 years of age; pregnant women; treatment with dialysis; inpatients (being at an inpatient facility for more than one day)

Since we were interested in the symptomatic profile of the CKD population in community-based settings, only outpatients receiving non-acute care or non-receiving any treatment were included. We restricted the scope of our interest to the patients not receiving dialysis therapy because renal replacement therapies are linked with several additional factors that may contribute to symptomatology. Studies in any geographic location were included.

Outcome

Primary outcomes were self-reported symptoms as follows: 1-2. Prevalence and severity of abdominal pain. 3-4. Prevalence and severity of abdominal bloating. 5-6. Prevalence and severity of constipation. 7-8. Prevalence and severity of diarrhea. 9-10. Prevalence and severity of fecal incontinence. 11-12. Prevalence and severity of rectal pain.

To be included, the study could not have reported only a composite outcome that shared a name with the outcome of interest. Examples of such composite outcomes included scales from the Gastrointestinal Symptom Rating Scale (GSRS; e.g. "abdominal pain" scale is the sum of items assessing (1) pain/discomfort in the upper abdomen, (2) hunger pains in the stomach, and (3) nausea). The only exceptions were functional GI disorders diagnosed according to the Rome criteria (see below). We did not include studies using proxy indicators to assess symptom prevalence, i.e. laxative use to assess constipation prevalence.

Secondary outcomes were as follows: 1. Prevalence of functional constipation. 2. Prevalence of each of the three stool consistency types. 3. Frequency of bowel movements (BMs). 4. Prevalence of functional diarrhea. 5. Prevalence of functional abdominal bloating/distension. 6. Prevalence of centrally mediated abdominal pain syndrome. 7. Prevalence of functional anorectal pain. 8. Associations/correlations between each of the lower GI symptoms/syndromes and health-related quality of life. 9. Associations/correlations between each of the lower GI symptoms/syndromes and

lab results. 10. Associations/correlations between each of the lower GI symptoms/syndromes and clinical data.

Stool consistency was classified as too hard (Bristol stool scale: types 1-2), normal (Bristol stool scale: types 3-5), or too loose (Bristol stool scale: types 6-7). Frequency of BMs was analyzed as a 4-level categorical variable: (1) less than three BMs per week, (2) at least three but less than seven BMs per week, (3) seven BMs per week (= once a day), (4) more than seven BMs per week (= more than once a day).

Design

We included observational studies, including case-control, cross-sectional, and cohort studies. Biographies, randomized controlled trials, reviews, meta-analyses, case reports, editorials, and studies protocols were excluded. Cross-sectional analysis of the baseline data from randomized controlled trials was permitted.

S2. Reporting bias assessment

To assess "reporting biases" such as selective non-publication (publication bias) and selective non-reporting of results, we conducted a Peters' regression test, calculated the Luis Furuya-Kanamori (LFK) index and generated both funnel and Doi plots for meta-analyses including at least 10 studies [1,2]. If asymmetry was detected, we reported whether it may have resulted from positive reporting bias (LFK index > 1; favoring studies that reported higher prevalence) or a negative one (LFK index < −1; favoring studies that reported lower prevalence). Calculations and plotting were done using functions from 'meta' (*metabias*, *funnel*) and 'metasens' (*lfkindex*, *doiplot*) R packages [3,4]. Since, as expected, included observational studies were rarely preceded by a published protocol, we did not plan nor perform comparison of planned and reported outcomes. Instead, in the case of data lacking, we tried to contact authors to obtain missing data.

S3. Confidence interval calculation, subgroup analysis, sensitivity analysis, and certainty assessment

Confidence intervals (95% CI) for individual study results of single proportions were estimated using the conservative exact Clopper–Pearson method. If data for multinomial proportions (e.g. symptom severity classified to at least 3 categories) were available from one study only, we estimated 95% CI with the Sison & Glaz method using *MultinomCI* function from 'DescTools' (version 0.99.44) R package [5].

In the case of both significant heterogeneity and at least 10 studies in the meta-analysis, we conducted pre-specified subgroup analyses [according to the date of data collection (before 2000, 2000-2010, after 2010), location of data collection (WHO Regions), age of participants, and sex of participants]. Performing subgroup analysis of meta-analysis with less than 10 studies would result in meaningless results due to too-low statistical power. Formal certainty assessment was not performed because there is no evidence-based tool for assessing studies of the prevalence of disease [6]. However, we followed the GRADE framework and underlined study limitations (RoB), inconsistency of results, imprecision, and reporting bias [7].

We conducted 7 sensitivity analyses for each single proportion (prevalence) outcome using the 'altmeta' package (version 3.3). Using *maprop.glmm* function, we tested how changing a logit transformation into another link [probit, cauchit, and complementary log-log links (cloglog)] would affect pooled prevalence estimation with GLMM [8]. Since a conventional two-step method to meta-analyze single proportion is still popular and the Freeman–Tukey double arcsine transformation (FTT) is the most widely used—yet controversial—to normalize proportion distribution before pooling [9,10], we used a *maprop.twostep* function with two methods of the between-study variance (ML, REML) and two ways of back-transformation [using the harmonic mean of the study-specific sample sizes ("harmonic" in tables) or the inverse of the synthesized result's variance as the overall sample size ("inverse var" in tables)] [8,9].

Supplementary Results

S1. Study characteristics

The vast majority of studies reported data collected for CKD G4-5 subgroup. Information for this subgroup was extracted from 30 studies, and nearly a half of them (14 studies) presented the cause of CKD. Mean/median age in the studies for CKD G4-5 subgroups ranged from 50 up to 83 years (on average: 66.6), while males consisted of 39-77% (on average: 59.6%) participants. Data on symptoms in patients with CKD G3 were extracted from 10 studies [11–20]. Mean/median age in these studies ranged from 56 up to 81 (on average 63.5), and males represented 52-83% (on average 64.4%) participants. We found far fewer studies reporting data on GI symptomatology in the early stages of CKD: data for the CKD G1-2 subgroup was extracted from 6 studies only [11–13,18–20]. Mean age in these studies ranged from 50 up to 66 (on average 55), while males represented 32-100% (on average 62.7%) participants. Cause of CKD was reported only in one study in this subgroup [20].

S2. Abdominal pain in ADPKD and Fabry disease

We suspected identifying more studies assessing the prevalence of abdominal pain in such diseases as ADPKD and Fabry disease. However, we had to exclude a number of identified studies, mainly due to them reporting composite outcomes (e.g. “lumbar and/or abdominal pain” instead of abdominal pain in ADPKD) or not presenting analysis for the non-dialysis CKD subgroup (aggregated data of Fabry disease patients with normal and abnormal kidney function). In a study conducted in Turkey, four out of seven [57.1% (95% CI 18.4 to 90.1%)] male patients with CKD caused by Fabry disease reported abdominal pain. Interestingly, none out of four female patients reported the symptom. We show extracted data on abdominal pain for ADPKD in the Supplementary Table S18.

Table S1. Deviations from the registered protocol with justification

Protocol	Current version	Justification
Additional outcome(s): (…)<br>2. Prevalence of having stool form suggesting constipation (Bristol stool scale: types 1-2).<br>(…)<br>6. Prevalence of having stool form suggesting diarrhea (Bristol stool scale: types 6-7).	Secondary outcomes were as follows: (…) 2. Prevalence of three stool consistency types. (…) Stool consistency was classified as too hard (Bristol stool scale: types 1-2), normal (Bristol stool scale: types 3-5), or too loose (Bristol stool scale: types 6-7).	These 2 outcomes are supposed to be correlated, therefore both would be dependent effects sizes. To obtain unbiased results, we decided to meta-analyze stool consistency as a 3-level categorical variable.
Additional outcome(s): (…)<br>3. Prevalence of having less than 7 bowel movements per week.<br>4. Prevalence of having less than 3 bowel movements per week.	Secondary outcomes were as follows: (…) 3. Frequency of bowel movements (BMs). (…) Frequency of BMs was analyzed as a 4-level categorical variable: (1) less than three BMs per week, (2) at least three but less than seven BMs per week, (3) seven BMs per week (= once a day), (4) more than seven BMs per week (= more than once a day).	These 2 outcomes are supposed to be correlated, therefore both would be dependent effects sizes. To obtain unbiased results, we decided to meta-analyze bowel movement frequency as a 4-level categorical variable.
Sensitivity analysis [*of single proportion outcomes*] will be conducted using a random-effects meta-analysis with the Freeman-Tukey double arcsine transformation of proportions.	We conducted 7 sensitivity analyses for each single proportion (prevalence) outcome using 'altmeta' package (version 3.3). Using maprop.glmm function, we tested how changing a logit transformation into another link (probit, cauchit, and cloglog) would affect pooled prevalence estimation with GLMM. Since a conventional two-step method to meta-analyze single proportion is still popular and the Freeman–Tukey double arcsine transformation (FTT) is the most widely used—yet controversial—to normalize proportion distribution before pooling, we used the maprop.twostep function with two methods of the between-study variance (ML, REML) and two ways of back-transformation (using the harmonic mean of the study-specific sample sizes or the inverse of the synthesized result's variance as the overall sample size).	Justified in the "current version".
[*not mentioned*]	To assess "reporting biases" such as selective non-publication (publication bias) and selective non-reporting of results, we calculated the Luis Furuya-Kanamori (LFK) index and generated both funnel and Doi plots for meta-analyses including at least 5 studies. If asymmetry was detected, we planned to report whether it may have resulted from positive reporting bias (LFK index > 1; favoring studies that reported higher prevalence) or negative one (LFK index < −1; favoring studies that reported lower prevalence). Since, as expected, included observational studies were rarely preceded by a published protocol, we did not plan nor perform comparison of planned and reported outcomes. Instead, in case of data lacking, we tried to contact authors to obtain missing data.	Reporting biases were planned. However, since PROSPERO does not require a description of reporting bias assessment, this description was omitted from the original version of the protocol.
For each of the outcome, the following variables will be extracted from each paper:<br>(…) [outcomes] in groups according to GFR (GFR higher than 60; GFR 30-60; GFR below 30; GFR below 15 mL/min/1.73 m^2) and according to albuminuria/UACR (below 30; 30-300; above 300 mg/24 h or mg/g).<br>(…) Data that used the same (or enough similar) definition of symptom will be synthesized when available from at least 2 studies.	Since the burden of disease- and treatment-related symptoms increases with progression of CKD, we decided to meta-analyze all outcomes separately in the subgroups as follows: early CKD (G1 and G2, i.e. eGFR ≥ 60 mL/min per 1.73 m2), moderate CKD (G3, i.e. eGFR 30–59 mL/min per 1.73 m2), advance CKD (G4 and G5, i.e. eGFR < 30 mL/min per 1.73 m2).	While the separate analyses for each eGFR group were pre-planned and expressed in the "Data extraction (selection and coding)" section, we unintentionally omitted the direct statement in the "Strategy for data synthesis" / "Analysis of subgroups or subsets" sections.
Subgroup analyses by the presence of albuminuria, CKD etiology, date of data collection (before 2000, 2000-2010, after 2010), location of data collection, race/ethnicity, age group will be performed with the test for difference between the groups.	We planned to conduct separate meta-analyses according to albuminuria categories; however, data on albuminuria was not reported in the included studies. (…) We did not conduct pre-specified subgroup analyses to explore possible causes of heterogeneity among study results because of the too-small number of those studied, that would cause a too-low statistical power.	Justified in the "current version".

[*not mentioned*]	To compare the prevalence between subgroups, meta-analysis of within-study odds ratios was conducted using random effects model with restricted maximum-likelihood (REML) estimator for τ^2.	Subgroup effects are more credible if the comparison is made within rather than between studies with different methodological qualities, geographic localization, etc. The analysis was not pre-planned but requested during peer review.

Table S2. Search strategy

Database	Search
**MEDLINE (via PubMed)**	**#1**<br>(chronic kidney[tw] OR chronic renal[tw] OR chronic glomerul*[tw] OR chronic nephro*[tw] OR progressive kidney[tw] OR progressive glomerul*[tw] OR progressive nephro*[tw] OR diabetic kidney[tw] OR diabetic renal[tw] OR diabetic glomerul*[tw] OR ckd[tw] OR esrd[tw] OR<br>((diabet*[tw] OR “Disease Progression”[mh:noexp] OR “Recurrence”[mh:noexp]) AND nephropath*[tw])<br>OR uremi*[tw] OR uraemi*[tw] OR proteinuri*[tw] OR nephrosclerosis[tw] OR glomerulosclerosis[tw] OR glomerular sclerosis[tw] OR “Glomerular Filtration Rate”[majr:noexp] OR microalbuminuri*[tw] OR macroalbuminuri*[tw] OR albuminuri*[tw] OR calciphylaxis[tw] OR secondary hyperparathyroidism[tw] OR “Hyperparathyroidism, Secondary”[mh:noexp] OR tubulointerstitial fibrosis[tw] OR interstitial fibrosis[tw] OR renal fibrosis[tw] OR kidney fibrosis[tw] OR vascular calcification*[tw] OR alport*[tw] OR denys-drash[tw] OR glomerulopathy[tw] OR hypoalbuminemi*[tw] OR hypoalbuminaemi*[tw] OR multicystic kidney*[tw] OR polycystic kidney*[tw] OR cystic kidney*[tw] OR kidney disease*[tw] OR kidney failur*[tw] OR kidney function*[tw] OR kidney insufficienc*[tw] OR kidney disorder*[tw] OR kidney dysfunction[tw] OR renal disease*[tw] OR renal failur*[tw] OR renal function*[tw] OR renal insufficienc*[tw] OR renal disorder*[tw] OR renal dysfunction[tw] OR<br>((kidney[tw] OR renal[tw]) AND<br>(ckf[tw] OR crd[tw] OR crf[tw] OR eskd[tw] OR eskf[tw] OR esrf[tw] OR hyperparathyroidism[tw] OR end-stage[tw] OR endstage[tw] OR eGFR[tiab]))<br>OR<br>((kidney transplant*[tiab] OR renal transplant*[tiab])<br>AND (candidates[tiab] OR wait list*[tiab] OR waiting list*[tiab]))<br>OR<br>((ureteral obstruction[tw] OR nephritis OR glomerulonephritis OR nephrop* OR<br>(obstruct*[tiab] AND (kidney*[tiab] OR renal[tiab] OR nephropathy[tiab])))<br>AND (sclerosi*[tw] OR fibrosi*[tw] OR fibrotic[tw]))<br>)<br><br>**#2**<br>"Abdominal Pain"[MeSH Terms] OR "abdominal pai*"[Title/Abstract] OR "abdomen pai*"[Title/Abstract] OR "pain in abdomen"[Title/Abstract] OR "painful abdom*"[Title/Abstract] OR<br>"Flatulence"[MeSH Terms] OR "flatulen*"[Title/Abstract] OR "bloated"[Title/Abstract] OR "bloating"[Title/Abstract]<br>OR<br>"diarrhea"[MeSH Terms] OR "diarrh*"[Title/Abstract] OR "loose stoo*"[tiab]<br>OR<br>"fecal incontinence"[MeSH Terms] OR "Encopresis"[Mesh] OR "faecal incontinenc*"[Title/Abstract] OR "fecal incontinenc*"[Title/Abstract] OR "bowel incontinenc*"[Title/Abstract] OR<br>"constipation"[MeSH Terms] OR "constipat*"[Title/Abstract] OR "obstipation"[Title/Abstract]<br>OR<br>"rectal pain"[Title/Abstract] OR "anorectal pain"[Title/Abstract] OR “functional anorectal”[tiab]<br>OR

	"defecation"[MeSH Terms] OR "defaecat*"[Title/Abstract] OR "defecat*"[Title/Abstract] OR "bowel movemen*"[All Fields]<br>OR<br>"Bristol Stool Form"[tiab] OR "Bristol Stool Scale"[tiab] OR BSFS[tiab]<br>OR “gastrointestinal symptom*”[tiab]<br><br>**#3**<br>#1 AND #2 NOT ("animals"[mesh] NOT "humans"[mesh])<br>NOT ("animals"[mesh] NOT "humans"[mesh])<br>NOT (("infant"[MeSH Terms] OR "child"[MeSH Terms] OR "adolescent"[MeSH Terms]) NOT "adult"[MeSH Terms])<br>NOT ("Review"[Publication Type] OR "systematic review"[Publication Type] OR "Network Meta-Analysis"[MeSH Major Topic] OR "case reports"[Publication Type] OR "editorial"[Publication Type] OR "biography"[Publication Type] OR "Randomized Controlled Trial"[Publication Type] OR "Clinical Trial Protocol"[Publication Type])<br>NOT ("A Case Series"[Title] OR "systematic review and meta-analysis"[Title] OR "randomized clinical trial"[Title])
**OpenDissertations (via EBSCO)**	**#1**<br>"chronic kidney" OR "chronic renal" OR "chronic glomerul*" OR "chronic nephro*" OR "progressive kidney" OR "progressive glomerul*" OR "progressive nephro*" OR "diabetic kidney" OR "diabetic renal" OR "diabetic glomerul*" OR ckd OR esrd OR (diabet* AND nephropath*) OR uremi* OR uraemi* OR proteinuri* OR nephrosclerosis OR glomerulosclerosis OR "glomerular sclerosis" OR "Glomerular Filtration Rate" OR microalbuminuri* OR macroalbuminuri* OR albuminuri* OR calciphylaxis OR "secondary hyperparathyroidism" OR "tubulointerstitial fibrosis" OR "interstitial fibrosis" OR "renal fibrosis" OR "kidney fibrosis" OR "vascular calcification*" OR alport* OR denys-drash OR glomerulopathy OR hypoalbuminemi* OR hypoalbuminaemi* OR "multicystic kidney*" OR "polycystic kidney*" OR "cystic kidney*" OR "kidney disease*" OR "kidney failur*" OR "kidney function*" OR "kidney insufficienc*" OR "kidney disorder*" OR "kidney dysfunction" OR "renal disease*" OR "renal failur*" OR "renal function*" OR "renal insufficienc*" OR "renal disorder*" OR "renal dysfunction" OR ((kidney OR renal) AND (ckf OR crd OR crf OR eskd OR eskf OR esrf OR hyperparathyroidism OR end-stage OR endstage OR eGFR)) OR (("kidney transplant*" OR "renal transplant*") AND (candidates OR "wait list*" OR "waiting list*")) OR (("ureteral obstruction" OR nephritis OR glomerulonephritis OR nephrop* OR (obstruct* AND (kidney* OR renal OR nephropathy))) AND (sclerosi* OR fibrosi* OR fibrotic)))<br><br>**#2**<br>#1 AND ("Abdominal Pain" OR "abdominal pai*" OR "abdomen pai*" OR "pain in abdomen" OR "painful abdom*" OR flatulen* OR bloated OR bloating OR diarrhea OR diarrh* OR "loose stoo*" OR "fecal incontinence" OR "faecal incontinenc*" OR "fecal incontinenc*" OR "bowel incontinenc*" OR constipation OR constipat* OR obstipation OR "rectal pain" OR "anorectal pain" OR "functional anorectal" OR defecation OR defaecat* OR defecat* OR "bowel movemen*" OR "Bristol Stool Form" OR "Bristol Stool Scale" OR BSFS OR "gastrointestinal symptom*")
**Scopus**	TITLE-ABS-KEY("chronic kidney") OR TITLE-ABS-KEY("diabetic kidney") OR TITLE-ABS("advanced kidney") OR TITLE-ABS("end stage renal") OR TITLE-ABS("endstage renal") OR TITLE-ABS("end stage kidney") OR TITLE-ABS-KEY("uremic patient*") OR TITLE-ABS-KEY("uraemic patient*") OR TITLE("kidney disease") OR TITLE("renal disease") OR TITLE-ABS("kidney insufficiency") OR TITLE-ABS("renal insufficiency") OR TITLE-ABS("ckd") OR TITLE-ABS("chronic renal") OR TITLE-ABS("progressive kidney") OR TITLE-ABS("progressive uremi*") OR TITLE-ABS("progressive uraemi*") OR (TITLE("chronic*") AND (TITLE("uremi*") OR TITLE("uraemi*"))<br>) OR TITLE-ABS("nephrosclerosis") OR TITLE("glomerulosclerosis") OR TITLE("glomerular sclerosis") OR TITLE-ABS("macroalbuminuri*") OR TITLE-ABS("Renal Osteodystrophy") OR TITLE-ABS("kidney fibrosis") OR TITLE-ABS("renal fibrosis") OR TITLE-ABS("interstitial fibrosis") OR TITLE-ABS("renal hyperparathyroidism") OR TITLE("polycystic kidney*") OR ((TITLE-ABS-KEY("kidney") OR TITLE-ABS-KEY("renal"))<br>AND (TITLE-ABS-KEY("ckf") OR TITLE-ABS-KEY("crd") OR TITLE-ABS-KEY("crf") OR TITLE-ABS-KEY("eskd") OR TITLE-ABS-KEY("eskf") OR TITLE-ABS-KEY("esrf") OR TITLE-ABS-KEY("esrd"))<br>)<br>OR (<br>(TITLE-ABS-KEY("chronic*") OR TITLE-ABS-KEY("severe"))<br>AND (TITLE-ABS-KEY("azotemia") OR TITLE-ABS-KEY("azotaemia"))<br>)<br>OR (TITLE("diabet*") AND (TITLE("nephropath*") OR TITLE("microalbuminuri*") OR TITLE("macroalbuminuri*") OR TITLE("albuminuri*"))<br>)<br>AND (

	TITLE-ABS ("abdominal pai*")<br>OR TITLE-ABS-KEY ("abdomen pai*")<br>OR TITLE-ABS ("pain in abdomen")<br>OR TITLE-ABS ("painful abdom*")<br>OR TITLE-ABS-KEY ("flatulen*")<br>OR TITLE-ABS ("bloated")<br>OR TITLE-ABS ("bloating")<br>OR TITLE-ABS-KEY ("diarrh*")<br>OR TITLE-ABS ("loose stoo*")<br>OR TITLE-ABS-KEY ("faecal incontinenc*")<br>OR TITLE-ABS-KEY ("fecal incontinenc*")<br>OR TITLE-ABS-KEY ("bowel incontinenc*")<br>OR TITLE-ABS-KEY ("constipat*")<br>OR TITLE-ABS-KEY ("obstipation")<br>OR TITLE-ABS-KEY ("rectal pain")<br>OR TITLE-ABS-KEY ("anorectal pain")<br>OR TITLE-ABS ("functional anorectal")<br>OR TITLE-ABS ("defaecat*")<br>OR TITLE-ABS ("defecat*")<br>OR TITLE-ABS ("bowel movemen*")<br>OR TITLE-ABS-KEY ("Bristol Stool Form")<br>OR TITLE-ABS-KEY ("Bristol Stool Scale")<br>OR TITLE-ABS ("BSFS")<br>OR TITLE-ABS ("gastrointestinal symptom*")<br>AND NOT (<br>TITLE ("a case series")<br>OR TITLE ("case report")<br>OR TITLE ("systematic review and meta-analysis")<br>OR TITLE ("randomized clinical trial")<br>OR TITLE-ABS ("ClinicalTrials.gov")<br>OR TITLE ("A randomized trial")<br>OR TITLE ("randomized placebo-controlled trial")<br>OR TITLE ("Efficacy and Safety of")<br>OR TITLE ("a protocol of")<br>OR TITLE ("meta-analysis of randomized")<br>)<br>)<br>AND (<br>EXCLUDE (DOCTYPE,"re")<br>OR EXCLUDE (DOCTYPE,"ed")<br>)
**Web of Science (WoS; in all cases below)**	#1<br>TS=("chronic kidney" OR "chronic renal" OR "chronic glomerul*" OR "chronic nephro*" OR "progressive kidney" OR "progressive glomerul*" OR "progressive nephro*" OR "diabetic kidney" OR "diabetic renal" OR "diabetic glomerul*" OR ckd OR esrd OR (diabet* AND nephropath*) OR uremi* OR uraemi* OR proteinuri* OR nephrosclerosis OR glomerulosclerosis OR "glomerular sclerosis" OR "Glomerular Filtration Rate" OR microalbuminuri* OR macroalbuminuri*

	OR albuminuri* OR calciphylaxis OR "secondary hyperparathyroidism" OR "tubulointerstitial fibrosis" OR "interstitial fibrosis" OR "renal fibrosis" OR "kidney fibrosis" OR "vascular calcification*"<br>OR alport* OR denys-drash OR glomerulopathy OR hypoalbuminemi* OR hypoalbuminaemi* OR "multicystic kidney*" OR "polycystic kidney*" OR "cystic kidney*" OR "kidney disease*"<br>OR "kidney failur*" OR "kidney function*" OR "kidney insufficienc*" OR "kidney disorder*" OR "kidney dysfunction" OR "renal disease*" OR "renal failur*" OR "renal function*"<br>OR "renal insufficienc*" OR "renal disorder*" OR "renal dysfunction" OR<br>((kidney OR renal) AND<br>(ckf OR crd OR crf OR eskd OR eskf OR esrf OR hyperparathyroidism OR end-stage OR endstage OR eGFR))<br>OR<br>(("kidney transplant*" OR "renal transplant*")<br>AND (candidates OR "wait list*" OR "waiting list*"))<br>OR<br>(("ureteral obstruction" OR nephritis OR glomerulonephritis OR nephrop* OR<br>(obstruct* AND (kidney* OR renal OR nephropathy)))<br>AND (sclerosi* OR fibrosi* OR fibrotic))<br>)<br><br>#2<br>TS=("Abdominal Pain" OR "abdominal pai*" OR "abdomen pai*" OR "pain in abdomen" OR "painful abdom*" OR flatulen* OR bloated OR bloating OR diarrhea OR diarrh *<br>OR "loose stoo*" OR "fecal incontinence" OR "faecal incontinenc*" OR "fecal incontinenc*" OR "bowel incontinenc*" OR constipation OR constipat* OR obstipation<br>OR "rectal pain" OR "anorectal pain" OR "functional anorectal" OR defecation OR defaecat* OR defecat* OR "bowel movemen*" OR "Bristol Stool Form"<br>OR "Bristol Stool Scale" OR BSFS OR "gastrointestinal symptom*")<br><br>#3<br>TI = ("Case Series" OR “case report” OR "systematic review and meta-analysis" OR “meta-analysis of randomized” OR “network meta-analysis” OR "randomized clinical trial" OR "randomized placebo-controlled trial" OR "randomized trial" OR “protocol for” OR “protocol of”)<br><br>#4<br>#1 AND #2 NOT #3
**WoS Core Collection (A&HCI, BKCI-SSH, BKCI-S, CCR-EXPANDED, ESCI, CPCI-SSH, CPCI-S, SCI-EXPANDED, SSCI)**	#5<br>#4 **Refined by:** [excluding] **DOCUMENT TYPES:** (REVIEW OR EDITORIAL MATERIAL)
**WoS SciELO**	#5<br>#4 **Refined by**: [excluding] **DOCUMENT TYPES:** (REVIEW ARTICLE OR CASE REPORT)
**WoS Korean Journal Database**	#4

Table S3. Symptom questionnaires used for forward citation chasing

Group of questionnaires (date of citation chasing)	Name of questionnaire	DOI
**Questionnaires not dedicated to CKD population (14-11-2021)**	1. The memorial symptom assessment scale short form (MSAS-SF)	10.1002/1097-0142(20000901)89:5<1162::aid-cncr26>3.0.co;2-y
	2. The Condensed Memorial Symptom Assessment Scale (CMSAS)	10.1081/cnv-200026487
	3. The Memorial Symptom Assessment Scale (MSAS)	10.1016/0959-8049(94)90182-1
	4. Autonomic Symptom Profile (ASP)	10.1212/WNL.52.3.523
	5. The Palliative Care Outcome Scale (POS)	10.1136/qshc.8.4.219
	6. Integrated Palliative care Outcome Scale	10.1177/0269216315608348,
	7. Gastrointestinal Quality of Life Index	10.1002/bjs.1800820229
	8. Constipation Assessment Scale	10.1097/00002820-198906000-00012
	9. Patient Assessment of Constipation Symptoms	10.1080/003655299750025327
	10. Obstructed Defecation Syndrome Score	10.1111/j.1463-1318.2007.01262.x
	11. The NIH Patient-Reported Outcomes Measurement Information System (PROMIS) Gastrointestinal Symptom Scales	10.1038/ajg.2014.237
	12. Upper gastrointestinal disorders-symptom severity index (PAGI-SYM)	10.1007/s11136-004-9567-x
	13. Rome IV Diagnostic Questionnaire	10.1053/j.gastro.2016.02.014
	14-15. Rome III Diagnostic Questionnaires	10.5056/jnm14045, 10.1053/j.gastro.2006.03.008
**Questionnaires dedicated to CKD population (26-12-2021)**	1. Dialysis Symptom Index	10.1681/ASN.2005020157
	2. Patient Outcome Scale-Renal (POS-Renal)	10.1159/000183177
	3. Integrated Palliative Outcome Score (IPOS)-renal	10.1016/j.jpainsymman.2018.04.006
	4. The CKD Symptom Burden Index (CKD SBI)	10.1111/jorc.12152

Table S4. Items of electronic extraction form

Category	Item
**Design and study characteristics**	study design
	First author name
	Journal name
	Geographic coverage (country)
	Study period and year of publication
	Exclusion criteria
**Sample characteristics (extracted for each of the eGFR groups)**	Gender (proportion of males; 0-100)
	Race/ethnicity (proportions)
	CKD etiology (proportions)
	Age
	Creatinine (serum)
	eGFR
	Albuminuria
	Proteinuria
	BMI
**Outcome details (extracted for each of the outcomes)**	Tool used to identify the outcome (name of the questionnaire)
	Min and max values for the numerical scales
	Was the outcome tested for correlation/association with quality of life? Describe.
	Was the outcome tested for correlation/association with lab tests? Describe.
	Was the outcome tested for correlation/association with clinical data? Describe.
	Is information about prevalence in the control group (without CKD) collected/available?
**Risk of bias assessment**	JBI Critical Appraisal Checklist for Studies Reporting Prevalence Data
**Results (extracted for each of the eGFR groups and outcomes)**	Total (N analyzed)
	The number of participants reporting the outcome (cases)
	Mean/median, SD/IQR, range for severity outcomes evaluated with numerical scales

Table S5. Excluded studies with reason

Title	year	journal	Volume (issue)	First author	Decision	Source of record*
A study of gastric emptying in chronic renal failure	1997	Journal of Association of Physicians of India	45 (11)	Alimchandani, A.	WRONG OUTCOME (fullness instead of bloating or abdominal pain)	DS
Application of immunosuppressants in patients with autosomal dominant polycystic kidney disease after kidney transplantation	2020	Nan fang yi ke da xue xue bao	40 (4)	Li, Q.	WRONG OUTCOME (aggregated data for all gastrointestinal symptoms such as nausea, vomiting, diarrhea, and flatulence)	DS
Association of Constipation with risk of end-stage renal disease in patients with chronic kidney disease.	2019	BMC nephrology	20 (1)	Lu CY	WRONG OUTCOME (registry-based)	DS
Associations between multimorbidity and glycaemia (HbA1c) in people with type 2 diabetes: Cross-sectional study in Australian general practice	2020	BMJ Open	10 (11)	Chiang, J.I.	WRONG OUTCOME (registry-based)	DS
Asymptomatic pyuria in diabetic women.	2001	Nippon Ika Daigaku zasshi	68 (5)	Nakano H	WRONG POPULATION (lack of separate analysis for diabetic nephropathy) and OUTCOME (constipation treatment instead of reporting symptom)	DS
Autonomic neuropathy is associated with increased cardiovascular risk factors: the EURODIAB IDDM Complications Study.	2002	Diabetic medicine	19 (11)	Kempler P	WRONG OUTCOME (nocturnal diarrhea instead of diarrhea) and POPULATION (lack of separate analysis for patients with diabetic nephropathy)	DS
Autosomal dominant polycystic kidney disease: MR imaging evaluation using current techniques	2003	Journal of Magnetic Resonance Imaging	18 (2)	Mosetti, M.A.	WRONG OUTCOME (indication for the magnetic resonance examination instead of patients' symptoms)	DS
Autosomal dominant polycystic kidney disease: observations from a university hospital in Saudi Arabia.	1995	Saudi journal of kidney diseases and transplantation	6 (1)	Al-Muhanna FA	WRONG OUTCOME (abdominal pain assessed based on retrospective medical records analysis only)	DS
Autosomal dominant polycystic kidney disease: Study of clinical characteristics in an Indian population.	2017	Saudi journal of kidney diseases and transplantation	28 (1)	Vikrant S	WRONG OUTCOME ("Lumbar or abdominal pain" instead of abdominal pain)	DS
Autosomal dominant polycystic kidney disease: symptoms and clinical findings.	1984	The Quarterly journal of medicine	53 (212)	Milutinovic J	WRONG OUTCOME ("back pain", "abdominal tenderness and fullness")	DS
Baseline graft status is a critical predictor of kidney graft failure after diarrhoea.	2019	Nephrology, dialysis, transplantation	34 (9)	Devresse A	WRONG POPULATION (only symptomatic patients, lack of subanalysis for outpatients)	DS
Burden of drug use for gastrointestinal symptoms and functional gastrointestinal disorders in France: a national study using reimbursement data for 57 million inhabitants.	2019	Therapeutic advances in gastroenterology	12	Tuppin P	WRONG POPULATION (dialysis aggregated with transplanted patients)	DS
Characteristics and Dysbiosis of the Gut Microbiome in Renal Transplant Recipients.	2020	Journal of clinical medicine	9 (2)	Swarte JC	WRONG OUTCOME (symptoms prevalence not assessed)	DS

Characteristics of the patients with diabetic nephropathy with relatively low serum creatinine at the initiation of dialysis.	1990	Nihon Jinzo Gakkai shi	32 (9)	Nakao T	WRONG OUTCOME (aggregated prevalence of gastrointestinal symptoms that was a reason for introduction dialysis)	DS
Characterization of Upper Gastrointestinal Symptoms, Gastric Motor Functions, and Associations in Patients with Diabetes at a Referral Center.	2019	The American journal of gastroenterology	114 (1)	Chedid V	WRONG POPULATION (lack of separate analysis for patients with diabetic nephropathy)	DS
Chronic constipation is negatively associated with colonic diverticula	2021	Scandinavian Journal of Gastroenterology	56 (11)	Higashimori, A.	WRONG POPULATION (lack of separate analysis for non-dialysis patients with CKD)	DS
Clinical and pathological spectrums of aristolochic acid nephropathy.	2012	Clinical nephrology	78 (1)	Chen D	WRONG POPULATION (both AKI and CKD) and WRONG OUTCOME (aggregated data on nausea, vomiting and poor appetite)	DS
Clinical factors associated with the symptoms of constipation in patients with diabetes mellitus: A multicenter study.	2018	Journal of gastroenterology and hepatology	33 (4)	Yamada E	WRONG POPULATION (lack of separate analysis for patients with diabetic nephropathy)	DS
Clinical Features of 167 Inpatients with Autosomal Dominant Polycystic Kidney Disease at a Single Center in China.	2018	Medical science monitor	24	Meng J	WRONG OUTCOME ("Lumbar and/or abdominal pain" and "flank pain" instead of abdominal pain)	DS
Clinical manifestations of autosomal dominant polycystic kidney disease in patients older than 50 years.	1990	American journal of kidney diseases	15 (3)	Milutinovic J	WRONG OUTCOME ("frequent or constant" back/abdominal tenderness after exclusion of "gastrointestinal and gynecological causes" was assessed instead of abdominal pain)	DS
Clinical manifestations, risk factors, and prognostic factors of cytomegalovirus enteritis.	2021	Gut pathogens	13 (1)	Yeh PJ	WRONG POPULATION (inpatients; CKD only in 31% of participants; lack of subgroup analysis)	DS
Clinical remarks on diarrhea and vomiting, the result of renal disease	1867	British Medical Journal	1 (330)	Johnson, G.	WRONG PUBLICATION TYPE (letter)	DS
Clinical study on thyroid function in chronic renal failure	1983	The Japanese Journal of Nephrology	25 (8)	Kijima, Y.	WRONG POPULATION (symptoms evaluated only amongst dialysis patients)	DS
Colonic changes in uremia	1981	Union Medicale du Canada	110 (5)	Caron, C.	WRONG POPULATION (both dialysis and non-dialysis ESKD patients)	DS
Colonic diverticular disease in autosomal dominant polycystic kidney disease: is there really an association? A nationwide analysis.	2021	International journal of colorectal disease	36 (1)	Duarte-Chavez R	WRONG OUTCOME (registry-based)	DS
Comorbidity and polypharmacy in chronic heart failure: A large cross-sectional study in primary care	2017	British Journal of General Practice	67 (658)	Baron-Franco, B.	WRONG OUTCOME (registry-based)	DS
Comorbidity Burden in Adults With Autism Spectrum Disorders and Intellectual Disabilities—A Report From the EFAAR (Frailty Assessment in Ageing Adults With Autism Spectrum and Intellectual Disabilities) Study	2019	Frontiers in Psychiatry	10	Miot, S.	WRONG POPULATION (not CKD)	DS

Comparison of High-Resolution Manometry in Patients Complaining of Dysphagia among Patients with or without Diabetes Mellitus.	2021	Digestion	102 (4)	Muroi K	WRONG POPULATION (lack of subgroup analysis for patients with diabetic nephropathy)	DS
Constipation In Chinese Elderly: A Hidden Cause Of Chronic Kidney Disease And The Risk Of Rapid Renal Function Decline	2020	Nephrology Dialysis Transplantation	35	Jian, GH	WRONG OUTCOME (registry based study; unclear definition of constipation)	DS
Constipation might be associated with risk of allergic rhinitis: A nationwide population-based cohort study.	2020	PloS one	15 (10)	Wu MC	WRONG OUTCOME (registry-based)	DS
Constipation, hard stools, fecal urgency, and incomplete evacuation, but not diarrhea is associated with diabetes and its related factors.	2016	World journal of gastroenterology	22 (11)	Ihana-Sugiyama N	WRONG SETTINGS (inpatients rather than outpatients)	DS
Conversion to mycophenolate mofetil in conjunction with stepwise withdrawal of cyclosporine in stable renal transplant recipients.	2000	Transplantation	69 (3)	Schrama YC	WRONG OUTCOME (instead of diarrhea/other symptoms prevalence assessment, only severe, probably MMF-associated, diarrhea cases were reported)	DS
Conversion to sirolimus-based maintenance immunosuppression using daclizumab bridge therapy in renal transplant recipients.	2004	Clinical transplantation	18	Sundberg AK	WRONG OUTCOME ("diarrhea" not defined; period prevalence of adverse effect instead of point prevalence of symptom)	DS
Correlation of Cognitive Impairment with Constipation and Renal Failure	2016	Sains Malaysiana	45 (9)	Eshkoor, SA	Wrong population ("renal failure" was "recorded based on both respondents report and physician's diagnosis"; lack of lab test or eGFR estimation)	DS
Cross-sectional study of quality of life and symptoms in chronic renal disease patients: The Modification of Diet in Renal Disease Study	1997	American Journal of Kidney Diseases	29 (6)	Rocco, M.V.	WRONG OUTCOME (Prevalence and severity of constipation, diarrhea, and abdominal bloating were assessed but not reported; authors reported "severity index score" (result of multiplication the frequency by the severity of each symptom") of abdominal bloating	DS
Cryptosporidium spp. Infection in Solid Organ Transplantation: The Nationwide "TRANSCRYPTO" Study.	2017	Transplantation	101 (4)	Lanternier F	WRONG POPULATION (mixed of kidney-, liver-, heart-, and pancreas-transplant patients)	DS
Decreased concentrations of deoxycholic acid in serum of uraemic patients with diarrhoea.	1990	Scandinavian journal of clinical and laboratory investigation	50 (3)	Stenvinkel P	WRONG POPULATION (mixed of 16 dialysis and 2 non-dialysis CKD patients)	DS
Delayed gastric emptying among Indian patients with non-diabetic chronic kidney disease	2021	Indian Journal of Nephrology	31 (2)	Kumar, M.S.	WRONG POPULATION (mix of both dialysis- and non-dialysis patients) and OUTCOME (GCSI subscale instead of 1-item symptom)	DS
Delayed gastric emptying in patients with chronic renal failure	1996	Nuclear Medicine Communications	17 (2)	Kao, C.H.	WRONG OUTCOME (aggregated presence of upper gastrointestinal symptoms was only reported)	DS
Delayed Gastric Emptying Is Associated With Early and Long-term Hyperglycemia in Type 1 Diabetes Mellitus.	2015	Gastroenterology	149 (2)	Bharucha AE	WRONG POPULATION (lack of separate analysis for patients with diabetic nephropathy)	DS

Demographic, diagnostic and therapeutic characteristics of autosomal dominant polycystic kidney disease in Ghana.	2021	BMC nephrology	22 (1)	Okyere P	WRONG POPULATION (both AKI and CKD)	DS
Diabetic factors associated with gastrointestinal symptoms in patients with type 2 diabetes.	2010	World journal of gastroenterology	16 (14)	Kim JH	WRONG POPULATION (lack of subgroup analysis for patients with diabetic nephropathy)	DS
Diarrhea In A Patient With Renal-Failure	1976	Minnesota Medicine	59 (1)	Tsai, SH	WRONG PUBLICATION TYPE (case report)	DS
Dietary intake and nutritional deficiencies in patients with diabetic or idiopathic gastroparesis.	2011	Gastroenterology	141 (2)	Parkman HP	WRONG POPULATION (lack of separate analysis for diabetic nephropathy)	DS
Dyspepsia among patients with chronic kidney disease: a cross sectional study.	2013	International archives of medicine	6 (1)	Bacci MR	WRONG OUTCOME (dyspepsia)	DS
Effect of tolerance versus chronic immunosuppression protocols on the quality of life of kidney transplant recipients.	2016	JCI insight	1 (8)	Madariaga ML	WRONG OUTCOME (prevalence of lower GI symptoms was not reported)	DS
Epigenetic Alterations Are Associated With Gastric Emptying Disturbances in Diabetes Mellitus.	2020	Clinical and translational gastroenterology	11 (3)	Puthanmadhom Narayanan S	WRONG POPULATION (lack of separate analysis for patients with diabetic nephropathy)	DS
Evaluation of E148Q and Concomitant AA Amyloidosis in Patients with Familial Mediterranean Fever.	2021	Journal of clinical medicine	10 (16)	Arici ZS	WRONG OUTCOME (unclear whether point or period prevalence was reported; unclear definition of symptoms/methods of assessment)	DS
Evaluation of tolerance and safety of conversion from mycophenolate mofetil to enteric-coated mycophenolic acid in renal transplant recipients.	2017	Journal of biological regulators and homeostatic agents	31 (1)	Qiao LW	WRONG POPULATION (only symptomatic kidney transplanted patients)	DS
Evaluation of upper gastrointestinal symptoms and effect of different modalities of treatment in patients of chronic kidney disease	2014	Journal, Indian Academy of Clinical Medicine	15 (3)	Nand, N.	WRONG POPULATION (only symptomatic patients) AND OUTCOME (lack of report of associations between abdominal pain and any clinical/lab findings)	DS
Exocrine pancreatic dysfunction is common in hepatocyte nuclear factor 1β-associated renal disease and can be symptomatic.	2018	Clinical kidney journal	11 (4)	Clissold RL	WRONG OUTCOME (aggregated data on abdominal pain, loose stools or unintentional weight loss)	DS
Familial Mediterranean fever in Mexico City. A 20-year follow-up.	2004	Cirugia y cirujanos	72 (2)	Halabe-Cherem J	WRONG POPULATION (lack of patients with chronic kidney disease	DS
Gastric emptying in chronic renal failure	1985	British Medical Journal (Clinical research ed.)	291 (6491)	McNamee, P.T.	WRONG OUTCOME (e.g., gastric emptying, nausea, vomiting)	DS
Gastric helicobacter and upper gastrointestinal symptoms in chronic renal failure	1991	Annals of Medicine	23 (4)	Ala-Kaila, K.	WRONG OUTCOME (even though "all renal patients were systematically questioned as to upper GI symptoms such as heartburn or upper gastric pain relieved by milk or antacids", prevalence of symptoms was not reported)	DS

Gastroduodenal lesions and Helicobacter pylori infection in dyspeptic patients with and without chronic renal failure	2005	Helicobacter	10 (1)	Nardone, G.	WRONG POPULATION (mixed non-dialysis and dialysis CKD patients)	DS
Gastroesophageal reflux disease in chronic renal failure patients: evaluation by endoscopic examination.	2009	The Tokai journal of experimental and clinical medicine	34 (3)	Kawaguchi Y.	WRONG OUTCOME (not detailed, "some upper GI symptoms")	DS
Gastrointestinal complications in patients with chronic kidney disease--a 5-year retrospective study from a tertiary referral center.	2013	Renal failure	35 (1)	Thomas R	WRONG POPULATION (only symptomatic CKD patients; hemodialysis patients mixed with non-transplanted CKD patients)	DS
Gastrointestinal symptoms and shock in a patient with chronic renal failure	1980	The American Journal of Medicine	69 (4)		WRONG PUBLICATION TYPE (case report)	DS
Gastrointestinal transit disorders in patients with insulin-treated diabetes mellitus.	1990	Digestive diseases (Basel, Switzerland)	8 (1)	Wegener M	WRONG POPULATION (lack of separate analysis for diabetic nephropathy) and SETTINGGS (inpatients)	DS
GASTROPATHIES IN PATIENTS WITH 3-4 STAGES OF CHRONIC KIDNEY DISEASE.	2016	Eksperimental'naia i klinicheskaia gastroenterologiia	(10)	Abdurakhmanova NM	WRONG OUTCOME (only upper gastrointestinal symptoms were evaluated)	DS
Genetic Polymorphisms Affecting Tacrolimus Metabolism and the Relationship to Post-Transplant Outcomes in Kidney Transplant Recipients.	2021	Pharmacogenomics and personalized medicine	14	Cheng F	WRONG OUTCOME (unclear definition and assessment of diarrhea)	DS
Health impact of acute intermittent porphyria in latent and non-recurrent attacks patients.	2021	Orphanet journal of rare diseases	16 (1)	Buendía-Martínez J	WRONG POPULATION (lack of subgroup analysis for 8 CKD patients)	DS
Helicobacter pylori eradication for the treatment of dyspeptic symptoms in chronic renal failure	2005	Annals of Saudi Medicine	25 (5)	Šimunić, M	WRONG OUTCOME (probably epigastric rather than abdominal pain)	DS
Henoch-Schönlein purpura in adults. a study of 40 cases	1996	Revue de Medecine Interne	17 (5)	Lasseur, C.	WRONG OUTCOME (unclear definition of diarrhea, probably based on medical records)	DS
Histomorphological patterns of renal amyloidosis: A correlation between histology and chemical type of amyloidosis	1997	Human Pathology	28 (7)	Looi, L.-M.	WRONG OUTCOME (GI symptoms not assessed in all patients)	DS
Immunoglobulin light chain amyloidosis is diagnosed late in patients with preexisting plasma cell dyscrasias.	2014	American journal of hematology	89 (11)	Kourelis TV	WRONG POPULATION (lack of subgroup analysis for CKD patients) and OUTCOME ("earliest referable symptom or abnormal laboratory value" instead of symptom prevalence)	DS
Impact of conversion to a once daily tacrolimus-based regimen in kidney transplant recipients with gastrointestinal complications.	2012	Transplantation	93 (9)	Veroux M	WRONG POPULATION (only symptomatic kidney transplanted patients)	DS

Impaired gastric motility and its relationship to gastrointestinal symptoms in patients with chronic renal failure.	2005	Journal of gastroenterology	40 (12)	Hirako M	WRONG OUTCOME (assessed but not reported: "All patients completed a self-administered questionnaire that included seven symptoms (anorexia, nausea, heartburn, abdominal pain, bloating, diarrhea, and constipation)")	DS
Improvement in autonomic and gastric function following pancreas-kidney versus kidney-alone transplantation and the correlation with quality of life.	1994	Transplantation	57 (6)	Hathaway DK	WRONG OUTCOME (abdominal pain and bloating assessed but reported as composite score of gastrointestinal symptoms)	DS
Incidence and Causes of Late Hospital Readmissions After Living Donor Renal Transplant: A Retrospective Study.	2021	Experimental and clinical transplantation	19 (5)	Sharma A	WRONG OUTCOME (diarrhea as a hospital admission reason)	DS
Increased prevalence of gastrointestinal symptoms in patients with chronic renal failure.	2000	Gastroenterology	118 (4)	Strid, H	WRONG OUTCOME (prevalence of GI symptoms not reported)	DS
Late conversion to mammalian target of rapamycin inhibitor/proliferation signal inhibitors in kidney transplant patients: clinical experience in the last 5 years.	2010	Transplantation proceedings	42 (8)	Sola E	WRONG OUTCOME (aggregated data on all gastrointestinal symptoms)	DS
Laxative Use and Change in Estimated Glomerular Filtration Rate in Patients With Advanced Chronic Kidney Disease.	2020	Journal of renal nutrition	31 (4)	Sumida K	WRONG OUTCOME (registry based)	DS
Laxative Use and Risk of Dyskalemia in Patients with Advanced CKD Transitioning to Dialysis.	2021	Journal of the American Society of Nephrology	32 (4)	Sumida K	WRONG OUTCOME (registry based)	DS
Laxative use in patients with advanced chronic kidney disease transitioning to dialysis.	2020	Nephrology, dialysis, transplantation	36 (11)	Sumida K	WRONG OUTCOME (registry based)	DS
Long-term outcome of live donor kidney transplantation for renal amyloidosis	2003	American Journal of Kidney Diseases	42 (2)	Sherif, A.M.	WRONG OUTCOME (aggregated data on nausea, vomiting, diarrhea, and abdominal pain)	DS
Long-term outcomes of lupus nephritis treated with regimens based on cyclophosphamide and mycophenolate mofetil	2020	Lupus	29 (8)	Prasad, N.	WRONG OUTCOME ("diarrhea" not defined; period prevalence of adverse effect instead of point prevalence of symptom)	DS
Microsporidia infection among various groups of the immunocompromised patients.	2018	Tropical biomedicine	35 (2)	Hassan NA	WRONG POPULATION (ESKD not specified - unclear whether there were dialysis patients included)	DS
Nephrological manifestations of patients with Fabry disease in Argentina	2007	Revista de Nefrologia, Dialisis y Trasplante	27 (3)	Marcelo Neumann, P	WRONG POPULATION (lack of subgroup analysis for non-dialysis CKD patients)	DS
Outcomes and Factors Associated With Reduced Symptoms in Patients With Gastroparesis.	2015	Gastroenterology	149 (7)	Pasricha PJ	WRONG POPULATION (lack of separate analysis for diabetic nephropathy)	DS
Pain patterns in patients with polycystic kidney disease	2004	Kidney International	66 (4)	Bajwa, Z.H.	WRONG POPULATION (aggregated data for both non-dialysis, dialysis, and post-transplant patients)	DS
Patients with chronic renal failure have abnormal small intestinal motility and a high prevalence of small intestinal bacterial overgrowth.	2003	Digestion	67 (3)	Strid H	WRONG POPULATION (mix of both dialysis- and non-dialysis patients)	DS
Prevalence and evaluation of symptoms in advanced chronic kidney disease	2015	Enfermeria Nefrologica	18 (3)	Gutiérrez Sánchez D	WRONG PUBLICATION TYPE (Review)	DS

Prevalence and pattern of cystic kidney diseases in Ilorin, Nigeria.	2010	Saudi journal of kidney diseases and transplantation	21 (6)	Chijioke, A.	WRONG OUTCOME (abdominal pain as a presenting feature, not a systematically assessed symptom) AND CONTEXT (inpatients)	DS
Prevalence and predictors of delayed gastric emptying among Indian patients with long-standing type 2 diabetes mellitus.	2016	Indian journal of gastroenterology	35 (5)	Anudeep V	WRONG POPULATION (lack of separate analysis for patients with diabetic nephropathy)	DS
Prevalence of amyloid deposition in long standing rheumatoid arthritis in Iranian patients by abdominal subcutaneous fat biopsy and assessment of clinical and laboratory characteristics.	2006	BMC musculoskeletal disorders	7	Alishiri GH	WRONG POPULATION (lack of separate analysis for CKD patients/patients with renal amyloidosis)	DS
Prevalence of Chronic Constipation and Chronic Diarrhea in Diabetic Individuals in the United States.	2019	The American journal of gastroenterology	114 (1)	Sommers T	WRONG POPULATION (Diabetic patients. Neither creatinine nor proteinuria was collected. CKD was defined as "Told you had weak/failing kidneys")	DS
Prevalence of diarrhea in end-stage renal disease patients initiating hemodialysis	2021	Renal Replacement Therapy	7 (1)	Oba, M.	WRONG POPULATION (inpatients with both acute and chronic kidney disease)	DS
Prevalence of gastrointestinal symptoms in patients with chronic obstructive pulmonary disease.	2008	European journal of gastroenterology & hepatology	20 (4)	Niklasson A	WRONG OUTCOME (GSRS subscale instead of symptom)	DS
Prevalence of gastrointestinal symptoms in young and middle-aged diabetic patients.	1999	Scandinavian journal of gastroenterology	34 (12)	Spångéus A	WRONG POPULATION (lack of reported separate analysis for diabetic nephropathy)	DS
Prevalence of Helicobacter pylori in patients with different renal function	1997	Turkish Journal of Gastroenterology	8 (3)	Vardar, R.	WRONG OUTCOME (lack of data on the symptom prevalence)	DS
Prevalence of Intestinal Protozoa among Saudi Patients with Chronic Renal Failure: A Case-Control Study.	2015	Journal of tropical medicine	2015	Hawash YA	WRONG POPULATION (it is not clear whether dialysis patients were included)	DS
Prevalence of symptoms in female Fabry disease patients: a case-control survey.	2012	Journal of inherited metabolic disease	35 (5)	Bouwman MG	WRONG POPULATION (lack of information about chronic kidney disease)	DS
Prevalence of the need for sodium intake restriction and the use of laxatives in palliative patients	2018	Revista Espanola de Enfermedades Digestivas	110 (11)	Gándara-Del-Castillo, Á.	WRONG POPULATION (mixed of CKD and other palliative patients) and WRONG OUTCOME (laxative usage)	DS
Primary IgA glomerulonephritis and Schönlein-Henoch purpura nephritis: Clinicopathological and immunohistological characteristics.	1978	The Quarterly journal of medicine	47 (188)	Nakamoto Y	WRONG OUTCOME (only "abdominal pain" evaluated based on medical records)	DS
Quality of life in renal transplant recipients following conversion from mycophenolate mofetil to enteric-coated mycophenolate sodium.	2007	Transplantation proceedings	39 (7)	Cofan F	WRONG POPULATION (only symptomatic kidney transplanted patients)	DS
Racial differences in symptoms and complications in adults with type 2 diabetes mellitus.	1999	Ethnicity & health	4 (1)	Konen JC	WRONG POPULATION (lack of separate analysis for diabetic nephropathy)	DS
Renal function and symptoms/adverse effects in opioid-treated patients with cancer.	2015	Acta anaesthesiologica Scandinavica	59 (8)	Kurita GP	WRONG POPULATION (advance cancer population)	DS

Retrospective analysis of the overt proteinuria diabetic kidney disease in the treatment of modified Shenzhuo formula for 2 years	2017	Medicine (United States)	96 (12)	Chen, H	WRONG OUTCOME (prevalence of "chief complaint"/"the main symptoms of interrogation" were evaluated instead of systematic assessment of lower GI symptoms)	DS
Role of Endoscopic Findings and Biopsies in Renal Transplant Recipients With Gastrointestinal Complications: A Tertiary Care Experience.	2018	Experimental and clinical transplantation	16 (5)	Wadhwa RK	WRONG OUTCOME (diarrhea as an indication for endoscopy, not as a symptom; aggregated endoscopy findings independently of indication)	DS
Safety of Eplerenone for Kidney-Transplant Recipients with Impaired Renal Function and Receiving Cyclosporine A.	2016	PloS one	11 (4)	Bertocchio JP	WRONG OUTCOME (gastrointestinal symptoms were not systematically assessed)	DS
Saliva Composition And Upper Gastrointestinal Symptoms In Chronic Renal Disease	2012	NEPHROLOGY	17	Manley, K	WRONG OUTCOME (only upper gastrointestinal symptoms)	DS
Screening for celiac disease among patients with chronic kidney disease.	2012	Renal failure	34 (5)	Sahin I	WRONG OUTCOME (prevalence of symptoms were not assessed)	DS
Serum-Mediated Inhibition of Enzyme Replacement Therapy in Fabry Disease.	2016	Journal of the American Society of Nephrology : JASN	27 (1)	Lenders M	WRONG POPULATION (mixed of non-dialysis, dialysis, and transplanted patients)	DS
Signs and symptoms in chronic renal failure. II. Vomiting, twitching, haemorrhagic diathesis, convulsions, itching, and diarrhoea.	1958	Acta medica Scandinavica	160 (5)	Effersoe P	WRONG PUBLICATION TYPE (case series)	DS
Study of live donor kidney transplantation outcome in recipients with renal amyloidosis.	1994	Nephrology, dialysis, transplantation	9 (6)	Sobh M	WRONG OUTCOME (aggregated data on nausea, vomiting, abdominal pains and diarrhoea)	DS
Symptom-Based Stratification of Diabetes Mellitus by Renal Function Decline (SYSTEM): A Retrospective Cohort Study and Modeling Assessment.	2021	Frontiers in medicine	8	Chan KW	WRONG POPULATION (lack of subanalysis for patients with CKD/diabetic nephropathy) and OUTCOME ("Abdominal distension" instead of bloating; "Alternating dry or loose stool" instead of constipation/diarrhea)	DS
Taste genetics and gastrointestinal symptoms experienced in chronic kidney disease.	2015	European journal of clinical nutrition	69 (7)	Manley KJ	WRONG OUTCOME ("dry mouth, taste changes, nausea, dry retching and vomiting")	DS
The association of combined total kidney and liver volume with pain and gastrointestinal symptoms in patients with later stage autosomal dominant polycystic kidney disease	2017	American Journal of Nephrology	46 (3)	D'Agnolo, H.M.A.	WRONG OUTCOME (upper and lower abdominal pain assessed separately)	DS
The clinical features and outcomes of systemic AL amyloidosis: A cohort of 231 Chinese patients	2015	Clinical Kidney Journal	8 (1)	Huang, X.	WRONG POPULATION (lack of separate analysis for patients with CKD) and WRONG OUTCOME ("recurrent diarrhea" instead of diarrhea)	DS
The Gastrointestinal-Tract In Uremia	1993	Digestive Diseases And Sciences	38 (2)	Kang, JY	WRONG PUBLICATION TYPE (narrative review)	DS
The impact of gastroesophageal reflux disease, irritable bowel syndrome, and functional constipation on health-related quality of life in patients with chronic kidney disease	2018	Journal Of Gastroenterology And Hepatology	33	Nor, NM	WRONG POPULATION (mixed with dialysis patients)	DS

The prevalence of gastrointestinal symptoms in patients with chronic renal failure is increased and associated with impaired psychological general well-being	2002	Nephrology Dialysis Transplantation	17 (8)	Strid, H.	WRONG OUTCOME (GSRS subscale instead of symptom)	DS
Tolerability of mycophenolate sodium in renal transplant recipients	2018	International Journal of Clinical Pharmacy	40 (6)	Hiramoto, L.L.	WRONG OUTCOME (diarrhea as one of the reason for mycophenolate dose change, not evaluated as a symptom)	DS
Unilateral multicystic renal disease in adults	1982	Journal of Urology	128 (2)	Ambrose, S.S.	WRONG PUBLICATION TYPE (case series)	DS
Upper gastro-intestinal mucosal changes in patients with chronic renal failure.	1989	The Journal of the Association of Physicians of India	37 (9)	Goenka MK	WRONG OUTCOME (prevalence of nausea, vomiting, anorexia and GI bleeding was reported)	DS
Uraemic Diarrhea.	1869	British medical journal	2 (464)	Forthergill JM	WRONG PUBLICATION TYPE (letter)	DS
Uremia And Abdominal Pain	1968	Postgraduate Medicine	44 (4)	Donnelly, WJ	WRONG PUBLICATION TYPE (case report)	DS
Uremigenic diarrheas.	1957	Acta gastro-enterologica Belgica	20 (11)	Froehlich AL	WRONG PUBLICATION TYPE (case series)	DS
Validation of the IPOS-Renal Symptom Survey in Advanced Kidney Disease: A Cross-sectional Study	2018	Journal of Pain and Symptom Management	56 (2)	Raj, R.	WRONG POPULATION (lack of separate analysis for non-dialysis patients)	DS
Variable clinical features of patients with Fabry disease and outcome of enzyme replacement therapy	2021	Molecular Genetics and Metabolism Reports	26	Dutra-Clarke, M.	WRONG POPULATION (lack of separate analysis for patients with CKD) and OUTCOME (aggregated abdominal pain, constipation, and diarrhea)	DS
CKD in Elderly Patients Managed without Dialysis: Survival, Symptoms, and Quality of Life	2015	Clinical journal of the American Society of Nephrology	10 (2)	Brown, Mark A.	WRONG OUTCOME (prevalence not reported)	CCQ (not dedicated to CKD)
Delayed gastric emptying associates with diabetic complications in diabetic patients with symptoms of gastroparesis	2019	The American journal of gastroenterology	114 (11)	Parkman, Henry P.	WRONG POPULATION (only symptomatic patients)	CCQ (not dedicated to CKD)
Delayed Gastric Emptying Is Associated With Early and Long-term Hyperglycemia in Type 1 Diabetes Mellitus.	2015	Gastroenterology	149 (2)	Bharucha, A.E.	WRONG POPULATION (lack of separate analysis for patients with diabetic nephropathy)	CCQ (not dedicated to CKD)
Development and validation of a specific questionnaire for evaluating the impact of gastrointestinal symptoms on the health-related quality of life of transplant patients.	2012	Transplantation proceedings	44 (5)	Ortega, F.	WRONG POPULATION (patients after transplantation of different organs) and OUTCOME (prevalence not reported)	CCQ (not dedicated to CKD)
Epigenetic Alterations Are Associated With Gastric Emptying Disturbances in Diabetes Mellitus.	2020	Clinical and translational gastroenterology	11 (3)	Narayanan, S.P.	WRONG POPULATION (lack of separate analysis for patients with diabetic nephropathy)	CCQ (not dedicated to CKD)
Factors affecting diabetic patient's long-term quality of life after simultaneous pancreas-kidney transplantation: a single-center analysis.	2021	Langenbeck's archives of surgery	406 (3)	López-Sánchez, J.	WRONG OUTCOME (GI symptoms prevalence were not assessed)	CCQ (not dedicated to CKD)

Opting not to dialyse : a practitioner research study to explore patient experience	2009	NA		Noble, H.	WRONG OUTCOME (GI symptoms prevalence not assessed systematically)	CCQ (not dedicated to CKD)
Patient-reported gastrointestinal symptom burden and health-related quality of life following conversion from mycophenolate mofetil to enteric-coated mycophenolate sodium.	2006	Transplantation	81 (9)	Chan, L.	WRONG OUTCOME (GSRS subscales instead of GI symptoms prevalence were reported)	CCQ (not dedicated to CKD)
Reduction of gastrointestinal complications in renal graft recipients after conversion from mycophenolate mofetil to enteric-coated mycophenolate sodium.	2011	Transplantation proceedings	43 (5)	Reinke, P.	WRONG OUTCOME (GSRS subscales instead of GI symptoms prevalence were reported)	CCQ (not dedicated to CKD)
The Maastricht Study: an extensive phenotyping study on determinants of type 2 diabetes, its complications and its comorbidities	2014	European journal of epidemiology	29 (6)	Schram, M.T.	WRONG OUTCOME (GI symptoms prevalence not reported)	CCQ (not dedicated to CKD)
"An evil heritage": interview study of pain and autosomal dominant polycystic kidney disease.	2009	Pain management nursing	10 (3)	Heiwe, S.	WRONG OUTCOME (lower GI symptoms prevalence were not assessed)	CCQ (nephrological)
A brief, patient- and proxy-reported outcome measure in advanced illness: Validity, reliability and responsiveness of the Integrated Palliative care Outcome Scale (IPOS).	2019	Palliative medicine	33 (8)	Murtagh, F.E.M.	WRONG POPULATION (lack of subgroup analysis for CKD patients)	CCQ (nephrological)
A Mixed Methods Study of Symptom Experience in Patients With End-Stage Renal Disease.	2020	Nursing research	70 (1)	Ng, M.S.N.	WRONG POPULATION (dialysis-dependent patients)	CCQ (nephrological)
Arabic translation, adaptation and modification of the dialysis symptom index for chronic kidney disease stages four and five	2015	BMC nephrology	16 (1)	Almutary, H.	WRONG OUTCOME (prevalence not reported)	CCQ (nephrological)
Assessment of palliative need in patients with chronic kidney disease by the new Three Levels of Need Questionnaire (3LNQ) is not exhaustive.	2014	Danish medical journal	61 (4)	Blindbaek, L.	WRONG OUTCOME (lower GI symptoms prevalences were not assessed)	CCQ (nephrological)
Bridging "Office-Based Care" With the "Virtual Practice Care Model": Evolving Care for Chronic Kidney Disease Patients in the COVID-19 Pandemic-And Beyond.	2020	Frontiers in medicine	7	Zhao, B.	WRONG OUTCOME (prevalence of GI symptoms not reported)	CCQ (nephrological)
Changes in symptom burden and physical performance with initiation of dialysis in patients with chronic kidney disease.	2014	Hemodialysis international.	19 (1)	Rivara, M.B.	WRONG OUTCOME (lower GI symptoms prevalences were not assessed)	CCQ (nephrological)
Course of symptoms and health-related quality of life during specialized pre-dialysis care.	2014	PloS one	9 (4)	de Goeij, M.C.M.	WRONG OUTCOME (lower GI symptoms prevalences were not assessed)	CCQ (nephrological)
Developing a self-administered CKD symptom assessment instrument.	2009	Nephrology, dialysis, transplantation	25 (1)	Agarwal, R.	WRONG OUTCOME ("Difficult bowel movements" instead of constipation, "Frequent bowel movements" instead of diarrhea; prevalence not reported)	CCQ (nephrological)

Development and usability testing of an electronic patient-reported outcome measure (ePROM) system for patients with advanced chronic kidney disease.	2018	Computers in biology and medicine	101	Aiyegbusi, O.L.	WRONG OUTCOME (prevalence of GI symptoms was not reported)	CCQ (nephrological)
Differences in illness representations in patients with chronic kidney disease.	2015	Journal of renal care	41 (3)	Pagels, A.A.	WRONG OUTCOME ("changed bowel habits" instead of either constipation or diarrhea)	CCQ (nephrological)
Differences in physical symptoms between those with and without kidney disease: a comparative study across disease stages in a UK population.	2021	BMC nephrology	22 (1)	Wilkinson, T. J.	WRONG OUTCOME (lower GI symptoms prevalences were not assessed)	CCQ (nephrological)
Discussions of the Kidney Disease Trajectory by Elderly Patients and Nephrologists: A Qualitative Study	2012	American journal of kidney diseases	59 (4)	Schell, J.O.	WRONG OUTCOME (lower GI symptoms prevalences were not assessed)	CCQ (nephrological)
Effects of Hemodialysis on the Symptom Burden of Terminally Ill and Nonterminally Ill End-Stage Renal Disease Patients.	2018	Journal of palliative medicine	22 (3)	Wu, Yi L.	WRONG OUTCOME (lower GI symptoms prevalences were not assessed)	CCQ (nephrological)
End-of-Life Experience of Older Adults Dying of End-Stage Renal Disease: A Comparison With Cancer.	2017	Journal of pain and symptom management	54 (6)	Wachterman, M.W.	WRONG OUTCOME (lower GI symptoms prevalences were not assessed)	CCQ (nephrological)
Evaluating symptom burden in kidney transplant recipients: validation of the revised Edmonton Symptom Assessment System for kidney transplant recipients - a single-center, cross-sectional study.	2020	Transplant international	33 (4)	Dano, S.	WRONG OUTCOME (lower GI symptoms prevalences were not assessed)	CCQ (nephrological)
Groningen frailty indicator in older patients with end-stage renal disease.	2015	Renal failure	37 (9)	Meulendijks, F. G.	WRONG OUTCOME (lower GI symptoms prevalences were not assessed)	CCQ (nephrological)
Health Outcome Priorities of Older Adults with Advanced CKD and Concordance with Their Nephrology Providers' Perceptions.	2018	Journal of the American Society of Nephrology	29 (12)	Ramer, S.J.	WRONG OUTCOME (prevalence not reported)	CCQ (nephrological)
High Symptom Burden and Low Functional Status in the Setting of Multimorbidity	2017	Journal of the American Geriatrics Society	65 (10)	Portz, J.	WRONG POPULATION (lack of subgroup analysis for CKD patients)	CCQ (nephrological)
Impact of Depression on Long-Term Outcome After Renal Transplantation: A Prospective Cohort Study	2012	Transplantation	94 (10)	Zelle, D.M.	WRONG OUTCOME (lower GI symptoms prevalences were not assessed)	CCQ (nephrological)
Increasing Nephrologist Awareness of Symptom Burden in Older Hospitalized End-Stage Renal Disease Patients.	2019	American journal of nephrology	51 (1)	Jawed, A.	WRONG OUTCOME (lower GI symptoms prevalence were not assessed)	CCQ (nephrological)
Individual quality of life in chronic kidney disease: influence of age and dialysis modality.	2009	Clinical journal of the American Society of Nephrology	4 (4)	Abdel-Kader, K.	WRONG OUTCOME (prevalence of GI symptoms was not reported)	CCQ (nephrological)
Kidney symptom questionnaire: Development, content validation and relationship with quality of life	2018	Journal of renal care	44 (3)	Brown, S. A.	WRONG OUTCOME (lower GI symptoms prevalences were not assessed)	CCQ (nephrological)
Living with moderate to severe renal failure from the perspective of patients	2016	BMC nephrology	17 (1)	Schipper, K.	WRONG OUTCOME (lower GI symptoms prevalences were not assessed)	CCQ (nephrological)
Pain, sleep disturbance, and quality of life in patients with chronic kidney disease.	2007	Clinical journal of the American Society of Nephrology	2 (5)	Cohen, S.D.	WRONG OUTCOME (lower GI symptoms prevalences were not assessed)	CCQ (nephrological)

Patient and Clinician Perspectives on Electronic Patient-Reported Outcome Measures in the Management of Advanced CKD: A Qualitative Study.	2019	American journal of kidney diseases	74 (2)	Aiyegbusi, O.L.	WRONG OUTCOME (lower GI symptoms prevalences were not assessed)	CCQ (nephrological)
Physical and Psychological Burden of Chronic Kidney Disease among Older Adults	2010	American journal of nephrology	31 (4)	McClellan, W.	WRONG OUTCOME (lower GI symptoms prevalences were not assessed)	CCQ (nephrological)
Prognostic implications of predialysis patients' symptoms in peritoneal dialysis patients.	2021	Renal failure	43 (1)	Wang, F.-Y.	WRONG OUTCOME (symptoms prevalence based on retrospective analysis of medical documentation; not self-reported)	CCQ (nephrological)
Psychometric properties of the Czech Integrated Palliative Outcome Scale: reliability and content validity analysis	2020	BMC palliative care	19 (1)	Vlckova, K.	WRONG POPULATION (no CKD patients)	CCQ (nephrological)
Quality of life with conservative care compared with assisted peritoneal dialysis and haemodialysis.	2018	Clinical kidney journal	12 (2)	Iyasere, O.	WRONG OUTCOME (prevalence of GI symptoms not reported)	CCQ (nephrological)
Rapid Electronic Capturing of Patient-Reported Outcome Measures in Older Adults With End-Stage Renal Disease: A Feasibility Study:	2020	The American journal of hospice & palliative care	38 (5)	Gabbard, J.	WRONG POPULATION (hemodialysis patients)	CCQ (nephrological)
Serious Illness Treatment Preferences for Older Adults with Advanced CKD.	2019	Journal of the American Society of Nephrology	30 (11)	Baddour, N.A.	WRONG OUTCOME (prevalence not reported)	CCQ (nephrological)
Severe fatigue after kidney transplantation: a highly prevalent, disabling and multifactorial symptom	2013	Transplant international	26 (10)	Goedendorp, M.M.	WRONG OUTCOME (lower GI symptoms prevalences were not assessed)	CCQ (nephrological)
Spanish modified version of the palliative care outcome scale-symptoms renal: cross-cultural adaptation and validation	2016	BMC nephrology	17 (1)	Gutiérrez Sánchez, D.	WRONG OUTCOME (prevalence not reported)	CCQ (nephrological)
Symptom burden in patients with chronic kidney disease not requiring renal replacement therapy.	2017	Clinical kidney journal	10 (6)	Brown, S. A.	WRONG OUTCOME (lower GI symptoms prevalence not assessed)	CCQ (nephrological)
Symptom Burden of Adults with Type 2 Diabetes Across the Disease Course: Diabetes & Aging Study	2012	Journal of general internal medicine	27 (12)	Sudore, R. L.	WRONG OUTCOME (registry-type study)	CCQ (nephrological)
Symptom Clusters From Dialysis to Renal Transplantation: A Five-Year Longitudinal Study.	2015	Journal of pain and symptom management	51 (3)	Amro, A.	WRONG OUTCOME (lower GI symptoms prevalences were not assessed)	CCQ (nephrological)
Symptom experience in non-dialysis-dependent chronic kidney disease: A qualitative descriptive study.	2017	Journal of renal care	43 (4)	Pugh-Clarke, K.	WRONG OUTCOME ("Gastrointestinal disturbances" instead of either constipation or diarrhea)	CCQ (nephrological)
The impact of fatigue on daily activity in people with chronic kidney disease.	2010	Journal of clinical nursing	19 (21)	Bonner, A.	WRONG OUTCOME (lower GI symptoms prevalences were not assessed)	CCQ (nephrological)
Towards a symptom cluster model in chronic kidney disease: A structural equation approach.	2017	Journal of advanced nursing	73 (10)	Almutary, H.	WRONG POPULATION (lack of subgroup analysis for non-dialysis CKD patients)	CCQ (nephrological)
Tracking patients with advanced kidney disease in the last 12 months of life	2018	Journal of renal care	44 (2)	Bonner, A.	WRONG POPULATION (lack of subgroup analysis for non-dialysis CKD patients)	CCQ (nephrological)
Trajectories of illness in stage 5 chronic kidney disease: a longitudinal study of patient symptoms and concerns in the last year of life.	2011	Clinical journal of the American Society of Nephrology	6 (7)	Murtagh, F.E.M.	WRONG OUTCOME (prevalence not reported)	CCQ (nephrological)

Understanding the Experience of stress on initiation of Haemodialysis: A Phenomenological Study	2014	International Journal of Nursing	3 (1)	Tarachand, J.S.	WRONG POPULATION (hemodialysis patients)	CCQ (nephrological)
Unmet palliative care needs among patients with end-stage kidney disease: a national registry study about the last week of life	2017	Journal of pain and symptom management	55 (2)	Axelsson, L.	WRONG OUTCOME (lower GI symptoms prevalences were not assessed)	CCQ (nephrological)
Validation of the IPOS-Renal Symptom Survey in Advanced Kidney Disease: A Cross-sectional Study.	2018	Journal of pain and symptom management	56 (2)	Raj, R.	WRONG POPULATION (lack of subgroup analysis for non-dialysis CKD patients)	CCQ (nephrological)
A High Prevalence of Abnormal Nutrition Parameters Found in Predialysis End-Stage Kidney Disease: Is It a Result of Uremia or Poor Eating Habits?	2014	Journal of renal nutrition	24 (5)	Chan, M.F.	WRONG OUTCOME (lower GI symptoms prevalence not assessed)	CCIS
Assessment of palliative need in patients with chronic kidney disease by the new Three Levels of Need Questionnaire (3LNQ) is not exhaustive.	2014	Danish medical journal	61 (4)	Blindbaek, L.	WRONG OUTCOME (lower GI symptoms prevalence not assessed)	CCIS
Assessment of Quality of Life in Dialysis and Non-Dialysis Chronic Kidney Disease Patients	2017	NA		Islam, M.S.	WRONG POPULATION (unclear settings: probably inpatients)	CCIS
Better health-related quality of life in kidney transplant patients compared to chronic kidney disease patients with similar renal function.	2021	PloS one	16 (10)	Ryu, J.	WRONG OUTCOME (lower GI symptoms prevalence was not assessed)	CCIS
Changes in symptom burden and physical performance with initiation of dialysis in patients with chronic kidney disease.	2014	Hemodialysis international.	19 (1)	Rivara, M.B.	WRONG OUTCOME (lower GI symptoms prevalence was not assessed)	CCIS
Chronic Renal Insufficiency Cohort (CRIC) Study: Baseline Characteristics and Associations with Kidney Function	2009	Clinical journal of the American Society of Nephrology	4 (8)	Lash, J.P.	WRONG OUTCOME (GI symptom prevalence was not reported)	CCIS
CKD in Elderly Patients Managed without Dialysis: Survival, Symptoms, and Quality of Life	2015	Clinical journal of the American Society of Nephrology	10 (2)	Brown, M.	WRONG OUTCOME (GI symptom prevalence was not reported)	CCIS
Clinical events and patient-reported outcome measures during CKD progression: findings from the Chronic Renal Insufficiency Cohort Study.	2020	Nephrology, dialysis, transplantation	36 (9)	Grams, M. E.	WRONG OUTCOME (lower GI symptoms prevalence not assessed)	CCIS
Clinical features and CKD-related quality of life in patients with CKD G3a and CKD G3b in China: results from the Chinese Cohort Study of Chronic Kidney Disease (C-STRIDE).	2017	BMC nephrology	18 (1)	Peng, Z.	WRONG OUTCOME (lower GI symptoms prevalence not assessed)	CCIS
Course of symptoms and health-related quality of life during specialized pre-dialysis care.	2014	PloS one	9 (4)	de Goeij, M. C.M.	WRONG OUTCOME (lower GI symptoms prevalence was not assessed)	CCIS
Development of the Chronic Kidney Disease Symptom Index – Sri Lanka; a symptom assessment instrument for Chronic Kidney Disease patients	2017	Journal of the Postgraduate Institute of Medicine	4 (1)	Sameera J.S.	WRONG OUTCOME (GI symptoms prevalence not reported)	CCIS

Differences In Illness Representations In Patients With Chronic Kidney Disease.	2015	Journal of renal care	41 (3)	Pagels, A.A.	WRONG OUTCOME ("changed bowel habits")	CCIS
Dolor músculo-esquelético en pacientes con enfermedad renal crónica	2016	Nefrologia	36 (4)	Caravaca, F.	WRONG OUTCOME (lower GI symptoms prevalence not assessed)	CCIS
Elements of Palliative Care in the Last 6 Months of Life: Frequency, Predictors, and Timing	2019	Journal of general internal medicine	35 (3)	Ernecoff, N.C.	WRONG OUTCOME (appropriate symptom management instead of symptom prevalence was reported)	CCIS
Event-related distress in kidney disease patients	2011	Nephrology, dialysis, transplantation	27 (1)	Ramer, S.J.	WRONG OUTCOME (GI symptom prevalence was not reported)	CCIS
Exploring Symptoms In Patients Managed Without Dialysis: A Qualitative Research Study	2010	Journal of renal care	36 (1)	Noble, H.	WRONG OUTCOME (prevalence of "Bowel and bladder problems" was reported)	CCIS
Gastointestinal-specific patient-reported outcome instruments differentiate between renal transplant patients with or without GI complications	2005	Transplantation proceedings	37 (2)	Kleinman, L.	WRONG OUTCOME (GSRS subscale rather than symptoms were assessed)	CCIS
Health related quality of life in chronic kidney disease; a descriptive study in a rural Sri Lankan community affected by chronic kidney disease	2020	Health and Quality of Life Outcomes	18 (1)	Sameera S.	WRONG OUTCOME (GI symptoms prevalence not reported)	CCIS
Kidney Disease Symptoms before and after Kidney Transplantation.	2021	Clinical journal of the American Society of Nephrology	16 (7)	Taylor, K.	WRONG OUTCOME (lower GI symptoms prevalence not assessed)	CCIS
Palliative Care Consultation in Advanced Chronic Kidney Disease with Pain.	2018	Journal of palliative medicine	21 (6)	Chan, K.Y.	WRONG OUTCOME (lower GI symptoms prevalence not assessed)	CCIS
Physical Symptom Cluster Subgroups in Chronic Kidney Disease.	2019	Nursing research	69 (2)	Lockwood, M.B.	WRONG OUTCOME (lower GI symptoms prevalence not assessed)	CCIS
Quality of Life and Physical Function in Older Patients on Dialysis: A Comparison of Assisted Peritoneal Dialysis with Hemodialysis	2015	Clinical journal of the American Society of Nephrology	11 (3)	Iyasere, O.	WRONG POPULATION (only dialysis-dependent patients)	CCIS
Quality of Life and Survival in Patients with Advanced Kidney Failure Managed Conservatively or by Dialysis	2012	Clinical journal of the American Society of Nephrology	7 (12)	Da Silva-Gane, M.	WRONG OUTCOME (GI symptoms prevalence not assessed)	CCIS
Quality of life in Chronic Kidney Disease (CKD): A cross-sectional analysis in the Renal Research Institute-CKD study	2005	American journal of kidney diseases	45 (4)	Perlman, R.L.	WRONG OUTCOME (GI symptoms not assessed)	CCIS
Quality of Life in Pre-dialysis patients with Chronic Kidney Disease at Glomerular Filtration Rates	2013	Journal of Korean Biological Nursing Science	15 (2)	Kim, H.W.	WRONG OUTCOME (lower GI symptoms prevalence not assessed)	CCIS
Quality of life with conservative care compared with assisted peritoneal dialysis and haemodialysis.	2018	Clinical kidney journal	12 (2)	Iyasere, O.	WRONG OUTCOME (GI symptoms prevalence not reported)	CCIS
Relationships Between Illness Perceptions, Coping and Psychological Morbidity in Kidney Transplants Patients.	2016	The American journal of the medical sciences	351 (3)	Knowles, S.R.	WRONG OUTCOME (GI symptoms prevalence not assessed)	CCIS

Relevance of heat stress and dehydration to chronic kidney disease (CKDu) in Sri Lanka	2019	Preventive medicine reports	15	Jayasekara, K.B.	WRONG POPULATION (lack of subgroup analysis for CKD subgroup)	CCIS
Screening for constipation in palliative care patients.	2009	Journal of palliative medicine	12 (10)	Noguera, A.	WRONG POPULATION (lack of analysis for CKD patients)	CCIS
Self-rated health, quality of life and appetite as predictors of initiation of dialysis and mortality in patients with chronic kidney disease stages 4-5: a prospective cohort study.	2018	BMC research notes	11 (1)	Grove, B.E.	WRONG OUTCOME (lower GI symptoms prevalence not assessed)	CCIS
Sleep disorders, depressive symptoms and health-related quality of life--a cross-sectional comparison between kidney transplant recipients and waitlisted patients on maintenance dialysis	2010	Nephrology, dialysis, transplantation	26 (3)	Kovacs, A.	WRONG OUTCOME (lower GI symptoms prevalence not assessed)	CCIS
Study Of Clinical Profile Of Chronic Kidney Disease In Non-Diabetic Patients	2021	International Journal of Advances in Medicine	8 (8)	Kumar, U R.	WRONG OUTCOME (lower GI symptoms prevalence not assessed)	CCIS
The SF36 as an outcome measure of services for end stage renal failure.	1998	Quality in health care	7 (4)	Wight, J	WRONG OUTCOME (lower GI symptoms prevalence not assessed)	CCIS
The suffering of advanced chronic renal patients and their relationship with symptoms in loja, ecuador	2021	International journal of environmental research and public health	18 (10)	Bonilla-Sierra, P.	WRONG OUTCOME (lower GI symptoms not assessed)	CCIS
The Symptoms Prevalence, Medical Interventions, and Health Care Service Needs for Patients With End-Stage Renal Disease in a Renal Palliative Care Program	2015	The American journal of hospice & palliative care	33 (10)	Kwok, A.O.	WRONG OUTCOME (prevalence of "Bowel problem" and "Distended abdomen" was reported)	CCIS
Trajectory of Quality of Life for Poor Prognosis Stage 5D Chronic Kidney Disease with and without Dialysis	2013	American journal of nephrology	37 (3)	Seow, Y.-Y.	WRONG OUTCOME (GI symptoms prevalence not assessed)	CCIS
Why did I start dialysis? A qualitative study on views and expectations from an elderly cohort of patients with end-stage renal failure starting haemodialysis in the United Kingdom	2011	International urology and nephrology	44 (1)	Stringer, S.	WRONG OUTCOME (lower GI symptoms prevalence not assessed)	CCIS
Wie unterscheiden sich Bedürfnisse und Versorgung in der SAPV in Abhängigkeit von der Grunderkrankung? Auswertungen aus dem Nationalen Hospiz- und Palliativregister	2020	Z Palliativmed	21 (4)	Kaiser, F.	WRONG POPULATION (lack of analysis for CKD patients)	CCIS

*** Abbreviations: DS:** Databases screening; **CCIS:** Citation chasing of included studies; **CCQ:** Citation chasing of questionnaires.

Table S6. Exclusion criteria in the included studies.

First author, reference	Exclusion criteria
Gordon, [21]	Not reported
Yapa, [16]	"Those with medically determined cognitive impairment and/or acute illness (such as peritonitis, myocardial infarction or respiratory infection)"
Ariffin, [22]	"patients less than 18 years of age, with a strong history of non-compliance with medication and treatment, with recent hospitalization (within 3 months) and have evidence of recent active infections (including bacterial and viral)"
Ramos, [17]	"individuals with diabetes mellitus, chronic liver disease, autoimmune disease (i.e., systemic lupus erythematosus, rheumatoid arthritis), congestive heart failure (stages 3/4), human immunodeficiency virus, current malignancy, bowel diseases (i.e., inflammatory bowel diseases, celiac disease), and/or cognitive limitations; who are current smokers; and who are using medications including phosphate binders, immunosuppressants, anti-inflammatories, antibiotics, laxatives, prebiotics, and/or probiotics 3 months preceding the baseline."
Trimingham, [23]	"patients who were in hospital, age <18 years, non-English speaking patients requiring an interpreter, those unable to complete the written or verbal questionnaire (i.e. cognitive impairment) and those who declined to participate"
Sanya, [24]	Diabetic mellitus, coronary heart disease, congestive heart disease, usage of drugs that can influence the cardiovascular or autonomic system
Saini, [25]	"patients aged <18 years and patients who were unclear about their diagnosis and its implications", cancer diagnosis, known reversible kidney disease, renal replacement therapy, eGFR > 16.5
Ohkuma, [26]	"(1) they had drug-induced diabetes or were undergoing steroid treatment; (2) they were being administered renal replacement therapy; (3) they had serious diseases other than diabetes,such as advanced malignancies or decompensated liver cirrhosis; or (4) they were unable to regularly visit a hospital or clinic"; (5) "past history of colon cancer"; (6) "type 1 diabetes"
Ruszkowski, [18,27]	"receiving currently or in the past dialysis; kidney transplantation; cognitive deficits and visual impairment that unable of answering the questionnaire; having a serious illness in an acute treatment phase"
Quintal-Medina, [28]	"sepsis, pericarditis, pleurisy, and uremic encephalopathy"
Meade, [29]	"under 18 years old, from a non-English speaking background requiring an interpreter, were unable to complete the questionnaires or receiving temporary dialysis"
Wizemann, [30]	Not reported
Zhang, [19]	"institutionalized (e.g., prisoner, nursing home or skilled nursing facility resident); unable or unwilling to give consent; unlikely or unable to participate in required study procedures; New York Heart Association class III or IV heart failure (baseline); known cirrhosis; known HIV infection and/or AIDS; pregnant women; previously received dialysis for ≥ 1 mo; previous organ or bone marrow transplant; received immunosuppressive or other immunotherapy for primary renal disease or systemic vasculitis within the past 6 mo; previous chemotherapy or alkylating agents for systemic cancer other than non-melanoma skin cancer within 2 yr; previous diagnosis of multiple myeloma or renal carcinoma; polycystic kidney disease; current participation in interventional clinical trial or in a research study" [31]
Gryp, [20,32]	"age < 18 years, active infection (C-reactive protein > 20 mg/L), active malignancy, cardiovascular event in the past three months, immunosuppressive therapy, inflammatory bowel disease, obesity (BMI > 35 kg/m^2), pregnancy, transplantation, and/or use of non-steroidal anti-inflammatory drugs within the past month"
Miskulin and the HALT-PKD studies investigators, [33,34]	Main exclusion criteria: kidney vascular disease; systemic diseases with kidney involvement; UACR ≥0.5 or 1.0 g/g; diabetes; currently pregnant or intention of becoming pregnant; increased serum potassium level; history of angioneurotic edema, other absolute contraindication for ACEi/ARB or intolerable cough

	associated with ACEi; systemic diseases necessitating NSAIDs, immunosupressant, or immunomodulatory medications; life expectancy <2 yr; "congenital absence of a kidney or history of a total nephrectomy"
Windahl and the EQUAL study investigators, [35–38]	< 65 years, eGFR ≥ 20 mL/min/1.73 m^2, dialysis
Ducharlet, [39]	Patients with eGFR < 15 ml/min/1.73 m^2 were excluded from the analysis
Grove, [15]	“patients reporting change in health and patients who have been in contact with the clinic”
Onodugo, [40]	"severe cardiac disease, cancer, diabetes, collagen and demyelinating diseases, left ventricular systolic dysfunction, or a history of stroke"
Allawi, [41]	Patients who (1) suffer from chronic kidney disease for less than 6 months, (2) are on dialysis, (3) have diseases that might be associated with autonomic neuropathy (diabetes mellitus, heart failure, stroke), (4) use drugs that affect the autonomic nervous system (beta-blockers, tricyclic antidepressants or purgatives), (5) are in the uremic syndrome, i.e. present with persistent nausea & vomiting, encephalopathy or acidotic breathing or bedridden, (5) have GFR > 15 mL/min per 1.73 m^2.
Lee A, [42]	"patients under the care of a nephrologist with an eGFR ≤15 ml/min who had chosen not to dialyse (conservative management); (...) kidney transplant recipients; patients with a colostomy or ileostomy and those with inadequate English language skills to complete a written questionnaire."
Dawson, [14]	Not reported
Abeywickrama, [11]	"Patients with psychiatric/cognitive disorders or language barriers and patients who refused to participate in the study"
Lee SJ, [12]	Not reported
Yong, [43]	"Patients with cognitive impairment or known psychiatric illness"
Senanayake, [13]	"patients with previous renal transplantation, critically ill patients and patients who were unable to provide rational information for any reason (e.g.; mental retardation)" [44]
Abdel-Kader, [45]	"age <18 yr or >90 yr, not residing at home, active malignancy, active infection (pneumonia), active coronary artery disease (e.g., unstable angina, myocardial infarction) within the last 6 mo, advanced cirrhosis, advanced dementia, active alcohol abuse, active treatment for sleep apnea, refractory psychiatric disease, or an unsafe home environment"
Wan Zukiman, [46]	"pregnancy; presence of any type of acute psychiatric disorder that warrant hospitalization, acute medical illnesses, or malignancy; lack of capacity to give informed consent; inability to communicate fluently in Malay or English language; or illiteracy"
Gutiérrez Sánchez, [47–50]	"Patients with cognitive impairment and those under 18 years of age"
Murtagh, [51,52]	"Patients who lacked capacity to consent to research participation (as judged by clinician assessment)"
Turkmen, [53]	„diabetic kidney disease or other kidney disease including glomerulonephritis, lupus nephritis, systemic vasculitis proven by kidney biopsy”, age > 70
Brennan, [54]	Not reported (patients with age < 18 years old, lack of decision for a conservative management of CKD G5 were not included)
Murphy, [55]	Not reported
Taira, [56]	Not reported
Purtell & Sowa, [57,58]	Not reported
De Miguel, [59]	Not reported
Almutary, [60,61]	"cognitive impairment that would preclude voluntary, informed consent, and those with critical conditions"

Table S7. Risk of bias in included studies.

Title [a], reference	Q1	Q2	Q3	Q4	Q5	Q6	Q7	Q8	Q9
Abnormal intestinal bile acid distribution in azotaemic man: a possible role in the pathogenesis of uraemic diarrhea [21]	No	Unclear	No	No	Unclear	Unclear	Unclear	Not applicable	Unclear
Alterations in symptoms and health-related quality of life as kidney function deteriorates: A cross-sectional study [16]	Yes	No	Yes	Unclear	Unclear	Yes	Yes	Not applicable	Yes
Appetite and gastrointestinal symptoms in end stage renal disease patients [22]	Unclear	Unclear	No	No	Unclear	No	Unclear	Not applicable	Unclear
Bowel Habits and the Association With Uremic Toxins in Non-Dialysis-Dependent Chronic Kidney Disease Patients [17]	No	Unclear	No	Yes	Unclear	Yes	Yes	Yes	Unclear
Bowel health in chronic kidney disease: Patient perceptions differ from clinical definitions [23]	Unclear	No	No	No	Unclear	Yes	Yes	Not applicable	Unclear
Cardiovascular autonomic neuropathy in non-diabetic Nigerian patients with chronic renal failure [24]	No	No	No	No	Unclear	Unclear	Yes	No	Unclear
Comparative pilot study of symptoms and quality of life in cancer patients and patients with end stage renal disease [25]	Unclear	Unclear	No	No	Unclear	Yes	Unclear	Not applicable	Unclear
Constipation and diabetic kidney disease: The Fukuoka Diabetes Registry [26]	Yes	Yes	Yes	Unclear	Unclear	Yes	Yes	Unclear	Unclear
Constipation and the Quality of Life in Conservatively Treated Chronic Kidney Disease Patients: A Cross-sectional Study [18,27]	Yes	Yes	No	Yes	Unclear	Yes	Not applicable	Yes	Yes
Factors associated with residual symptom burden in patients with peritoneal dialysis: a cohort study [28]	No	Unclear	No	Yes	Unclear	Yes	Unclear	Not applicable	Unclear
Gastrointestinal symptom burden and dietary intake in patients with chronic kidney disease [29]	Yes	No	No	Yes	Yes	Yes	Yes	Unclear	Yes

Gastrointestinal symptoms in patients suffering from chronic uremia [30]	Unclear	Unclear	No	No	Unclear	No	Unclear	Not applicable	Unclear
Gastrointestinal symptoms, inflammation and hypoalbuminemia in chronic kidney disease patients: a cross-sectional study [19]	Unclear	Unclear	Yes	Unclear	Unclear	Unclear	Yes	Not applicable	Yes
Gut microbiota generation of protein-bound uremic toxins and related metabolites is not altered at different stages of chronic kidney disease [20,32]	Unclear	Unclear	No	Yes	Unclear	Yes	Yes	No	Unclear
Health-Related Quality of Life in Patients With Autosomal Dominant Polycystic Kidney Disease and CKD Stages 1–4: A Cross-sectional Study [33,34]	No	Yes	Yes	Yes	Unclear	Unclear	Yes	Yes	Yes
Patient-Reported Measures and Lifestyle Are Associated With Deterioration in Nutritional Status in CKD Stage 4-5: The EQUAL Cohort Study [35–38]	Unclear	Unclear	Yes	Yes	Unclear	Yes	Yes	Yes	Unclear
Patient-reported outcome measures and their utility in the management of patients with advanced chronic kidney disease [39]	Unclear	Unclear	No	No	Unclear	Yes	Unclear	Yes	No
Patient-reported outcome measures for clinical decision-making in outpatient follow-up: validity and reliability of a renal disease questionnaire [15]	Unclear	Yes	No	No	Unclear	Yes	Yes	Not applicable	No
Predictors of autonomic dysfunction among pre-dialysis chronic kidney disease patients in Nigeria [40]	No	Unclear	No	No	Unclear	Unclear	Unclear	No	Unclear
Prediction of autonomic neuropathy in chronic kidney disease (stage 5) Iraqi patients (case control study) [41]	No	Unclear	No	No	Unclear	Unclear	Unclear	No	Unclear
Prevalence of constipation in patients with advanced kidney disease [42]	Yes	Yes	No	No	Unclear	No	Yes	Yes	Yes

Prevalence of Taste Changes and Association with Other Nutrition-Related Symptoms in End-Stage Kidney Disease Patients [14]	Unclear	Unclear	No	No	Unclear	Yes	Unclear	Yes	Unclear
Quality of Life and Symptom Burden among Chronic Kidney Disease of Uncertain Etiology (CKDu) Patients in Girandurukotte, Sri Lanka [11]	No	Yes	No	No	Unclear	Yes	Unclear	Not applicable	Unclear
Relationship between symptom clusters and quality of life in patients at stages 2 to 4 chronic kidney disease in Korea [12]	Unclear	Unclear	No	No	Unclear	Yes	Unclear	Yes	Unclear
Symptom burden and quality of life in end-stage renal disease: a study of 179 patients on dialysis and palliative care [43]	Yes	Yes	No	No	Unclear	No	Yes	Not applicable	Unclear
Symptom burden in chronic kidney disease; a population based cross sectional study [13]	Yes	Yes	Yes	No	Unclear	Yes	Yes	Not applicable	Yes
Symptom burden, depression, and quality of life in chronic and end-stage kidney disease [45]	Unclear	Unclear	No	No	Unclear	Yes	Unclear	Unclear	Unclear
Symptom Prevalence and the Negative Emotional States in End-Stage Renal Disease Patients with or without Renal Replacement Therapy: A Cross-Sectional Analysis [46]	Yes	Yes	No	Yes	Yes	Yes	Yes	Not applicable	Yes
Symptomatic profile of patients with Chronic Kidney Disease Stage 4 and 5 [47–50]	Yes	Unclear	No	No	Unclear	Yes	No	Not applicable	Unclear
Symptoms in advanced renal disease: a cross-sectional survey of symptom prevalence in stage 5 chronic kidney disease managed without dialysis [51,52]	Unclear	Yes	No	Yes	Yes	Yes	Unclear	Yes	Yes
The Prevalence of Fabry Disease in Patients with Chronic Kidney Disease in Turkey: The TURKFAB Study [53]	No	Yes	No	No	Not applicable	No	Unclear	Not applicable	Unclear
The symptoms of patients with CKD stage 5 managed without dialysis [54]	Yes	Unclear	No	Yes	Unclear	Yes	Yes	Not applicable	Unclear

Understanding symptoms in patients with advanced chronic kidney disease managed without dialysis: use of a short patient-completed assessment tool [55]	Unclear	Unclear	No	Unclear	Unclear	Yes	Yes	Not applicable	Unclear
Urinary concentrations of neonicotinoid insecticides were related to renal tubular dysfunction and neuropsychological complaints in Dry-zone of Sri Lanka [56]	No	Unclear	No	No	Unclear	Unclear	Yes	Not applicable	Unclear
The Kidney Supportive Care programme: characteristics of patients referred to a new model of care [57,58]	Unclear	Yes	No	No	Yes	Yes	Yes	Not applicable	Not applicable
What are the last months of life like for advanced chronic renal failure patients who are not considered candidates for treatment with haemodialysis or peritoneal dialysis [59]	Yes	Yes	No	Yes	Yes	Unclear	Unclear	Not applicable	Not applicable
Which patients with chronic kidney disease have the greatest symptom burden? A comparative study of advanced CKD stage and dialysis modality [60]	Yes	No	No	Unclear	Unclear	Yes	Yes	Not applicable	Unclear

[a] If a study had more than one report, the title of the oldest one or most important article has been chosen.
Questions according to Joanna Briggs Institute (JBI) Critical Appraisal Checklist for Prevalence Studies [62]. Q1: Was the sample frame appropriate to address the target population? Q2: Were study participants sampled in an appropriate way? Q3: Was the sample size adequate? Q4: Were the study subjects and the setting described in detail? Q5: Was the data analysis conducted with sufficient coverage of the identified sample? Q6: Were valid methods used for the identification of the condition? Q7: Was the condition measured in a standard, reliable way for all participants? Q8: Was there appropriate statistical analysis? Q9: Was the response rate adequate, and if not, was the low response rate managed appropriately?

Table S8. Severity of self-reported constipation

	Total (N analyzed participants)	Mild (Slight)	Moderate	Severe	Overwhelming (very severe)
**CKD G1-2**					
Lee SJ (G1-2)	7	4	2	1	0
Prevalence (95% CI) [a]		57.14% (28.6-91.5)	28.57% (0-62.9)	14.29% (0-48.6)	0% (0-34.3)
**CKD G3**					
Dawson (G3)	1	1	0	0	0
Lee SJ (G3)	36	14	11	6	5
Yapa (G3)	71	19	42	10	0
Prevalence (95% CI) [b]		37.60% (10.4-69.1)	40.92% (12.8-72.2)	15.84% (0-41.2)	5.65% (0-23.9)
**CKD G4-5**					
Lee SJ (G4-5)	22	9	8	3	2
Dawson (G4-5)	28	15	8	5	0
Murphy	23	9	11	3	0
Sowa	26	16	7	2	1
Yapa (G4-5)	190	73	95	22	0
Sánchez	22	8	7	6	1
Brennan	18	11	3	3	1
Quintal-Medina	31	7	8	12	4
Wan Zukiman	32	26		6	
Ducharlet	15	10		5	
Prevalence (95% CI) [c]		43.81% (32.8-54.4)	34.43% (24.2-44.9)	18.16% (10.3-27.2)	3.60% (0.4-9.0)

[a] Only one study provided data, thus meta-analysis could not be performed. Confidence intervals for multinomial proportions were calculated according to the method of Sison and Glaz. [b] Meta-analysis was conducted using a random-effects model with Freeman–Tukey double arcsine transformation of proportions ($\tau = 0.182$; $I^2 = 82\%$). [c] Data from both Wan Zukiman *et al*. and Ducharlet *et al*. was excluded from the meta-analysis because of the non-comparable format of that data. Meta-analysis of the remaining studies was conducted using random-effects model with Freeman–Tukey double arcsine transformation of proportions ($\tau = 0.064$; $I^2 = 70\%$).

Table S9. Subgroup analysis for self-reported constipation prevalence in chronic kidney disease (CKD) G4-5

Subgroup	N	Prevalence		τ^2	*P* value
		[%]	95% CI		
**WHO Region**					0.02
Western Pacific	9	41.36	32.94–50.32	0.21	
European	8	31.44	26.78–36.50	0.05	
American	2	38.85	31.55–46.69	0	
Eastern Mediterranean	2	94.60	0.61–100.0	22.48	
South-East Asian	1	42.89	38.35–47.55	-	
**Study period**					0.05
After 2010	15	43.71	32.26–55.88	0.83	
2000–2010	6	32.59	27.11–38.59	0.03	
Before 2000	1	15.00	4.92–37.58	-	
**Average age** [a]					0.49
Lower tercile (< 64 y)	7	51.17	24.90–76.81	2.20	
Medium tercile	6	39.12	32.48–46.18	0.03	
Upper tercile (> 75 y)	7	35.40	28.49–42.97	0.12	
**Sex** [b]					0.58
More males	12	39.00	25.64–54.25	1.08	
More females	8	43.74	36.52–51.24	0.09	

[a] Two studies (Wizemann *et al*. [30] and Ariffin *et al*. [22]) were excluded from the analysis because they did not provide data on the age of participants. Also, average age was not a significant predictor of the prevalence in the meta-regression model (*P* = 0.38).

[b] Two studies (Wizemann *et al*. [30] and Ariffin *et al*. [22]) were excluded from the analysis because they did not provide data on the number of males in CKD G4-5 groups. Also, the percentage of males was not a significant predictor of the prevalence in the meta-regression model (*P* = 0.46).

Table S10. Severity of self-reported constipation (alternative version)

	Total (N analyzed participants)	Not at all	A little (a little bit)	Somewhat	Quite a bit	Very much
**CKD G3**						
Grove	141	111	21	-	8	1
Prevalence (95% CI) [a]		78.7% (73.0-85.7)	14.9% (9.2-21.9)		5.7% (0-12.7)	0.7% (0-7.7)
**CKD G4-5**						
EQUAL study	361	22	144	105	70	20
Murtagh	64	41	8	10	5	
Grove	90	62	23	-	5	0
Prevalence (95% CI) [b]		32.5% (0-100)	26.9% (0-100)	23.7% (0-100)	16.9% (0-100)	

[a] Only one study provided data, thus meta-analysis could not be performed. Confidence intervals for multinomial proportions were calculated according to the method of Sison and Glaz. [b] Data from both Grove *et al*. were excluded from the meta-analysis because of the non-comparable format of data (lack of "somewhat" category). Meta-analysis of the remaining studies was conducted using a random-effects model with Freeman–Tukey double arcsine transformation of proportions (τ = 0.899; I^2 = 99%).

Table S11. Relationships between self-reported constipation and health-related quality of life (HRQoL), clinical data, or laboratory tests results

Author, reference (outcome*)	HRQoL	Clinical data	Laboratory data
Lee SJ [12] (P)	Principal component analysis (varimax rotation) revealed that both constipation and diarrhea clustered together with the 'difficulty sleeping' item into the "neurological and bowel problem" symptom cluster. The score of this cluster correlated negatively with summary metrics of health-related quality of life scale SF36v2, i.e. with both physical (r = −0.29 *P*<0.001) and mental (r = −0.31 *P*<0.001) component summaries.	Patients with cardiovascular diseases had a higher score (more severe) of the "neurological and bowel problem" factor (*P* = 0.03), i.e. diarrhea, constipation, and difficulty sleeping. "There were no relationships between the severity of the symptom clusters and gender, age, educational background, having spouses, or current occupations" (*P* values not reported).	There were no significant correlations between score of "neurological and bowel problem" symptom cluster and serum creatinine level (r = 0.05), eGFR (r = −0.11), blood urea nitrogen level (r = −0.08), or hemoglobin level (r = −0.07) (*P* values not reported).
EQUAL study investigators [35–38] (P, S)	Presence of constipation was associated with lower HRQoL measured with the RAND-36: both physical (coefficient: −7.5; SE 1.57; N = 994; *P* <0.001) and mental (coefficient: −8.0; SE 1.49; N = 1086; *P* <0.001) component score. Similarly, constipation severity correlated with both physical (r = −0.19, N = 1001, *P* < 0.001) and mental (r = −0.20, N = 1092, *P* < 0.001) component score. [Information received from the Authors]	There were no differences in age-adjusted prevalence of constipation between women and men of ≥65 years of age (*P* = 0.27). Constipation was one of the symptoms whose prevalence increased the most over the 1-year follow-up period (ca. +8.6%). In the multivariable models, constipation was associated with a decline in nutritional status evaluated with the Subjective Global Assessment tool (SGA; "at least 1 point decline in SGA at any visit during the first 12 months of follow-up"; OR 1.41, 95% CI 1.20-1.67).	NR

Yapa [16] (P, S)	Constipation severity did significantly correlate with summary measures of SF-36v2 questionnaire: both Physical (PCS; r=0.17 (*P* < 0.001) and Mental (MCS; r=0.15, *P* < 0.001) Component Summary. [Information from correspondence with authors]	Prevalence of constipation was significantly associated with the stage of CKD (*P* < 0.05); the increasing trend was observed from G3b stage (31.7%) through G4 (39.6%) up to G5 stage (46.2%). Otherwise, severity was not significantly associated with stage of CKD (*P* value not reported). Patients with CKD G5 did not significantly differ from dialysis patients in the case of constipation prevalence (46% vs G5D: 43%; *P* = 0.50).	NR
Dawson [14] (P)	In the conservatively managed patients, there was a significant association between taste disturbances and constipation prevalence (*P* = 0.005).	NR	NR
Sanya [24] (P)	NR	CKD patients with autonomic neuropathy reported constipation more frequently than CKD patients without autonomic neuropathy (59% vs 33%).	NR
Onodugo [40] (P)	NR	In the multivariable regression model, constipation was not associated with autonomic dysfunction (*P* = 0.39).	NR
Ducharlet [39] (P)	NR	Patients with CKD G4 did not differ from dialysis patients in the case of constipation prevalence (48% vs 56%; *P* = 0.45).	NR
Abdel-Kader [45] (P, S)	NR	Neither median severity of constipation (*P* = 0.7) nor prevalence of constipation (G4-5: 33% vs G5D: 26%, *P* = 0.3) differed between non-dialysis and dialysis patients.	NR
Ariffin [22] (P)	NR	Non-dialysis patients with CKD G5 did not significantly differ in the self-reported constipation prevalence from healthy controls (22% vs 13%, *P* = 0.16) or dialysis-dependent patients (22% vs 30%, *P* = 0.27).	NR
Lee A [42] (P)	NR	Non-dialysis patients with CKD G5 did not significantly differ in the self-reported constipation prevalence from dialysis-dependent patients (62% vs G5D: 42%, *P* = 0.09).	NR
Yong [43] (P)	NR	Non-dialysis patients with CKD G5 did not significantly differ in the self-reported constipation prevalence from dialysis-dependent patients (36% vs G5D: 28%, *P* = 0.31).	NR
Wan Zukiman [46] (P)	NR	Non-dialysis patients with CKD G5 did not significantly differ in the self-reported constipation prevalence from dialysis-dependent patients (32% vs G5D: 21%, *P* = 0.08).	NR
Taira [56] (P)	NR	Non-dialysis patients with CKD of unknown etiology (eGFR not reported) had higher prevalence of self-reported constipation than their healthy family members and neighbors (13.3% vs 1.3%, *P* = 0.019).	NR
Gutiérrez Sánchez [47,49] (P, S)	NR	Factor analysis (maximum likelihood extraction, oblimin rotation; both non-dialysis and dialysis-dependent CKD G5), constipation is not clustered together with other gastrointestinal (diarrhea, nausea, vomiting) or "neuropsychological" (weakness, mouth problems, poor mobility, difficulty sleeping, feeling anxious, and feeling depressed)	NR

		symptoms. Non-dialysis patients with CKD G4-5 had higher prevalence of self-reported constipation than dialysis-dependent patients (41% vs G5D: 25%, *P* = 0.015).	
Almutary [61] (P, S)	NR	Factor analysis (principal axis factoring, oblique rotation; CKD patients including dialysis-dependent) revealed that gastrointestinal symptom cluster includes nausea and vomiting as core symptoms across all dimensions. Constipation is not related to this cluster at any dimension. Non-dialysis patients with CKD G5 had lower prevalence of self-reported constipation than dialysis-dependent patients (23.7% vs G5D: 45%, *P* = 0.012).	NR
Murtagh [51,52] (P)	NR	In the subgroup of patients who died, the prevalence of constipation within a month before death was 1.87 times higher than in the whole baseline group (65% [95% CI: 50-78%]).	NR

* Outcome that was tested for association with other data. NR: not reported; P: prevalence; S: severity.

Table S12. Relationships between functional constipation and HRQoL, clinical data, or laboratory tests results.

Authors	HRQoL	Laboratory data	Clinical data
Ruszkowski, [18,27]	Functional constipation (FC) was significantly associated with worse assessment of some domains of HRQoL: role limitations due to physical health problems, bodily pain, and vitality. CKD patients with FC were more likely to have impaired sleep quality in comparison to patients without FC (PR 2.71, 95% CI 1.21-6.07, *P* = 0.02). The associations maintain to be significant after adjustment for key clinical data. Specifically, FC was significantly associated with severity of insomnia, but not with excessive daytime sleepiness.	When all patients were divided into 3 groups based of eGFR value (≤ 32, 33-43, ≥44 ml/min/1.73 m^2), patients with the lowest eGFR had more frequently FC than these with the highest eGFR (adjusted PR 2.85, 95% CI 1.12 to 7.28).	CKD patients who were treated with paracetamol were more likely to have FC than patients not receiving this drug (adjusted PR 2.67, 95% CI 1.07-6.64). Likewise, taking non-steroidal anti-inflammatory drugs was independently associated with lower PR of FC (adjusted PR 0.34, 95% CI 0.11 to 1.00). Gender, age, body mass index were not significantly associated with altered prevalence of FC.
Ramos, [17]	NR	Authors noticed that "using the Rome III criteria, a trend for higher levels of *p*-cresyl sulfate was observed in constipated participants when compared with non-constipated participants"; however, there were no significant associations (*P* values ranged from 0.06 to 0.16).	"No differences were found in gender, age, body mass index, and dietary parameters between constipated and non-constipated groups assessed by both Rome III criteria" (*P* values not reported).
Lee A [42] (P)	NR	NR	Non-dialysis patients with CKD G5 did not significantly differ in the FC prevalence from dialysis-dependent patients (5% vs 13%, *P* = 0.26).

NR: not reported

Table S13. Severity of self-reported diarrhea

	Total (N analyzed participants)	Mild (including very mild, slight)	Moderate	Severe	Overwhelming (very severe)
**CKD G1-2**					
Lee SJ (G1-2)	6	4	1	0	1
Senanayake (G1-2)	9	3	2	2	2
Prevalence (95% CI) [a]		46.8% (15.5-80.9)	20.0% (0-53.9)	13.2% (0-40.2)	20.0% (0-53.9)
**CKD G3**					
Abeywickrama (G3)	9	8	1	0	0
Dawson (G3)	1	0	0	1	0
Lee SJ (G3)	12	8	2	1	1
Senanayake (G3)	11	8	2	1	0
Yapa (G3)	19	9	10	0	0
Prevalence (95% CI) [b]		63.0% (43.7-82.4)	25.2% (9.8-45.4)	8.0% (0-20.9)	3.8% (0-13.6)
**CKD G4-5**					
Abeywickrama (G4-5)	17	15	2	0	0
Brennan	9	3	4	1	1
Dawson (G4-5)	15	7	5	3	0
Lee SJ (G4-5)	5	3	2	0	0
Murphy	6	5	1	0	0
Sowa	15	5	5	5	0
Yapa (G4-5)	66	28	35	3	0
Senanayake (G4-5)	37	10	19	4	4
Sánchez	9	4	4	1	0
Quintal-Medina	26	7	4	11	4
Wan Zukiman	20	18 (Mild + Moderate)		2 (Severe + Overwhelming)	
Ducharlet	13	12 (Mild + Moderate)		1 (Severe + Overwhelming)	
Prevalence (95% CI) [c]		47.1% (33.3-59.7)	35.6% (23.0-48.2)	13.5% (5.4-23.6)	3.9% (0.1-10.9)

[a] Meta-analysis was conducted using a random-effects model with Freeman–Tukey double arcsine transformation of proportions (τ = 0.085; I^2 = 40%). [b] Meta-analysis was conducted using a random-effects model with Freeman–Tukey double arcsine transformation of proportions (τ = 0.096; I^2 = 49%). [c] Data from both Wan Zukiman *et al*. and Ducharlet *et al*. was excluded from the meta-analysis because of the non-comparable format of data. Meta-analysis of the remaining studies was conducted using a random-effects model with Freeman–Tukey double arcsine transformation of proportions (τ = 0.111; I^2 = 68%).

Table S14. Subgroup analysis for self-reported diarrhea prevalence in CKD G4-5

Subgroup	N	Prevalence		τ^2	*P* value
		[%]	95% CI		
**WHO Region**					< 0.001
Western Pacific	7	20.42	13.97–28.85	0.25	
European	7	18.20	11.13–28.35	0.44	
South-East Asian	3	11.79	5.40–23.83	0.52	
American	2	30.64	23.11–39.38	0.02	
Eastern Mediterranean	1	6.54	3.15–13.09	-	
**Study period**					0.63
After 2010	15	18.54	13.18–25.43	0.55	
2000–2010	4	16.35	8.88–28.17	0.32	
Before 2000	1	10.00	2.51–32.38	-	
**Average age** [a]					0.28
Lower tercile (< 62 y)	6	14.86	8.02–25.88	0.69	
Medium tercile	6	25.51	16.74–36.85	0.31	
Upper tercile (> 75 y)	6	18.40	12.39–26.45	0.23	
**Sex** [b]					0.52
More males	12	20.09	13.54–28.75	0.61	
More females	6	0.1675	11.07–24.54	0.21	

[a] Two studies (Wizemann *et al*. [30] and Ariffin *et al*. [22]) were excluded from the analysis because they did not provide data on the age of participants. Also, average age was not a significant predictor of the prevalence in the meta-regression model (*P* = 0.76).

[b] Two studies (Wizemann *et al*. [30] and Ariffin *et al*. [22]) were excluded from the analysis because they did not provide data on the number of males in CKD G4-5 groups. Also, the percentage of males was not a significant predictor of the prevalence in the meta-regression model (*P* = 0.45).

Table S15. Relationships between self-reported diarrhea and HRQoL, clinical data, or laboratory test results

Authors, reference (outcome*)	HRQoL	Clinical data	Laboratory data
Lee SJ [12] (P, S)	Principal component analysis (varimax rotation) revealed that both constipation and diarrhea clustered together with the 'difficulty sleeping' item into the "neurological and bowel problem" symptom cluster. The score of this cluster correlated negatively with summary metrics of health-related quality of life scale SF36v2, i.e. with both physical ($r = -0.289$, $P < 0.001$) and mental ($r = -0.308$, $P < 0.001$) component summaries.	Patients with cardiovascular diseases had a higher score (more severe) of the "neurological and bowel problem" factor ($P = 0.026$), i.e. diarrhea, constipation, and difficulty sleeping. "There were no relationships between the severity of the symptom clusters and gender, age, educational background, having spouses, or current occupations" (*P* values not reported).	There were no significant correlations between score of "neurological and bowel problem" symptom cluster and serum creatinine level ($r = 0.051$), eGFR ($r = -0.109$), blood urea nitrogen level ($r = -0.079$), or hemoglobin level ($r = -0.069$) (*P* values not reported).
EQUAL study investigators [35–38] (P, S)	Presence of diarrhea was associated with lower HRQoL measured with the RAND-36: both physical (coefficient: −6.6; SE 1.63; N = 996; $P < .0001$) and mental component score (coefficient: −9.2; SE 1.54; N = 1084; $P < .0001$). Also, the severity of diarrhea correlated negatively with both physical (Pearson $r = -0.16$; N = 998; $P < .0001$) and mental component score (Pearson $r = -0.20$; N = 1089; $P < .0001$) [Information received from the Authors].	The age-adjusted prevalence of diarrhea was higher in women than in men of ≥65 years of age ($P = 0.007$). Diarrhea was not significantly associated with a decline in nutritional status evaluated with the Subjective Global Assessment tool (SGA; "at least 1 point decline in SGA at any visit during the first 12 months of follow-up"). The prevalence of diarrhea decreased over the 1-year follow-up period (about −2.4%).	NR
Yapa [16] (P, S)	Diarrhea severity did not significantly correlate with summary measures of SF-36v2 questionnaire: Physical (PCS; r=0.001, $P = 0.97$) nor Mental (MCS; r=0.030, $P = 0.38$) Component Summary. [Information from correspondence with authors].	Both prevalence ($P < 0.05$) and severity ($P < 0.001$) of diarrhea were significantly associated with the stage of CKD. Patients with CKD G3b, G4, and G5 had significantly lower severity of diarrhea than dialysis patients (all $P < 0.001$). Patients with CKD G5 have more severe diarrhea than patients with G3b ($P < 0.05$).	NR
Dawson [14] (P)	In the conservatively managed patients, there was not a significant association between taste disturbances and diarrhea prevalence ($P = 0.18$).	NR	NR
Almutary [61] (P, S)	NR	Factor analysis (principal axis factoring, oblique rotation, CKD patients including dialysis-dependent) revealed that gastrointestinal symptom cluster includes nausea and vomiting as core symptoms across all dimensions. Diarrhea is related to this cluster in distress and severity dimensions only. Non-dialysis patients with CKD G5 had lower prevalence of self-reported diarrhea than dialysis-dependent patients (2.6% vs G5D: 28.6%, $P < 0.001$).	NR
Gutiérrez Sánchez [47,49] (P, S)	NR	Factor analysis (maximum likelihood extraction, oblimin rotation; both non-dialysis and dialysis-dependent CKD G5), diarrhea is clustered together with nausea and vomiting.	NR

		Non-dialysis patients with CKD G4-5 did not significantly differ in the self-reported diarrhea prevalence from dialysis-dependent patients (13.7% vs G5D: 17.9%, *P* = 0.47).	
Sanya [24] (P)	NR	There was not significant association between reporting diarrhea and having autonomic neuropathy in CKD patients.	NR
Onodugo [40] (P)	NR	In the multivariable regression model, autonomic dysfunction was not associated with diarrhea (*P* = 0.11), but was associated with nocturnal diarrhea (Odds ratio = 29; *P* = 0.02).	NR
Abdel-Kader [45]	NR	Median severity of diarrhea was lower in non-dialysis patients than in dialysis patients (*P* = 0.04), but there was no difference in the prevalence of it between these groups (G4-5: 25% vs G5D: 28%, *P* = 0.7).	NR
Senanayake [13] (P)	NR	Non-dialysis patients with CKD G5 had lower prevalence of self-reported diarrhea than dialysis-dependent patients (4% vs G5D: 16%, *P* = 0.016).	NR
Ducharlet [39] (P)	NR	Patients with CKD G4 had lower prevalence of diarrhea in comparison to dialysis patients (42% vs 64%; *P* = 0.03).	NR
Wan Zukiman [46] (P)	NR	Non-dialysis patients with CKD G5 did not significantly differ in the self-reported diarrhea prevalence from dialysis-dependent patients (20% vs G5D: 10%, *P* = 0.11).	NR
Ariffin [22] (P)	NR	Non-dialysis patients with CKD G5 did not significantly differ in the self-reported diarrhea prevalence from healthy controls (8% vs 4%, *P* = 0.30) or dialysis-dependent patients (8% vs 8.3%, *P* = 0.94).	NR
Taira [56] (P)	NR	Non-dialysis patients with CKD of unknown etiology (eGFR not reported) G5 did not significantly differ in the self-reported diarrhea prevalence from their healthy family members and neighbors (13.3% vs 2.6%, *P* = 0.07).	NR
Murtagh [51,52] (P)	NR	In the subgroup of patients who died, the prevalence of diarrhea within a month before death was nearly the same as in the whole baseline group [8% (95% CI: 2 to 20%)].	NR
Gordon [21] (P)	NR	NR	The composition of bile acids in the proximal small intestine after the test meal in CKD patients with diarrhea differs from the composition observed in healthy patients. Three patients with severe diarrhea had a decreased percentage of deoxycholic acid (DCA; mean 7.5%) and elevated percent of ursodeoxycholic acid (UDCA; mean 14.5%).

			Patient with severe uremia but without diarrhea had a more normal composition of bile acids (DCA: 23.7% [norms: 28±11]; UDCA: 2.4% [norms: <1]). Authors concluded: "Patients with low DCA and UDCA acid greater than 3% of total bile acids have symptoms of watery diarrhea. In this patient population, it appears that both of these bile acid abnormalities must be present for the symptom complex of diarrhea to occur".

* Outcome that was tested for association with other data. NR: not reported; P: prevalence; S: severity.

Table S16. Severity of abdominal bloating

	Total (N analyzed participants)	Mild (“A little”*)	Moderate („Somewhat”*)	Severe („Quite a lot”*)	Very severe (“very much”*)
**CKD G1-2**					
Ruszkowski	10	8	2	0	0
Prevalence (95% CI) [a]		80% (70-100)	20% (10.0-48.7)	0% (0-28.7)	0% (0-28.7)
**CKD G3**					
Ruszkowski	30	15	14	1	0
Prevalence (95% CI) [a]					
**CKD G4-5**					
Ruszkowski (%, [95% CI]) [a]	16	8 [50.0% (31.3-78.0)]	5 [31.25% (12.5-59.2)]	2 [12.5% (0-40.5)]	1 [6.25% (0-34.2)]
Murtagh (%, [95% CI]) [b]	14	12 [85.7% (57.2-98.2)]		2 [14.3% (1.8-42.8)]	
Yong	10	Mean (SD) 3.5 (1.7) in scale 0-10			

* We put answers from Murtagh *et al*. in brackets. [a] Confidence intervals for multinomial proportions were calculated according to the method of Sison and Glaz. [b] Confidence intervals for binomial proportions were calculated according to the conservative exact Clopper–Pearson method.

Table S17. Relationships between self-reported abdominal bloating and HRQoL, clinical data, or laboratory test results.

Author	HRQoL	Laboratory data	Clinical data
Ruszkowski [18,27]	Presence of bloating was associated with worse assessment of bodily pain, vitality, and mental health (SF36v2 domains).	There was no significant relationship between eGFR and either prevalence or severity of abdominal bloating.	NR
Meade [29]	NR	NR	In the combined group of non-dialysis and dialysis CKD patients, "there was no significant association of fruit, vegetables, wholegrains or legumes intake with any GI symptom" (*P* not reported).
Murtagh [51,52]	NR	NR	In the subgroup of patients who died, the prevalence of bloating within a month before death was 1.64 times higher than in the whole baseline group (35% [95% CI: 22-50%]).
Ariffin [22] (P)	NR	NR	Non-dialysis patients with CKD G5 did not significantly differ in the self-reported bloating prevalence from healthy controls (16% vs 10%, *P* = 0.29) or dialysis-dependent patients (16% vs 20.5%, *P* = 0.48).
Yong [43] (P, S)	NR	NR	Non-dialysis patients with CKD G5 did not significantly differ in the self-reported bloating prevalence from dialysis-dependent patients (22% vs G5D: 28%, *P* = 0.48). However, non-dialysis patients with CKD G5 had significantly less severe bloating than dialysis-dependent patients reporting this symptom (*P* = 0.04).

Table S18. Abdominal pain prevalence and severity in autosomal dominant polycystic kidney disease.

	Abdominal pain frequency				Abdominal pain intensity		
	Total (N analyzed participants)	never/rarely	sometimes	often/usually/always	N analyzed participants*	median (interquartile range) in males	median (interquartile range) in females
**CKD G1-2**							
Miskulin	575	420	92	63	97+175	2.0 (1.0–3.0)	2.0(1.0–3.0)
Prevalence (95% CI) [a]		73.0% (69.6-76.7)	16.0% (12.5-19.6)	11.0% (7.5-14.59)		-	-
Prevalence (95% CI) [b]			27.0% (23.4-30.8)			-	-
**CKD G3a**							
Miskulin	216	155	35	26	39+56	1.0 (1.0–3.0)	2.0 (1.0–4.0)
Prevalence (95% CI) [a]		71.8% (66.2-77.9)	16.2% (10.6-22.3)	12.0% (6.5-18.1)		-	-
Prevalence (95% CI) [b]			28.2% (22.3-34.7)			-	-
**CKD G3b-G4**							
Miskulin	204	141	33	30	47+66	2.0 (1.0–3.0)	2.0 (1.0–4.0)
Prevalence (95% CI) [a]		69.1% (63.2-75.6)	16.2% (10.3-22.7)	14.7% (8.8-21.2)		-	-
Prevalence (95% CI) [b]			30.9% (24.6-37.7)			-	-

* Number of males and females was shown. [a] Confidence intervals for multinomial proportions were calculated according to the method of Sison and Glaz. [b] Confidence intervals for binomial proportions were calculated according to the conservative exact Clopper–Pearson method.

Table S19. Relationships between self-reported abdominal pain and HRQoL, clinical data, or laboratory tests results.

Author, reference (outcome*)	HRQoL	Clinical data	Laboratory data
Ruszkowski [18,27] (P, S)	Presence of pain in the abdomen was associated with worse assessment of nearly all SF-36v2 HRQoL domains, with exception for role limitations due to emotional problems (RE, P > 0.05) and general health (GH, not assessed in the cited study). Patients reporting abdominal pain more frequently had impaired sleep quality in comparison to those without the symptom (PR 5.33; 95% CI 2.22- 12.79; P < 0.001). The higher severity of abdominal pain, the higher prevalence ratio of impaired sleep quality in comparison to patients without the symptom: mild pain is associated with PR 4.24 (95% CI 1.56-11.52) and at least moderate pain with PR 7.20 (95% CI 2.87-18.03). After adjustment for key clinical data, the associations remained significant (mild: PR 2.91, 95% CI 1.19-7.15; at least moderate: PR 11.04, 95% CI 4.82-25.26).	NR	NR
Miskulin & Nowak, [33,34] (P, S)	NR	"Among patients with eGFR >60 mL/min/1.73 m2, htTKV was not related to the frequency or intensity of abdominal pain in females or males". In contrast to back/radicular pain, there was no significant difference in odds of abdominal pain according to BMI category [normal, overweight, obese). Change in weight during the study was not associated with the significant change in abdominal pain.	"The intensity of (...) abdominal pain on average or at their worst (data not shown) was also not associated with eGFR"
Ariffin [22] (P)	NR	Non-dialysis patients with CKD G5 did not significantly differ in the self-reported abdominal pain prevalence from healthy controls (12% vs 13%, *P* = 0.86) or dialysis-dependent patients (12% vs 16.7%, *P* = 0.43).	NR

* Outcome that was tested for association with other data. NR: not reported; P: prevalence; S: severity.

Table S20. Relationships between stool consistency and HRQoL, clinical data, or laboratory tests results.

Author, reference	HRQoL	Laboratory data	Clinical data
Ruszkowski, [18,27]	Reporting types 1-2 of stool consistency was associated with neither worse HRQoL (any domain) nor altered sleep quality.	Prevalence of type 1-2 consistency was not significantly different between eGFR terciles (*P* values between 0.25 and 0.59).	Besides female sex and increasing age, taking diuretics was independently associated with increased prevalence of reporting type 1-2 stool form (adjusted PR 2.86, 95% CI 1.28-6.37, *P* = 0.01).
Ramos, [17]	NR	Patients with BSS<3 had significantly higher levels of all fractions (serum total, serum free, urinary) of *p*-cresyl sulfate (PCS) in comparison to patients with BSS ≥ 3. "In the multivariate analysis, the association of BSS<3 with PCS was maintained after adjustments for eGFR and protein-fiber ratio".	"No differences were found in gender, age, body mass index, and dietary parameters between constipated and non-constipated groups assessed by (…) BSS" (*P* value not reported).
Gryp, [20,32]	NR	"Fecal dry weight percentage significantly correlated with Bristol stool scale (*P* < 0.001, rs = –0.579)". There were significant negative correlations between BSS and plasma hippuric acid in the total CKD cohort (rs = −0.343, *P* < 0.001) and in stages G1–2 (rs = 0.366, *P* = 0.036); and between BSS and *p*-cresyl sulfate in the total CKD cohort (rs = −0.287, *P* = 0.003) and stages G4–5 (rs = 0.443, *P* = 0.012). Moreover, BSS correlated with the Bray-Curtis-based variation of the microbial composition of stool microbiome.	NR
Meade, [29]	NR	NR	In the combined group of non-dialysis and dialysis CKD patients, "there was no significant association of fruit, vegetables, wholegrains or legumes intake with (...) stool consistency" (*P* value not reported).

BSS: Bristol stool scale

Table S21. Number of bowel movements per week in patients with CKD or diabetic kidney disease.

	Total (N analyzed)	BM < 3	3 <= BM < 7	BM = 7	BM > 7
**CKD G1-2**					
Ruszkowski [18,27]	16	2	4	5	5
Prevalence (95% CI) [a]		12.5% (0-41.3)	25.0% (6.3-53.8)	31.25% (12.5-60.1)	31.25% (12.5-60.1)
**CKD G3**					
Ruszkowski [18,27]	67	5	17	34	11
Prevalence (95% CI) [a]		7.5% (0-20.8)	25.4% (14.9- 38.8)	50.7% (40.3-64.1)	16.4% (6.0-29.8)
**CKD G4-5**					
Meade [29]	134	2	20	63	49
Ruszkowski [18,27]	23	0	10	7	6
Prevalence (95% CI) [b]		1.5% (0-14.7)	26.1% (1.8-58.5)	40.5% (9.4-72.4)	31.9% (4.4-64.4)
**CKD eGFR undetermined**					
Trimingham [23]	99	0	11	52	36
Prevalence (95% CI) [a]		0% (0-10.9)	11.1% (2-22.1)	52.5% (43.4-63.5)	36.4% (27.3-47.3)
**DKD**					
Ohkuma (DKD A2-3) [26]	1880	176	571	1133	
Prevalence (95% CI) [a]		9.4% (7.1-11.7)	30.4% (28.1- 32.7)	60.3% (58.0-62.6)	
Ohkuma (DKD G3-5) [26]	1012	96	333	583	
Prevalence (95% CI) [a]		9.5% (6.3-12.7)	32.9% (29.7-36.1)	57.6% (54.4-60.8)	

BM: bowel movements per week; DKD: diabetic kidney disease. [a] Only one study provided data, thus meta-analysis could not be performed. Confidence intervals for multinomial proportions were calculated according to the method of Sison and Glaz. [b] Meta-analysis was conducted using a random-effects model with Freeman–Tukey double arcsine transformation of proportions (I^2 = 88%).

Table S22. Relationships between the frequency of defecations and HRQoL, clinical data, or laboratory tests results.

Authors, reference	HRQoL	Laboratory data	Clinical data
Ruszkowski [18,27]	Defecation less frequently than mean once a day was significantly associated with worse assessment of physical functioning, role limitations due to physical health problems, and mental health. Additionally, having less than 7 BM/week was associated with the increased prevalence ratio of impaired sleep quality (PR 7.23, 95% CI 1.74-30.12, *P* = 0.007) in comparison to having 7 BM/week. After adjustment for key clinical data, the association remained significant (PR 4.64, 95% CI 1.13-18.97, *P* = 0.03).	NR	NR
Ohkuma [26]	NR	In patients with diabetes type 2, the likelihood of both decreased eGFR (< 60 ml/min/1.73 m^2) and albuminuria (> 30 mg/g) was significantly higher in participants with less than 3 defecations per week compared with those without, with multivariable-adjusted ORs of 1.58 (1.19–2.09) and 1.49 (1.17-1.90), respectively.	NR
Ohkuma [26]	NR	In patients with diabetes type 2, the likelihood of decreased eGFR (< 60 ml/min/1.73 m^2) was significantly higher in participants with less than 7 but at least 3 defecations per week compared with those without, with multivariable-adjusted OR of 1.37 (1.16–1.62). The likelihood of albuminuria did not significantly differed between groups.	NR
Meade [29]	NR	NR	In the combined group of non-dialysis and dialysis CKD patients, "there was no significant association of fruit, vegetables, wholegrains or legumes intake with (...) stool frequency" (*P* not reported).

Table S23. Sensitivity analysis: differences from the reference model exceeding one percent.

Outcome	Subgroup	Model	Overall proportion (prevalence)			Difference in proportion
			Point estimate	95% CI: LL	95% CI: UL	
Self-reported diarrhea	G4-5	cauchit	0.1533	0.1173	0.2167	-0.0244
Self-reported abdominal pain	G4-5	cauchit	0.1724	0.0898	0.5401	-0.0232
Functional constipation	G3	FTT (ML, harmonic)	0.1543	0.0714	0.2557	-0.0191
Functional constipation	G3	FTT (REML, harmonic)	0.1543	0.0714	0.2557	-0.0191
Self-reported abdominal bloating	G4-5	cauchit	0.3467	0.2475	0.4906	-0.0147
Self-reported diarrhea	G3	cauchit	0.1240	0.0857	0.2153	-0.0134
Self-reported diarrhea	G1-2	FTT (ML, harmonic)	0.1258	0.0283	0.2607	-0.0127
Self-reported constipation	G4-5	cauchit	0.3747	0.3033	0.4620	-0.0130
Self-reported abdominal pain	G4-5	FTT (ML, harmonic)	0.2075	0.0619	0.4023	0.0120
Self-reported abdominal bloating	G1-2	FTT (REML, harmonic)	0.4971	0.4010	0.5933	0.0127
Self-reported abdominal bloating	G1-2	FTT (REML, inverse var)	0.4972	0.4032	0.5913	0.0127
Self-reported diarrhea	G4-5	FTT (ML, inverse var)	0.1907	0.1437	0.2425	0.0130
Self-reported abdominal pain	G4-5	FTT (REML, harmonic)	0.2085	0.0386	0.4525	0.0130
Self-reported diarrhea	G4-5	FTT (REML, inverse var)	0.1909	0.1427	0.2441	0.0131
Self-reported diarrhea	G1-2	FTT (ML, inverse var)	0.1572	0.0623	0.2816	0.0186
Self-reported diarrhea	G1-2	FTT (REML, inverse var)	0.1658	0.0403	0.3429	0.0273

Supplementary Figures

Figure S1. Funnel and Doi plot for self-reported constipation in chronic kidney disease (CKD) G4–5

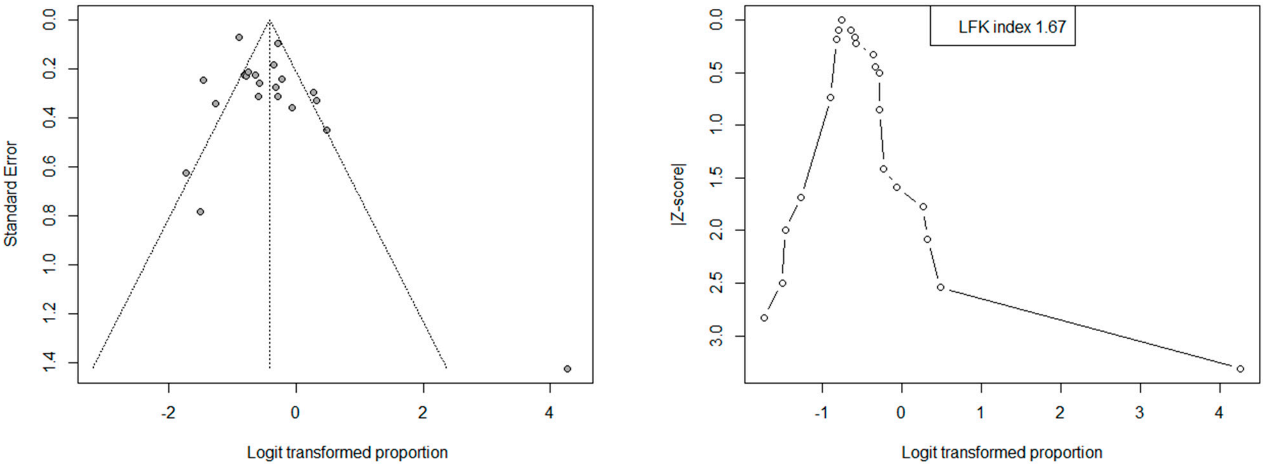

Figure S2. Forest plot with pooled odds ratio for self-reported constipation in CKD (G4–5 vs G3)

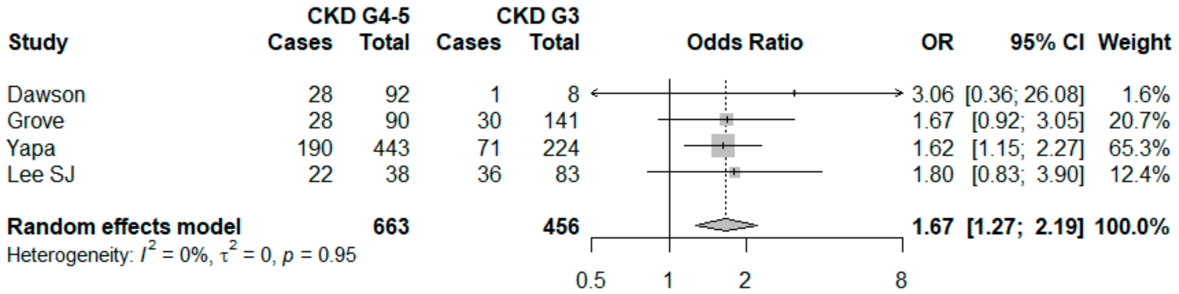

Random effects model with restricted maximum-likelihood (REML) estimator for τ^2. The weighted pooled OR significantly differs from 1 ($P < 0.001$).

Figure S3. Bubble plot based on meta-regression model: odds ratio for self-reported constipation in CKD (G4–5 vs G3)

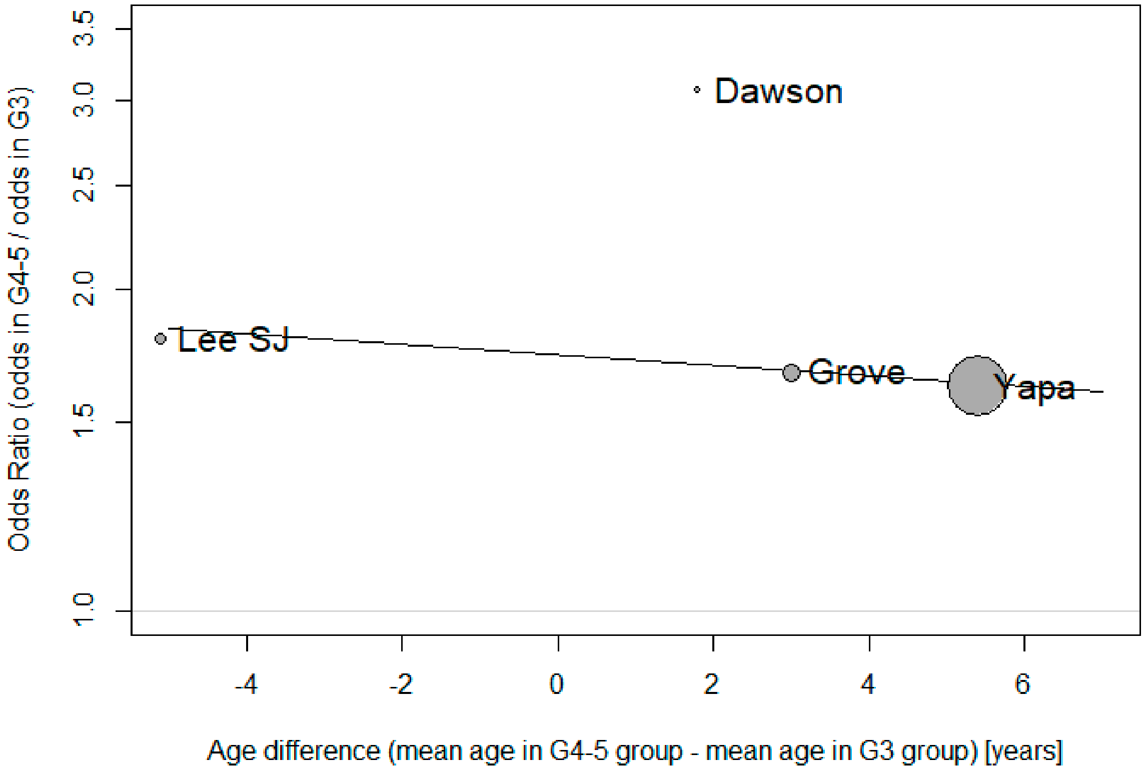

Meta-regression conducted using mixed-effects model (k = 4; τ^2 estimator: REML, R^2 = 0%). Estimated effect of the age difference: -0.0114 [95% CI: -0.0916 to 0.0688], *P* = 0.78.

Figure S4. Funnel and Doi plot for self-reported diarrhea in CKD G4–5

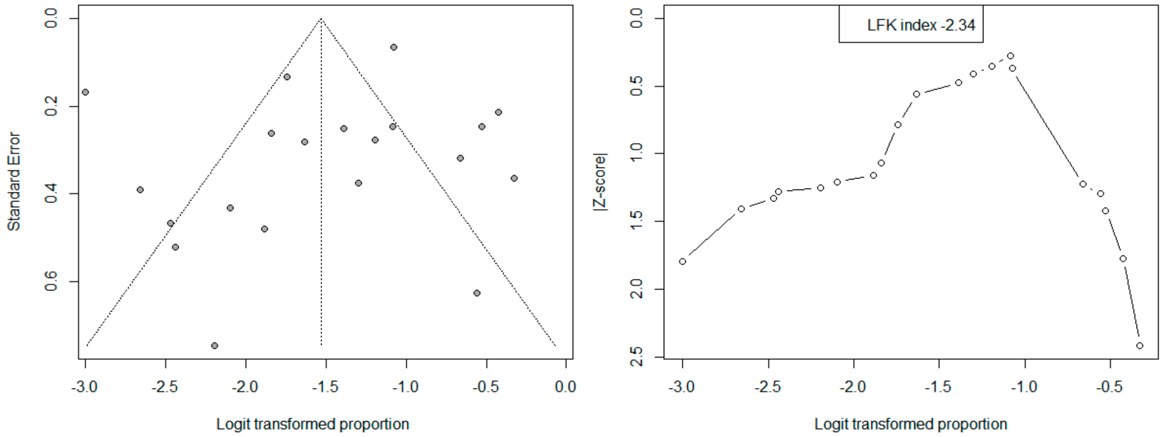

Figure S5. Forest plot with pooled odds ratio for self-reported diarrhea in CKD (G3 vs G1-2)

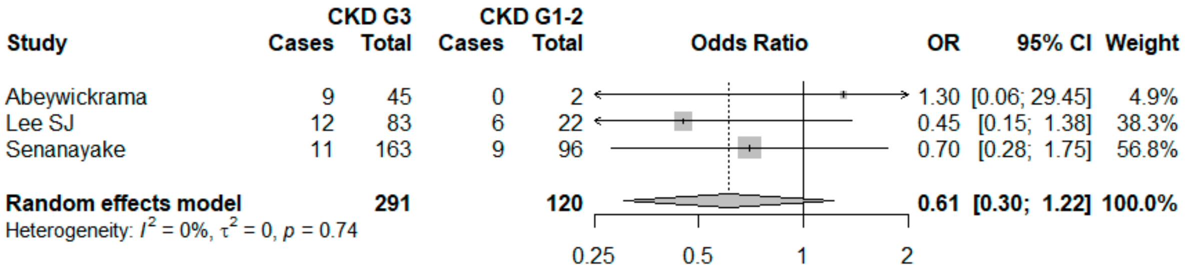

Random effects model with restricted maximum-likelihood (REML) estimator for τ^2. The weighted pooled OR does not significantly differ from 1 (P = 0.16).

Figure S6. Forest plot with pooled odds ratio for self-reported diarrhea in CKD (G4-5 vs G3)

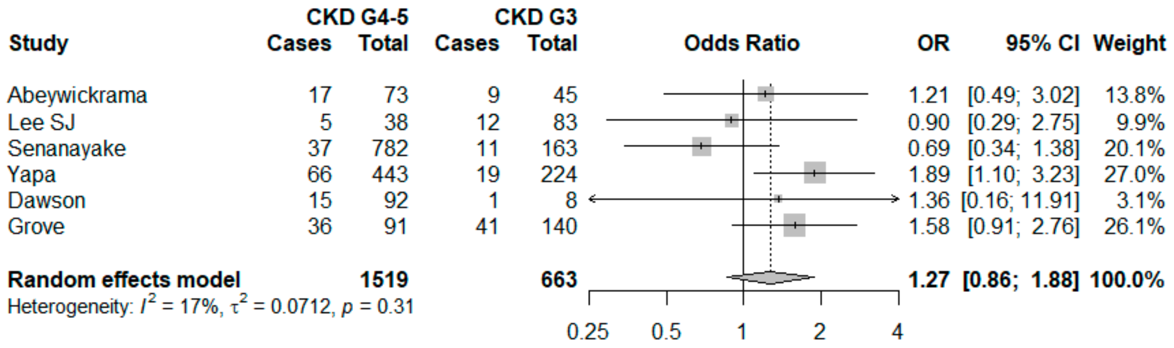

Random effects model with restricted maximum-likelihood (REML) estimator for τ^2. The weighted pooled OR does not significantly differ from 1 (P = 0.23).

Figure S7. Forest plot with pooled odds ratio for self-reported abdominal bloating in CKD (G3 vs G1–2)

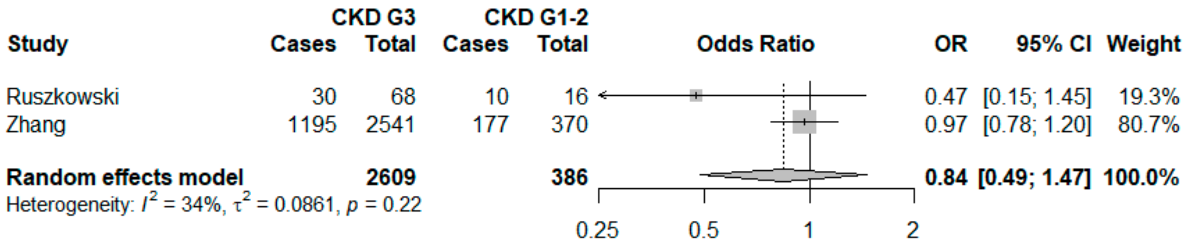

Random effects model with restricted maximum-likelihood (REML) estimator for τ^2. The weighted pooled OR does not significantly differ from 1 (P = 0.55).

Figure S8. Forest plot with pooled odds ratio for self-reported abdominal bloating in CKD (G4–5 vs G3)

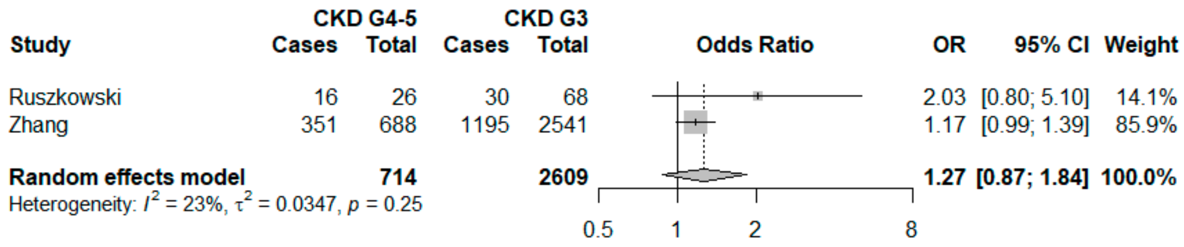

Random effects model with restricted maximum-likelihood (REML) estimator for τ^2. The weighted pooled OR does not significantly differ from 1 (*P* = 0.21).

Supplementary References

1. Furuya-Kanamori, L.; Barendregt, J.J.; Doi, S.A.R. A New Improved Graphical and Quantitative Method for Detecting Bias in Meta-Analysis. *Int. J. Evid. Based. Healthc.* **2018**, *16*, 195–203, doi:10.1097/XEB.0000000000000141.

2. Peters, J.L.; Sutton, A.J.; Jones, D.R.; Abrams, K.R.; Rushton, L. Comparison of Two Methods to Detect Publication Bias in Meta-Analysis. *JAMA* **2006**, *295*, 676–680, doi:10.1001/JAMA.295.6.676.

3. Balduzzi, S.; Rücker, G.; Schwarzer, G. How to Perform a Meta-Analysis with R: A Practical Tutorial. *Evid. Based. Ment. Health* **2019**, *22*, 153–160, doi:10.1136/ebmental-2019-300117.

4. Schwarzer, G.; Carpenter, J.R.; Rücker, G. Metasens: Statistical Methods for Sensitivity Analysis in Meta-Analysis 2022.

5. Sison, C.P.; Glaz, J. Simultaneous Confidence Intervals and Sample Size Determination for Multinomial Proportions. *J. Am. Stat. Assoc.* **1995**, *90*, 366–369, doi:10.1080/01621459.1995.10476521.

6. Hilton Boon, M.; Thomson, H.; Shaw, B.; Akl, E.A.; Lhachimi, S.K.; López-Alcalde, J.; Klugar, M.; Choi, L.; Saz-Parkinson, Z.; Mustafa, R.A.; et al. Challenges in Applying the GRADE Approach in Public Health Guidelines and Systematic Reviews: A Concept Article from the GRADE Public Health Group. *J. Clin. Epidemiol.* **2021**, *135*, 42–53, doi:10.1016/j.jclinepi.2021.01.001.

7. Guyatt, G.H.; Oxman, A.D.; Vist, G.E.; Kunz, R.; Falck-Ytter, Y.; Alonso-Coello, P.; Schünemann, H.J. GRADE: An Emerging Consensus on Rating Quality of Evidence and Strength of Recommendations. *BMJ* **2008**, *336*, 924–926, doi:10.1136/BMJ.39489.470347.AD.

8. Lin, L.; Xu, C.; Chu, H. Empirical Comparisons of 12 Meta-Analysis Methods for Synthesizing Proportions of Binary Outcomes. *J. Gen. Intern. Med.* **2022**, *37*, 308, doi:10.1007/S11606-021-07098-5.

9. Schwarzer, G.; Chemaitelly, H.; Abu-Raddad, L.J.; Rücker, G. Seriously Misleading Results Using Inverse of Freeman-Tukey Double Arcsine Transformation in Meta-Analysis of Single Proportions. *Res. Synth. Methods* **2019**, *10*, 476–483, doi:10.1002/JRSM.1348.

10. Borges Migliavaca, C.; Stein, C.; Colpani, V.; Barker, T.H.; Munn, Z.; Falavigna, M. How Are Systematic Reviews of Prevalence Conducted? A Methodological Study. *BMC Med. Res. Methodol.* **2020**, *20*, 1–9, doi:10.1186/s12874-020-00975-3.

11. Abeywickrama, H.M.; Wimalasiri, S.; Koyama, Y.; Uchiyama, M.; Shimizu, U.; Kakihara, N.; Chandrajith, R.; Nanayakkara, N. Quality of Life and Symptom Burden among Chronic Kidney Disease of Uncertain Etiology (CKDU) Patients in Girandurukotte, Sri Lanka. *Int. J. Environ. Res. Public Health* **2020**, *17*, 1–16, doi:10.3390/ijerph17114041.

12. Lee, S.J.; Jeon, J.H. Relationship between Symptom Clusters and Quality of Life in Patients at Stages 2 to 4 Chronic Kidney Disease in Korea. *Appl. Nurs. Res.* **2015**, *28*, e13–e19, doi:10.1016/J.APNR.2015.03.004.

13. Senanayake, S.; Gunawardena, N.; Palihawadana, P.; Bandara, P.; Haniffa, R.; Karunarathna, R.; Kumara, P. Symptom Burden in Chronic Kidney Disease; a Population Based Cross Sectional Study. *BMC Nephrol.* **2017**, *18*, 228, doi:10.1186/s12882-017-0638-y.

14. Dawson, J.; Brennan, F.P.; Hoffman, A.; Josland, E.; Li, K.C.; Smyth, A.; Brown, M.A. Prevalence of Taste Changes and Association with Other Nutrition-Related Symptoms in End-Stage Kidney Disease Patients. *J. Ren. Nutr.* **2021**, *31*, 80–84, doi:10.1053/J.JRN.2020.06.003.

15. Grove, B.E.; Schougaard, L.M.V.; Ivarsen, P.R.; Kyte, D.; Hjollund, N.H.; de Thurah, A. Patient-Reported Outcome Measures for Clinical Decision-Making in Outpatient Follow-up: Validity and Reliability of a Renal Disease Questionnaire. *J. Patient-Reported Outcomes* **2021**, *5*, 107, doi:10.1186/s41687-021-00384-0.

16. Yapa, H.E.; Purtell, L.; Chambers, S.; Bonner, A. Alterations in Symptoms and Health-Related Quality of Life as Kidney Function Deteriorates: A Cross-Sectional Study. *J. Clin. Nurs.* **2021**, *30*, 1787–1796,

doi:10.1111/JOCN.15738.

17. Ramos, C.I.; Armani, R.G.; Canziani, M.E.; Ribeiro Dolenga, C.J.; Nakao, L.S.; Campbell, K.L.; Cuppari, L. Bowel Habits and the Association With Uremic Toxins in Non–Dialysis-Dependent Chronic Kidney Disease Patients. *J. Ren. Nutr.* **2020**, *30*, 31–35, doi:10.1053/j.jrn.2019.02.004.

18. Ruszkowski, J.; Heleniak, Z.; Król, E.; Tarasewicz, A.; Gałgowska, J.; Witkowski, J.M.; Dębska-Ślizień, A. Constipation and the Quality of Life in Conservatively Treated Chronic Kidney Disease Patients: A Cross-Sectional Study. *Int. J. Med. Sci.* **2020**, *17*, 2954–2963, doi:10.7150/ijms.49648.

19. Zhang, X.; Bansal, N.; Go, A.S.; Hsu, C. Gastrointestinal Symptoms, Inflammation and Hypoalbuminemia in Chronic Kidney Disease Patients: A Cross-Sectional Study. *BMC Nephrol.* **2015**, *16*, 211, doi:10.1186/s12882-015-0209-z.

20. Gryp, T.; De Paepe, K.; Vanholder, R.; Kerckhof, F.M.; Van Biesen, W.; Van de Wiele, T.; Verbeke, F.; Speeckaert, M.; Joossens, M.; Couttenye, M.M.; et al. Gut Microbiota Generation of Protein-Bound Uremic Toxins and Related Metabolites Is Not Altered at Different Stages of Chronic Kidney Disease. *Kidney Int.* **2020**, *97*, 1230–1242, doi:10.1016/J.KINT.2020.01.028.

21. Gordon, S.J.; Miller, L.J.; Haeffner, L.J.; Kinsey, M.D.; Kowlessar, O.D. Abnormal Intestinal Bile Acid Distribution in Azotaemic Man: A Possible Role in the Pathogenesis of Uraemic Diarrhoea. *Gut* **1976**, *17*, 58–67, doi:10.1136/GUT.17.1.58.

22. Muhd Ariffin, N.F.; Naing, L.; Pisharam, J.; Maboud Khalil, M.A.; Tamin, N.; Chong, V.H.; Tan, J. Appetite and Gastrointestinal Symptoms in End Stage Renal Disease Patients. *J. Clin. Exp. Nephrol.* **2016**, *1*, doi:10.21767/2472-5056.100006.

23. Trimingham, C.; McDonald, S.; Dansie, K.; Jesudason, S.; Faull, R.; Clayton, P.; Liew, G.; Le Leu, R.; Meade, A. Bowel Health in Chronic Kidney Disease: Patient Perceptions Differ from Clinical Definitions. *J. Ren. Care* **2018**, *44*, 65–72, doi:10.1111/JORC.12230.

24. Sanya, E.O.; Ogunniyi, A. Cardiovascular Autonomic Neuropathy in Non-Diabetic Nigerian Patients with Chronic Renal Failure. *West Afr. J. Med.* **2004**, *23*, 15–20, doi:10.4314/WAJM.V23I1.28073.

25. Saini, T.; Murtagh, F.E.M.; Dupont, P.J.; McKinnon, P.M.; Hatfield, P.; Saunders, Y. Comparative Pilot Study of Symptoms and Quality of Life in Cancer Patients and Patients with End Stage Renal Disease. *Palliat. Med.* **2006**, *20*, 631–636, doi:10.1177/0269216306070236.

26. Ohkuma, T.; Iwase, M.; Fujii, H.; Ide, H.; Komorita, Y.; Yoshinari, M.; Oku, Y.; Higashi, T.; Oshiro, A.; Nakamura, U.; et al. Constipation and Diabetic Kidney Disease: The Fukuoka Diabetes Registry. *Clin. Exp. Nephrol.* **2021**, *25*, 1247–1254, doi:10.1007/S10157-021-02105-9.

27. Ruszkowski, J.; Heleniak, Z.; Król, E.; Tarasewicz, A.; Witkowski, J.M.; Dębska-Ślizień, A. Associations between Constipation Symptoms and the Sleep Quality in Non-Dialysis Chronic Kidney Disease Patients: A Cross-Sectional Study. *Polish Arch. Intern. Med.* **2021**, *131*, 512–519, doi:10.20452/pamw.15974.

28. Quintal-Medina, I.A.; Rocha-González, H.I.; Noyola-García, M.E.; Flores-Padilla, M.G. Factores Asociados a Carga Sintomática Residual En Pacientes Con Diálisis Peritoneal: Estudio de Cohorte [Factors Associated with Residual Symptom Burden in Patients with Peritoneal Dialysis: A Cohort Study]. *Rev. Med. Inst. Mex. Seguro Soc.* **2020**, *58*, 174–180, doi:10.24875/RMIMSS.M20000015.

29. Meade, A.; Le Leu, R.; Watson, N.; Jesudason, S.; Clayton, P.; Faull, R.; McDonald, S.; Trimingham, C. Gastrointestinal Symptom Burden and Dietary Intake in Patients with Chronic Kidney Disease. *J. Ren. Care* **2021**, *47*, 234–241, doi:10.1111/JORC.12373.

30. Wizemann, V.; Benz, U. Gastrointestinale Befunde Bei Chronisch Niereninsuffizienten Patienten [Gastrointestinal Symptoms in Patients Suffering from Chronic Uremia]. *Med. Welt* **1978**, *29*, 1025–1029.

31. Lash, J.P.; Go, A.S.; Appel, L.J.; He, J.; Ojo, A.; Rahman, M.; Townsend, R.R.; Xie, D.; Cifelli, D.; Cohan, J.; et al. Chronic Renal Insufficiency Cohort (CRIC) Study: Baseline Characteristics and Associations with Kidney Function. *Clin. J. Am. Soc. Nephrol.* **2009**, *4*, 1302–1311, doi:10.2215/CJN.00070109.

32. Gryp, T.; Faust, K.; Van Biesen, W.; Huys, G.R.B.; Verbeke, F.; Speeckaert, M.; Raes, J.; Vaneechoutte, M.; Joossens, M.; Glorieux, G. Gut Microbiome Profiling Uncovers a Lower Abundance of Butyricicoccus in Advanced Stages of Chronic Kidney Disease. *J. Pers. Med.* **2021**, *11*, 1118, doi:10.3390/jpm11111118.

33. Miskulin, D.C.; Abebe, K.Z.; Chapman, A.B.; Perrone, R.D.; Steinman, T.I.; Torres, V.E.; Bae, K.T.; Braun, W.; Winklhofer, F.T.; Hogan, M.C.; et al. Health-Related Quality of Life in Patients with Autosomal Dominant Polycystic Kidney Disease and CKD Stages 1-4: A Cross-Sectional Study. *Am. J. Kidney Dis.* **2014**, *63*, 214–226, doi:10.1053/J.AJKD.2013.08.017.

34. Nowak, K.L.; Murray, K.; You, Z.; Gitomer, B.; Brosnahan, G.; Abebe, K.Z.; Braun, W.; Chapman, A.; Harris, P.C.; Miskulin, D.; et al. Pain and Obesity in Autosomal Dominant Polycystic Kidney Disease: A Post Hoc Analysis of the Halt Progression of Polycystic Kidney Disease (HALT-PKD) Studies. *Kidney Med.* **2021**, *3*, 536-545.e1, doi:10.1016/J.XKME.2021.03.004.

35. Windahl, K.; Irving, G.F.; Almquist, T.; Lidén, M.K.; Stenvinkel, P.; Chesnaye, N.C.; Drechsler, C.; Szymczak, M.; Krajewska, M.; Fu, E.L.; et al. Patient-Reported Measures and Lifestyle Are Associated With Deterioration in Nutritional Status in CKD Stage 4-5: The EQUAL Cohort Study. *J. Ren. Nutr.* **2022**, *32*, 161–169, doi:10.1053/j.jrn.2021.03.006.

36. van de Luijtgaarden, M.W.M.; Caskey, F.J.; Wanner, C.; Chesnaye, N.C.; Postorino, M.; Janmaat, C.J.; Rao, A.; Torino, C.; Klinger, M.; Drechsler, C.; et al. Uraemic Symptom Burden and Clinical Condition in Women and Men of ≥65 Years of Age with Advanced Chronic Kidney Disease: Results from the EQUAL Study. *Nephrol. Dial. Transplant.* **2019**, *34*, 1189–1196, doi:10.1093/NDT/GFY155.

37. Janmaat, C.J.; van Diepen, M.; Meuleman, Y.; Chesnaye, N.C.; Drechsler, C.; Torino, C.; Wanner, C.; Postorino, M.; Szymczak, M.; Evans, M.; et al. Kidney Function and Symptom Development over Time in Elderly Patients with Advanced Chronic Kidney Disease: Results of the EQUAL Cohort Study. *Nephrol. Dial. Transplant.* **2021**, *36*, 862–870, doi:10.1093/NDT/GFZ277.

38. Voskamp, P.W.M.; Van Diepen, M.; Evans, M.; Caskey, F.J.; Torino, C.; Postorino, M.; Szymczak, M.; Klinger, M.; Wallquist, C.; Van De Luijtgaarden, M.W.M.; et al. The Impact of Symptoms on Health-Related Quality of Life in Elderly Pre-Dialysis Patients: Effect and Importance in the EQUAL Study. *Nephrol. Dial. Transplant* **2019**, *34*, 1707–1715, doi:10.1093/NDT/GFY167.

39. Ducharlet, K.; Sundararajan, V.; Philip, J.; Weil, J.; Barker, N.; Langham, R.G.; Burchell, J.; Gock, H. Patient-Reported Outcome Measures and Their Utility in the Management of Patients with Advanced Chronic Kidney Disease. *Nephrology (Carlton).* **2019**, *24*, 814–818, doi:10.1111/NEP.13509.

40. Onodugo, O.D.; Ulasi, I.I.; Ijoma, C.K.; Arodiwe, E.B.; Okoye, J.U.; Ezeala-Adikaibe, B.A.; Onodugo, N.P.; Ugwu, E.O. Predictors of Autonomic Dysfunction among Predialysis Chronic Kidney Disease Patients in Nigeria. *Niger. J. Clin. Pract.* **2018**, *21*, 932–938, doi:10.4103/NJCP.NJCP_369_17.

41. Allawi, A.A. dyab Prediction of Autonomic Neuropathy in Chronic Kidney Disease (Stage 5) Iraqi Patients (Case Control Study). *Diabetes Metab. Syndr. Clin. Res. Rev.* **2017**, *11*, S969–S973, doi:10.1016/j.dsx.2017.07.024.

42. Lee, A.; Lambert, K.; Byrne, P.; Lonergan, M. Prevalence of Constipation in Patients with Advanced Kidney Disease. *J. Ren. Care* **2016**, *42*, 144–149, doi:10.1111/jorc.12157.

43. Yong, D.S.P.; Kwok, A.O.L.; Wong, D.M.L.; Suen, M.H.P.; Chen, W.T.; Tse, D.M.W. Symptom Burden and Quality of Life in End-Stage Renal Disease: A Study of 179 Patients on Dialysis and Palliative Care. *Palliat. Med.* **2009**, *23*, 111–119, doi:10.1177/0269216308101099.

44. Senanayake, S.; Gunawardena, N.; Palihawadana, P.; Senanayake, S.; Karunarathna, R.; Kumara, P.; Kularatna, S. Health Related Quality of Life in Chronic Kidney Disease; A Descriptive Study in a Rural Sri Lankan Community Affected by Chronic Kidney Disease. *Health Qual. Life Outcomes* **2020**, *18*, 1–9, doi:10.1186/S12955-020-01369-1/TABLES/5.

45. Abdel-Kader, K.; Unruh, M.L.; Weisbord, S.D. Symptom Burden, Depression, and Quality of Life in Chronic and End-Stage Kidney Disease. *Clin. J. Am. Soc. Nephrol.* **2009**, *4*, 1057–1064, doi:10.2215/CJN.00430109.

46. Wan Zukiman, W.Z.H.; Yaakup, H.; Zakaria, N.F.; Shah, S.A. Bin Symptom Prevalence and the Negative Emotional States in End-Stage Renal Disease Patients with or without Renal Replacement Therapy: A Cross-Sectional Analysis. *J. Palliat. Med.* **2017**, *20*, 1127–1134, doi:10.1089/JPM.2016.0450.

47. Sánchez, D.G.; Leiva-Santos, J.P.; López, M.J.M.; Cuesta Vargas, A.I. Perfil Sintomático de Los Pacientes Con Enfermedad Renal Crónica Estudio 4 y 5 [Symptomatic Profile of Patients with Chronic Kidney Disease Stage 4 and 5]. *Enferm. Nefrol.* **2017**, *20*, 259–266, doi:10.4321/S2254-28842017000300010.

48. Gutiérrez Sánchez, D.; Leiva-Santos, J.P. Symptom Burden, Comorbidity and Functional Status of Patients with Chronic Kidney Disease Stage 5 Managed Conservately [Síntomas, Comorbilidad y Estado Funcional de Los Pacientes Con Enfermedad Renal Crónica Estadio 5 En Manejo Renal Conservador]. *Enfermería Glob.* **2021**, *20*, 44, doi:10.6018/EGLOBAL.449531.

49. Gutiérrez Sánchez, D.; Leiva-Santos, J.P.; Cuesta-Vargas, A.I. Symptom Burden Clustering in Chronic Kidney Disease Stage 5. *Clin. Nurs. Res.* **2019**, *28*, 583–601, doi:10.1177/1054773817740671.

50. Gutiérrez Sánchez, D.; Leiva-Santos, J.P.; Macías López, M.J.; Cuesta-Vargas, A.I. Prevalencia de Síntomas En Enfermedad Renal Crónica Avanzada [Prevalence of Symptoms in Advanced Chronic Kidney Disease]. *Nefrología* **2018**, *38*, 560–562, doi:10.1016/J.NEFRO.2017.11.021.

51. Murtagh, F.E.M.; Addington-Hall, J.M.; Edmonds, P.M.; Donohoe, P.; Carey, I.; Jenkins, K.; Higginson, I.J. Symptoms in Advanced Renal Disease: A Cross-Sectional Survey of Symptom Prevalence in Stage 5 Chronic Kidney Disease Managed without Dialysis. *J. Palliat. Med.* **2007**, *10*, 1266–1276, doi:10.1089/JPM.2007.0017.

52. Murtagh, F.E.; Addington-Hall, J.; Edmonds, P.; Donohoe, P.; Carey, I.; Jenkins, K.; Higginson, I.J. Symptoms in the Month before Death for Stage 5 Chronic Kidney Disease Patients Managed without Dialysis. *J. Pain Symptom Manage.* **2010**, *40*, 342–352, doi:10.1016/J.JPAINSYMMAN.2010.01.021.

53. Turkmen, K.; Guclu, A.; Sahin, G.; Kocyigit, I.; Demirtas, L.; Erdur, F.M.; Sengül, E.; Ozkan, O.; Emre, H.; Turgut, F.; et al. The Prevalence of Fabry Disease in Patients with Chronic Kidney Disease in Turkey: The TURKFAB Study. *Kidney Blood Press. Res.* **2016**, *41*, 1016–1024, doi:10.1159/000452605.

54. Brennan, F.; Collett, G.; Josland, E.A.; Brown, M.A. The Symptoms of Patients with CKD Stage 5 Managed without Dialysis. *Prog. Palliat. Care* **2015**, *23*, 267–273, doi:10.1179/1743291X14Y.0000000118.

55. Murphy, E.L.; Murtagh, F.E.M.; Carey, I.; Sheerin, N.S. Understanding Symptoms in Patients with Advanced Chronic Kidney Disease Managed without Dialysis: Use of a Short Patient-Completed Assessment Tool. *Nephron. Clin. Pract.* **2009**, *111*, doi:10.1159/000183177.

56. Taira, K.; Kawakami, T.; Weragoda, S.K.; Herath, H.M.A.S.; Ikenaka, Y.; Fujioka, K.; Hemachandra, M.; Pallewatta, N.; Aoyama, Y.; Ishizuka, M.; et al. Urinary Concentrations of Neonicotinoid Insecticides Were Related to Renal Tubular Dysfunction and Neuropsychological Complaints in Dry-Zone of Sri Lanka. *Sci. Rep.* **2021**, *11*, doi:10.1038/s41598-021-01732-2.

57. Purtell, L.; Sowa, P.M.; Berquier, I.; Scuderi, C.; Douglas, C.; Taylor, B.; Kramer, K.; Hoy, W.; Healy, H.; Bonner, A. The Kidney Supportive Care Programme: Characteristics of Patients Referred to a New Model of Care. *BMJ Support. Palliat. Care* **2018**, doi:10.1136/bmjspcare-2018-001630.

58. Sowa, P.M.; Purtell, L.; Hoy, W.E.; Healy, H.G.; Bonner, A.; Connelly, L.B. Utilization and Costs of Health Care in a Kidney Supportive Care Program. *J. Palliat. Care* **2020**, *35*, 176–184, doi:10.1177/0825859719868174.

59. de Miguel, C.; Morán, C.; Rumayor, M.; Fernández, B.; del Cura, I.; Sanz, E.M.; Gutiérrez, B. ¿Cómo Son Los Últimos Meses de Vida de Los Pacientes Con Insuficiencia Renal Crónica Avanzada En Los Que Se Desestima El Tratamiento Con Hemodiálisis o Con Diálisis Peritoneal? *Med. Paliativa* **2018**, *25*, 83–94, doi:10.1016/j.medipa.2016.08.001.

60. Almutary, H.; Bonner, A.; Douglas, C. Which Patients with Chronic Kidney Disease Have the Greatest Symptom Burden? A Comparative Study of Advanced Ckd Stage and Dialysis Modality. *J. Ren. Care* **2016**, *42*, 73–82, doi:10.1111/jorc.12152.

61. Almutary, H.; Douglas, C.; Bonner, A. Multidimensional Symptom Clusters: An Exploratory Factor Analysis in Advanced Chronic Kidney Disease. *J. Adv. Nurs.* **2016**, *72*, 2389–2400, doi:10.1111/JAN.12997.

62. Munn, Z.; Moola, S.; Lisy, K.; Riitano, D.; Tufanaru, C. Methodological Guidance for Systematic Reviews of Observational Epidemiological Studies Reporting Prevalence and Cumulative Incidence Data. *Int. J. Evid. Based. Healthc.* **2015**, *13*, 147–153, doi:10.1097/XEB.0000000000000054.
